# Cancer as a Complex Phenotype: Pattern of Cancer Distribution within and beyond the Nuclear Family

**DOI:** 10.1371/journal.pmed.0010065

**Published:** 2004-12-28

**Authors:** Laufey T Amundadottir, Sverrir Thorvaldsson, Daniel F Gudbjartsson, Patrick Sulem, Kristleifur Kristjansson, Sigurdur Arnason, Jeffrey R Gulcher, Johannes Bjornsson, Augustine Kong, Unnur Thorsteinsdottir, Kari Stefansson

**Affiliations:** **1**deCODE GeneticsReykjavikIceland; **2**National University HospitalReykjavikIceland; Guy's King's and St. Thomas' School of MedicineUnited Kingdom

## Abstract

**Background:**

The contribution of low-penetrant susceptibility variants to cancer is not clear. With the aim of searching for genetic factors that contribute to cancer at one or more sites in the body, we have analyzed familial aggregation of cancer in extended families based on all cancer cases diagnosed in Iceland over almost half a century.

**Methods and Findings:**

We have estimated risk ratios (RRs) of cancer for first- and up to fifth-degree relatives both within and between all types of cancers diagnosed in Iceland from 1955 to 2002 by linking patient information from the Icelandic Cancer Registry to an extensive genealogical database, containing all living Icelanders and most of their ancestors since the settlement of Iceland.

We evaluated the significance of the familial clustering for each relationship separately, all relationships combined (first- to fifth-degree relatives) and for close (first- and second-degree) and distant (third- to fifth-degree) relatives. Most cancer sites demonstrate a significantly increased RR for the same cancer, beyond the nuclear family. Significantly increased familial clustering between different cancer sites is also documented in both close and distant relatives. Some of these associations have been suggested previously but others not.

**Conclusion:**

We conclude that genetic factors are involved in the etiology of many cancers and that these factors are in some cases shared by different cancer sites. However, a significantly increased RR conferred upon mates of patients with cancer at some sites indicates that shared environment or nonrandom mating for certain risk factors also play a role in the familial clustering of cancer. Our results indicate that cancer is a complex, often non-site-specific disease for which increased risk extends beyond the nuclear family.

## Abstract 2

It's not often that an entire nation's genealogy and cancer records are available. But they are in Iceland, and have been used to determine how often cancers occur in families

## Introduction

Highly penetrant susceptibility variants explain only a small fraction of the genetics of all cancer cases. As an example, mutations in the *BRCA1* and *BRCA2* genes account for around 2%–3% of all breast cancer cases [1,2], although more prevalent founder mutations in these genes can explain up to about 10% of the disease in some populations [3,4,5,6,7]. However, the role of genetics in the remaining breast cancer cases and the majority of other cancers is not clear.

Family studies have given insight into the contribution of genetic and environmental factors to the etiology of cancer. Case-control, registry- and population-based studies have evaluated familial clustering using either risk ratio (RR) estimations for relatives of cancer patients, or kinship coefficient (KC) estimations for cancer patients. The largest of these studies, utilizing either the Utah Population and Cancer Registry Database or the Swedish Family-Cancer Database, have demonstrated excess familial clustering at practically all cancer sites in the body [8,9,10,11,12]. Most of these studies have been able to evaluate familial clustering only within the nuclear family, thus making it more difficult to separate the roles of shared environmental and genetic factors in the familial aggregation of cancers. However, in one of these studies [12], in which familial clustering was evaluated for more distant relatives, significant clustering outside the nuclear family was demonstrated for a number of cancer sites. Extended familial clustering has also been reported in studies of individual cancers [13,14,15,16,17,18,19,20,21,22].

Twin studies have also evaluated the role of genes versus environment in cancer susceptibility. The largest study involved close to 45,000 twins from Denmark, Sweden, and Finland where the RR of same type of cancer was calculated for individuals with affected twins and compared to those without an affected twin [23]. The authors concluded that for the majority of cancer sites only a limited part of the risk could be explained by heritable factors. Exceptions to this were cancers of the prostate, colon and breast.

In addition to well documented familial clustering for the majority of individual cancers, aggregation of different types of cancers in families has also been observed. Reports have been published on the results of systematic analysis of the aggregation of different cancers using the Utah Population and Cancer Registry Database [24,25]. In addition to demonstrating excess familial clustering for most cancer sites, these studies also indicate that an excess is also shared by different cancer sites. In these studies, cancer clustering was evaluated either by calculating the RR for first-degree relatives or KC between different cancer sites. While distant relationships contributed to the overall calculation of KC, their contributions were not evaluated separately in the studies between cancer sites, hence making it more difficult to separate the effects of genetic and environmental factors in these studies.

We have studied a registry of all cancer cases diagnosed in Iceland from 1 January 1955 to 31 December 2002, with the aim of searching for evidence of genetic factors both at individual cancer sites and those shared by different sites. By cross-referencing cancer prevalence in relatives of cases with the aid of a comprehensive nationwide genealogy database, we have estimated RR separately for first- to fifth-degree relatives of all cancer patients diagnosed in Iceland over 48 y. We demonstrate here an increased cancer risk in relatives outside the nuclear family (third- to fifth-degree relatives) for many cancer sites. These relatives share significant genetic makeup but are less likely to share environmental factors beyond those shared by the general population, indicating that genetic factors may be involved. By applying the analysis across different cancer sites we also demonstrate shared familiality between certain cancer sites both in close and distant relatives. These results suggest that cancer can be considered a broad phenotype with shared genetic factors crossing different cancer sites. That is, the difference between cancers at various sites may in part be the consequence of variable expressivity of the same cancer-predisposing genes.

## Methods

This study was approved by the National Bioethics Committee of Iceland, the Data Protection Authority of Iceland, and the Icelandic Cancer Society. All names of patients listed in the Icelandic Cancer Registry (ICR) and the genealogic database were encrypted through a process approved by the National Bioethics Committee and the Data Protection Authority before being analyzed [26].

### Cancer Registry

The ICR of the Icelandic Cancer Society is a carefully constructed database containing practically complete records of all cancer cases diagnosed in Iceland after 1 January 1955 [27]. Records are received at the ICR from all hospitals in the country that treat cancer patients, and the very few not listed are individuals who are diagnosed while living abroad. Furthermore, the records are verified by a continuous interaction between the ICR and Icelandic hospitals and clinicians. Approximately 95% of cases are histologically verified [28]. In the present study we used International Classification of Disease version 10 codes as the basis for defining phenotypes. A total of 81 unique phenotypes (sites) were analyzed. In this paper we present data from 27 sites with more than 200 cases each (Table 1). For the 48 years (1 January 1955 to 31 December 2002) a total of 32,534 individuals were found in our genealogy database. Cancer incidence in Iceland is comparable to the Nordic countries of Europe and is detailed in [27].

**Table 1:**
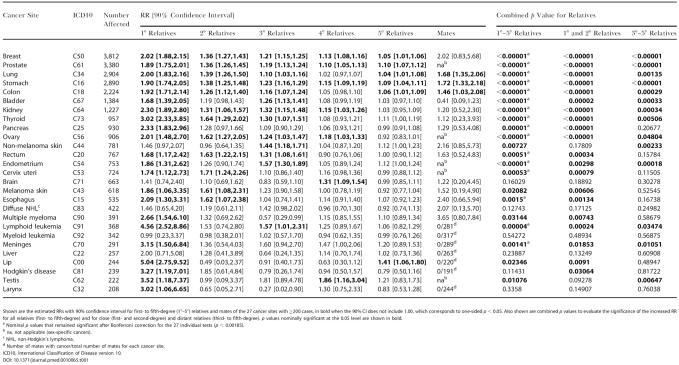
RR Estimates of Cancer at the Same Site for Relatives and Mates for Cancer Sites with 200 or More Cases

Shown are the estimated RRs with 90% confidence interval for first- to fifth-degree (1°–5°) relatives and mates of the 27 cancer sites with ≥200 cases, in bold when the 90% CI does not include 1.00, which corresponds to one-sided *p* < 0.05. Also shown are combined *p* values to evaluate the significance of the increased RR for all relatives (first- to fifth-degree) and for close (first- and second-degree) and distant relatives (third- to fifth-degree). *p* values nominally significant at the 0.05 level are shown in bold

^a^ Nominal *p* values that remained significant after Bonferroni correction for the 27 individual tests (*p* < 0.00185)

^b^ na, not applicable (sex-specific cancers)

^c^ NHL, non-Hodgkin's lymphoma

^d^ Number of mates with cancer/total number of mates for each cancer site

ICD10, International Classification of Disease version 10

### Genealogic Database

deCODE Genetics has built a computerized genealogy database of more than 687,500 individuals [29,30]. The names of all 288,000 Icelanders currently alive and a large proportion of all Icelanders who have ever lived in the country are in the database. The genealogy of the entered individuals is recorded from multiple sources including church records and censuses from previous centuries and, more recently, from published genealogy books. The genealogy database is quite complete from the 18th century on, thus allowing quite distant relationships to be traced accurately.

Mates are defined as individuals of the opposite sex who have one or more children in common, regardless of marital status.

### Calculations of RRs

The RR for relatives is a measure of the risk of disease for a relative of an affected person compared to the risk in the population as a whole. More precisely, for a given relationship the RR for disease B in the relatives of probands with disease A is defined as



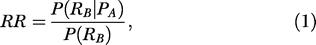



where *P_A_* denotes the event that the proband is affected with disease A, and *R_B_* denotes the event that the relative is affected with disease B. Note that disease A and disease B can be the same in this definition which applies when estimating RR at individual cancer sites. Using Bayes' rule it can be shown that for symmetric relationships, RR is the same if the roles of A and B are switched, i.e., the RR for disease A in the relatives of probands with disease B is the same as the described above. In this study we always chose the less common phenotype as the proband when estimating RR.

A basic underlying assumption in our estimation of RR is that of conditional independence of ascertainment, or censoring, (*O_RB_* and *O_PA_* are the events that the relative and proband are observed with diseases A and B, respectively):


*P*(*O_RB_*, *O_PA_* | *P_A_*, *R_B_*) = *P*(*O_RB_* | *R_B_*) *P*(*O_PA_* | *P_A_*).

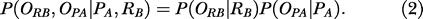



Some form of this assumption is used by most methods estimating RR [31].

Obtaining valid estimates of the RR is not always straightforward, since the method of ascertainment of affected cases critically affects the estimation, and inappropriate estimators can lead to bias or inflated estimates [32]. The use of a nationwide registry of patients covering close to five decades decreases much of the potential sampling bias. However, the ascertainment of the ICR depends on the year of birth of individuals. This dependence needs to be addressed when estimating the RR.

The approach chosen here is to estimate the RR for a number of subpopulations, where prevalence is reasonably constant, and combine them into a single estimate of RR for the full population. Let *r* be the number of relatives of probands, counting multiple times individuals who are relatives of multiple probands [33], let *a* be the number of relatives of probands that are affected (again possibly counting the same individual more than once), let *n* be the size of the population, and finally let *x* be the number of affected individuals in the population. If *P*(*R_B_*) and *P*(*R_B_ | P_A_*) can reasonably be assumed to be constant in the population, then *x/n* and *a/r,* respectively, are estimates of these probabilities. Given these estimates, RR is consistently estimated by



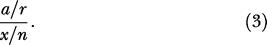



Assuming the population can be split into *N* subpopulations, such that within each subpopulation *P*(*R_B_*) and *P*(*R_B_ | P_A_*) can be assumed to be constant, although they may vary between subpopulations, and assuming furthermore that RR is the same in all the subpopulations, then the RR is consistently estimated by a convex combination of the estimates for the subpopulations. We selected weights for the combination such that the efficiency of the estimator was at maximum for RR equal to one. Making the simplifying assumption that the relatives are independent (while this assumption is not entirely correct, it affects only efficiency, not validity), the optimal weight for group *j* is



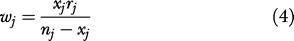



(this is the inverse of the variance of the estimate for RR in subpopulation *j*), where *a, r, x,* and *n* are defined as above, restricted to the subpopulation *j*. Note that probands are not restricted to the subpopulation. Given these weights, our estimate of RR is



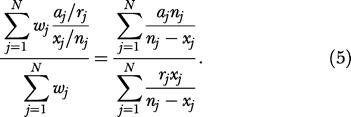



In this study, the most relevant variations in *P*(*R_B_*) and *P*(*R_B_ | P_A_*) stem from time-dependent censoring of affected status and sex-specific differences. Hence, we have stratified the population so that *j* runs over groups of people of the same sex and born in the same 5-y periods. For a fixed year-of-birth stratum, there is censoring of affected status (missing data) based on year of onset because of the fact that records cover only the period 1955–2002. Our approach is designed to address this type of missing data. As an example of the stratification, the breast cancer patients in our analysis were born in the years 1865 to 1970 (5-y strata), yielding 35 subpopulations, 22 for female patients, but only 13 for male, as this cancer is rare for males.

To assess the significance of the RR obtained for a given group of patients, we compared their observed values with the RR computed for up to 100,000 independently drawn and matched groups of control individuals. Each patient was matched to a single control individual in each control group. The control individuals were drawn at random from the genealogic database with the conditions that they had the same year of birth, the same sex, and the same number of ancestors recorded in the database at five generations back as the matched patients. Empirical *p* values can be calculated using the control groups; thus, a *p* value of 0.05 for the RR would indicate that 5% of the matched control groups had values as large as or larger than that for the patient's relatives or mates. The number of control groups required to obtain a fixed accuracy of the empirical *p* values is inversely proportional to the *p* value. We therefore selected the number of control groups generated adaptively up to a maximum of 100,000. When none of the values computed for the maximum number of control groups were larger than the observed value for the patient's relatives and mates, we report the *p* value as being less than 0.00001. Using a variance-stabilizing square-root transform, an approximate confidence interval may be constructed based on the distribution of RR for control groups [33].

As another test for significance of RR between cancer sites, we used combined estimators for risk in relatives of degree 1 and 2 together, degrees 3, 4, and 5 together, and degrees 1 through 5 together. If *RR_d_* is the RR for relatives of degree *d,* then *RR_d_* – 1 is known to decrease proportional to 2^-d^ as *d* increases for a monogenetic single variant or additive disease models, and faster for more complex disease models [34]. With the estimate of *RR_d_* denoted by 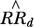, we then chose a test statistic of the form



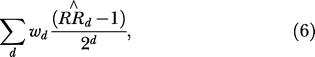



with *d* summed over the relevant degrees. For *RR_d_* close to one, the variance of the estimate 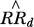 is inversely proportional to the number of relatives of degree *d* for the proband. Based on the Icelandic genealogy for the cancers being studied here, the number of relatives is proportional to γ*^d^,* where the value of γ quantifies how the number of relatives grows with each degree of relatedness to the proband. This factor γ varies only slightly between cancers and is on average 2.46. Minimizing the variance of the test statistic in equation 6 with respect to the weights yields the statistic



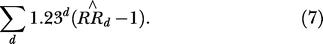



As above, the choice of weights and the form of the statistic affects only power, not validity. To assess significance, the observed value of the statistic was compared to its value for multiple matched control groups as described above.

Although our evaluations of familial clustering, for both close and distant relatives, are based on RR, an alternative approach based on comparing KCs among patients and among controls exists [12,24,25]. The two approaches are closely related, and our choice was made in part because relative risk is a less technical concept and its application to genetic counseling more direct. Also, the relationship between relative risk and the power to map disease genes by linkage analysis has been thoroughly investigated [34,35].

## Results

We have studied the familial clustering of cancer by estimating RR for first- and up to fifth-degree relatives both within and between all cancer sites. Here we present results for 27 sites that contain 200 or more cancer cases each, based on International Classification of Disease version 10 codes. These 27 sites represent 89% of all cancer cases in the ICR.

### Risk Estimations for Cancer at Same Site

A significantly increased RR to first-degree relatives of patients with cancer was seen for 22 of the 27 cancer sites (Table 1). Among the statistically significant RRs, the highest estimates were for lymphoid leukemia, Hodgkin's disease, and cancer of the thyroid, meninges, lip, testis, and larynx (RR above three). These cancers, except for thyroid cancer, were among the least prevalent sites (200–400 cases), as reflected in the large standard deviation of the RR estimates (Table 1). First-degree relatives of individuals with breast, lung, kidney, pancreatic, ovarian, and esophageal cancer and multiple myeloma, had between 2- and 3-fold increased risk of developing the same cancer.

The medians of the estimated RR values for the 27 sites in first- to fifth-degree relatives were 2.00, 1.32, 1.21, 1.10, and 1.04, respectively.

Combined *p*-values incorporating the increased risk for first- to fifth-degree relatives identified 21 sites being significant at a nominal level of 0.05. Sixteen of those sites remained significant after Bonferroni adjustment for the 27 individual tests (*p* value < 0.00185) (Table 1). To discriminate between familial clustering in close and distant relatives, combined *p* values were also calculated for first- and second-degree relatives on one hand and for third- to fifth-degree relatives on the other hand (Table 1). Fourteen sites were nominally significant for the distant relationships (third- to fifth-degree relatives) of which eight were significant after Bonferroni adjustment. These eight sites were all within the group of 16 sites demonstrating significant familial clustering in all relationships.

The RR for developing cancer at the same site was also estimated for mates of cancer patients at 22 out of the 27 individual sites. The remaining five sites are sex-specific and calculations thus not applicable. For seven rare cancer sites, affected mates were not observed, corresponding to a RR of zero. Only lung, stomach, and colon cancer were characterized by significantly increased RR values in mates (Table 1).

### Risk Estimations between Cancer Sites

**Figure 1:**
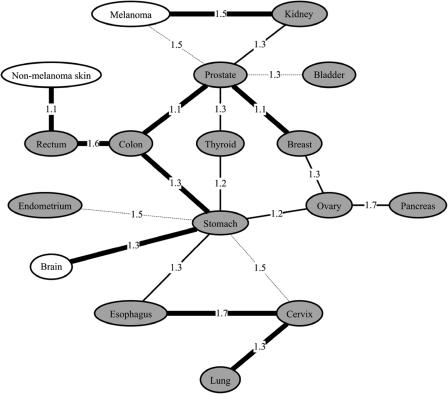
A Schematic Representation of Cancer Pairs Demonstrating Significant Familial Aggregation Cancer pairs that demonstrate significant familial co-clustering (first- to fifth-degree relatives) at the 0.05 level after adjustment for multiple testing (nominal *p* value < 1 × 10^-4^) are joined by lines. The thickness of the lines joining the pairs are based on nominal *p* values corresponding to the significance of the familiality in distant relatives (third to fifth degree): bold, *p* ≤ 0.001; solid, *p* ≤ 0.01; and dashed, *p* ≤ 0.05. The number on the lines joining each pair indicates the cross-cancer RR in first-degree relatives. Shaded ovals correspond to individual cancer sites that were significant for the combined group of first- to fifth-degree relatives at the 0.05 level after Bonferroni adjustment (see Table 1).

We calculated RR between all cancer sites for first- and up to fifth-degree relatives and mates (results for the 27 largest sites are shown in Table S1). As done for the individual cancer sites, *p* values were calculated for all (first- to fifth-degree), close (first- and second-degree), and distant (third- to fifth-degree) relationships. Figure 1 shows a diagram representing 20 pairs of cancer sites that associate with a combined *p* value, significant at a level of 1 × 10^-4^, for first- to fifth-degree relationships. This level was significant at the 0.05 level after Bonferroni adjustment for the 351 tests (number of unique pairs of cancers). The strength of the distant familiality (i.e., the *p* value for third- to fifth-degree relatives) between these pairs of cancers is represented by the thickness of the lines joining sites in Figure 1.

In total, 17 cancer sites were involved in 20 significant pairs of sites (Figure 1). Stomach and prostate cancer were involved in most pairs, seven and six pairs, respectively, followed by colon, ovarian, and cervical cancer, each involved in three pairs. The estimated RRs for the 20 pairs are between 1.1 and 1.7 for first-degree relatives and between 1.1 and 1.5 for second-degree relatives (Figure 1; Table S1). The highest RRs in first-degree relatives between cancer sites were seen for esophagus–cervix, with a RR of 1.74, pancreas–ovary, with a RR of 1.66, and colon–rectum, with a RR of 1.64.

All of the 20 pairs shown in Figure 1 were nominally significant (*p* value < 0.05) for distant relationships, of which nine were significant at the 0.001 level. In the latter group, prostate, rectum, stomach, and cervical cancers each appeared in two pairs, and colon cancer in three.

## Discussion

In this study we have comprehensively analyzed familial aggregation of cancer cases in a whole nation, both within and between pairs of cancer sites. The completeness of our genealogy database allows us to accurately trace distant relationships, which we believe is unique to this study. Linking the ICR to our nationwide genealogy database thus has made it possible to uncover distant familial connections between cancer cases, and reach beyond shared environmental factors to identify individual and combined cancer sites with the strongest genetic influences. Furthermore, even though the genetic effect decreases with more distant relationships, the sample sizes used to estimate familiality are dramatically larger for the distant relationships than for the closer ones. This compensates to some extent for the lower effect and adds considerable statistical power to the study.

In this paper we restrict the presentation and discussion to the most significant findings. However, we provide results for all pairs of 27 cancer sites in Table S1, as a resource for other researchers interested in the familiality of specific cancers.

The largest population-based studies reported to date, evaluating familial clustering within the same cancer site, are from Utah and Sweden [8,9,10]. These studies report RR values for first-degree relatives [36] that are comparable to those presented here for first-degree relatives. For example, the median RRs for the occurrence of the same cancer in first-degree relatives were 2.15, 1.86, and 2.00 for the Utah, the Sweden, and our study, respectively. Also, RR values in first-degree relatives ranged between 1.5 and 3.0 for the majority of sites, i.e., 69%, 82%, and 60%, in Utah, Sweden, and this study, respectively.

As seen in Utah and Sweden, high RR values were found in this study for multiple myeloma, lymphoid leukemia, and thyroid, testicular, and laryngeal cancer. The RR for thyroid cancer in first-degree relatives was much higher in Utah and Sweden (8.48 and 9.51) than in Iceland (3.02). One possible explanation of the lower RR may be the high incidence of thyroid cancer in Iceland, due to an excess of the papillary subtype [18,37], which is not a part of the multiple endocrine neoplasia syndromes.

The cancer sites showing the highest RR for first-degree relatives tend to be among the rarer sites. There are two potential reasons why rare tumors tend to show higher RRs than common cancers. Being common, the baseline frequency is not low and that creates a bound on how large the RR can be. Also, common cancers are expected to be genetically complex, whereas it is more likely for a rare tumor to be closer to a Mendelian trait, caused by rare alleles with high penetrances.

Most individual cancer sites, or 16 out of the 27 studied here, showed familiality as evidenced by significant *p* values (after adjustment for multiple testing) for the combined group of first- to fifth-degree relatives. Furthermore, eight of these 16 sites remained significant even after exclusion of the first- and second-degree relatives (after adjustment for multiple testing). The majority of the 16 significant cancer sites are among the sites of the most prevalent cancers, indicating that we may lack power to detect extended familiality for the less prevalent cancer sites. Indeed the median number of cases per cancer site was 943 for the 16 significant sites compared to 342 for the non-significant sites. Nevertheless, significant familial clustering (first- to fifth-degree relatives) is seen for some of the less prevalent sites, i.e., lymphoid leukemia and esophagus and meningeal cancer.

The largest cancer twin study reported to date [23] documented significant heritability of prostate (42%), colorectal (35%), and breast cancer (27%) and provided suggestive evidence for limited heritability of leukemia and stomach, lung, pancreas, ovarian, and bladder cancer. All of these cancer sites showed significant familial clustering in our study. However, when the analysis was restricted to distant relatives, lymphoid leukemia, pancreatic, and ovarian cancer were no longer significant. Although close to 45,000 pairs of twins were included in the study (of which 10,803 had been diagnosed with cancer), the study clearly lacked statistical power to detect the effects of heritable factors for the less prevalent cancer sites.

A significantly increased risk of the same cancer was seen in mates only for individuals diagnosed with stomach, lung, or colon cancer. These results are in accordance with previous reports, including Swedish population-based studies, except for colon cancer [38,39,40,41]. Environmental factors in adult life (including lifestyle and infections) or nonrandom mating could explain the higher risk of these cancer types in mates. The RR was not significant or not observed in mates for other sites.

We also assessed the significance of familial clustering between cancer sites by calculating combined *p* values corresponding to the increased risk for first- to fifth-degree relationships. With this method, we detected 17 cancers that linked into 20 pairs of sites that were significant after adjustment for multiple testing. Stomach and prostate cancer appeared more frequently in the pairs than other cancer types, followed by colon, ovarian, and cervical cancer. We emphasize again, as with the same-cancer calculations, that we might lack power to connect rare cancers to other cancer sites. This possibility is highlighted by the fact that the 17 cancers in the significant pairs are the most prevalent cancer sites in Iceland.

Some connections seen here between cancer sites may be partly explained by known high-risk genes involved in heritable syndromes. Thus, mutations in genes associated with hereditary nonpolyposis colorectal cancers could explain a part of the risk shared between stomach, colon, rectal, and endometrial cancer, and possibly brain and ovarian cancer [42,43]. In a similar manner, mutations in *BRCA1* and *BRCA2* may explain in part the cluster seen between prostate, breast, ovarian, and possibly pancreatic cancer [20,44,45,46]. Other known but even rarer cancer syndromes are likely to explain only a handful of cases.

Undiscovered genetic factors could contribute to some connections seen here to a much greater extent than the known susceptibility factors. Although these could include unknown high-risk susceptibility genes, they are more likely multiple genetic variants, each conferring small to moderate risk.

Familial clusters were identified between cancer sites, both in close and distant relatives, that do not correspond to known cancer syndromes. These include lung, esophageal, cervical, and stomach cancer, which, interestingly, have been associated with environmental rather than genetic factors. One explanation for this excess familiality between these cancer sites is an interaction of genetic susceptibility factors with environmental carcinogens (e.g., tobacco and diet) or infectious agents. Thus, the same environmental factor could interact with the same genetic susceptibility factor or factors to induce different cancers (i.e., smoking in lung and cervical cancer). Alternatively, different environmental factors could interact with the same genetic susceptibility factor or factors to increase the risk for different cancers (i.e., smoking in lung cancer and human papilloma virus in cervical cancer).

Hormone-related cancers form another risk cluster. Thus, shared genetic susceptibility factors could directly influence the hormonal metabolism to induce breast, prostate, thyroid, or ovarian cancer in carriers. Alternatively, shared genetic factors could interact with dietary factors to induce aggregation of cancers at these sites in related individuals. A significantly increased risk of breast, prostate, cervical, and non-melanoma skin cancer was recently reported in first-degree relatives of early-onset breast cancer patients from Sweden that tested negative for *BRCA1* and *BRCA2* mutations [47]. Our data support the notion that unknown susceptibility variants that increase the risk of breast and prostate cancer and melanoma remain to be characterized.

Two more groups of cancers with shared risk were identified that each include sites that share the same developmental progenitors: the prostate, kidney, and bladder are sites derived from the nephrogenic ridge while colon, rectum, and stomach are derived from the primitive gut tube. Therefore, the sites in each group may share risk alleles that regulate embryonic development, which can later play a role in oncogenesis.

Interestingly, three cancer sites/types, non-melanoma skin, brain, and melanoma, that do not have significant same-cancer familial clustering demonstrate significant cross-cancer familial clustering with more prevalent cancer sites, i.e., rectum, stomach, and kidney cancers, respectively.

Previous reports systematically evaluating the significance of co-clustering of cancer pairs in families have utilized the Utah Population Database. In these studies lip and prostate cancers appear to associate most frequently with other cancer sites. The same is true for prostate cancer in our study, whereas lip cancer does not significantly associate with any other cancer sites. This can at least in part be explained by the difference in age-standardized incidence rates for lip cancer in Iceland and Utah (Iceland 1.1 and Utah 2.4) [48]. In contrast, stomach cancer associates with seven other cancer sites out of the 20 significant pairs in our study, but only three other sites in the Utah study. Of the 20 cancer pairs that significantly associate in our study, eight concur with the Utah studies.

Because the increased cross-site RR extends beyond the nuclear family, shared genetic factors may contribute to the risk of more than one cancer type. This suggests that cancer could be considered a broad phenotype with shared genetic factors across cancer sites. Therefore cancer should in certain cases be studied in a broader context than previously done. Combining multiple cancers that show increased cross-site RR may serve to increase the power of linkage and case-control studies. Our results also have implications for genetic counseling and imply that the focus of attention should broaden to the history of multiple cancer types in relatives within and outside the nuclear family. These results also suggest the utility of comparing expression profiles and in vitro biological processes across the cancers that we have identified as sharing genetic risk. The isolation of cancer predisposition genes with broad effects may define new rate-limiting pathways that can be used to search for drug targets for a more focused treatment with fewer side effects but with utility across multiple cancers.

## Supporting Information

Table S1 Cross-Site RR Estimates for Relatives and Mates of Patients Diagnosed with Cancers at 27 Sites with 200 Cases or More (1.9 MB DOC).

### Accession Numbers

The LocusLink (http://www.ncbi.nlm.nih.gov/projects/LocusLink/) accession numbers for the genes discussed in this paper are *BRCA1*(LocusLink ID 672) and *BRCA2* (LocusLink ID 675).

**Patient Summary**

**Background**

Although a few cancers have a fairly simple genetic cause, most, especially the most common cancers, do not, and what makes one person rather than another develop cancer is not clear. One way of trying to work out how much genes rather than environment contribute to disease is to study large populations. One such population is the Icelandic nation: not only is detailed health information about individuals available, including information on cancer, but also very good genealogical information and a substantial amount of genetic data.

**What Did the Study Find?**

Researchers examined all cancer records dating back to 1955 and then analyzed the chances of relatives and mates of these patients having cancer. They found that some cancers, especially rare ones, had a higher than baseline chance of occurring in relatives, but so did many common cancers, and for some cancers, the higher chances extended to quite distant relatives. In addition, the risk sometimes involved different cancer types.

**What Does the Study Mean for Patients?**

Even for the highest risk cancers, the absolute increased risk for relatives remains very small. In addition, despite the large numbers of patients studied, the numbers of cancer cases are still not large enough to be completely certain of the results, apart from very common cancers, which had the lowest chance of occurring in relatives. So these results will not help doctors much at the present time in telling an individual patient what their risk is of getting cancer if a relative has it—but they will be useful for other researchers in knowing how to plan future studies to look at the underlying causes of cancer.

**Where Can I Get More Information?**

Icelandic Cancer Society: http://www.krabb.is/cancer/

The United States National Cancer Institute's Cancer Information Service: http://cis.nci.nih.gov/

CancerHelp UK, a free information service about cancer and cancer care: http://www.cancerhelp.org.uk/

deCODE Genetics:http://www.decode.com

## Abbreviations

ICR: Icelandic Cancer Registry
KC: kinship coefficient
RR: risk ratio
