# Supplementary material for: Cancer as a Complex Phenotype: Pattern of Cancer Distribution within and beyond the Nuclear Family
Source: PLoS Med. 2004 Dec 28;1(3):e65. doi: 10.1371/journal.pmed.0010065 (PMC539051; doi:10.1371/journal.pmed.0010065)
Supplement: Table S1 — (1.9 MB DOC). [file pmed.0010065.st001.doc]

| **Table 2** RR estimates for relatives and mates of patients diagnosed with breast cancer for sites with 200 or more cases^a^ | | | | | | | | | | | |
| --- | --- | --- | --- | --- | --- | --- | --- | --- | --- | --- | --- |
| Cancer sites | # of aff. | RR [90% CI] | | | | | |  | combined p-value for relatives | | |
|  |  | 1º relative | 2º relatives | 3º relatives | 4º relatives | 5º relatives | Mates |  | 1º-5º | 1º-2º | 3º-5º |
| Breast | 3812 | **2.02 [1.88,2.15]** | **1.36 [1.27,1.43]** | **1.21 [1.15,1.25]** | **1.13 [1.08,1.16]** | **1.05 [1.01,1.06]** | 2.02 |  | <0.00001 | <0.00001 | <0.00001 |
| Prostate | 3380 | **1.10 [1.02,1.17]** | **1.08 [1.01,1.12]** | **1.07 [1.02,1.10]** | 1.04 [1.00,1.05] | **1.06 [1.02,1.07]** | 1.09 |  | 0.00001 | 0.00003 | 0.0002 |
| Lung | 2904 | 1.10 [1.00,1.16] | 1.04 [0.96,1.09] | 1.05 [1.00,1.08] | 0.99 [0.95,1.01] | 1.00 [0.96,1.01] | 1.16 |  | 0.17097 | 0.48014 | 0.66022 |
| Stomach | 2890 | 1.08 [0.99,1.16] | 1.05 [0.98,1.09] | 1.00 [0.95,1.03] | 1.00 [0.96,1.02] | **1.05 [1.01,1.06]** | 1.01 |  | 0.06579 | 0.17786 | 0.32053 |
| Colon | 2224 | **1.13 [1.02,1.21]** | 1.06 [0.98,1.11] | 1.03 [0.98,1.07] | 1.04 [0.99,1.06] | **1.07 [1.03,1.08]** | 1.06 |  | 0.00022 | 0.00242 | 0.00269 |
| Bladder | 1384 | 1.07 [0.95,1.18] | 1.10 [1.00,1.18] | 1.05 [0.99,1.11] | 1.00 [0.94,1.02] | 1.02 [0.98,1.04] | 1.28 |  | 0.050 | 0.082 | 0.300 |
| Kidney | 1227 | 1.06 [0.94,1.20] | **1.12 [1.02,1.20]** | 1.03 [0.96,1.08] | 1.01 [0.96,1.04] | 1.04 [1.00,1.06] | 0.93 |  | 0.02958 | 0.0377 | 0.21246 |
| Thyroid | 957 | **1.21 [1.06,1.37]** | 1.03 [0.92,1.13] | 1.01 [0.93,1.06] | 1.04 [0.98,1.07] | 1.01 [0.96,1.03] | 1.17 |  | 0.05065 | 0.35495 | 0.3868 |
| Pancreas | 930 | 1.11 [0.96,1.24] | **1.12 [1.01,1.22]** | 1.04 [0.96,1.10] | 1.03 [0.97,1.06] | 1.00 [0.96,1.02] | 1.05 |  | 0.04072 | 0.09043 | 0.36245 |
| Ovary | 906 | **1.34 [1.17,1.50]** | 1.13 [1.00,1.23] | **1.10 [1.01,1.16]** | 1.06 [0.99,1.10] | 1.03 [0.99,1.05] | 0.00 |  | 0.00001 | 0.00187 | 0.00955 |
| Non-melanoma skin | 781 | 0.94 [0.79,1.08] | 1.05 [0.93,1.15] | 1.08 [0.99,1.14] | 0.99 [0.93,1.02] | 0.99 [0.95,1.01] | 1.34 |  | 0.589 | 0.386 | 0.494 |
| Rectum | 767 | 1.11 [0.95,1.27] | **1.20 [1.09,1.31]** | 1.04 [0.96,1.11] | 1.02 [0.96,1.06] | **1.09 [1.04,1.12]** | 1.13 |  | 0.00046 | 0.00055 | 0.01638 |
| Endometrium | 753 | 1.05 [0.90,1.18] | 1.14 [1.00,1.24] | 1.01 [0.93,1.07] | 0.95 [0.88,0.98] | 1.05 [1.00,1.08] | 2.68 |  | 0.230 | 0.270 | 0.710 |
| Cervix uteri | 724 | 1.03 [0.88,1.21] | 0.97 [0.84,1.07] | 1.05 [0.96,1.11] | 0.97 [0.90,1.01] | 0.98 [0.93,1.00] | 0.00 |  | 0.814 | 0.904 | 0.865 |
| Brain | 663 | 1.00 [0.83,1.17] | 1.03 [0.91,1.16] | 1.07 [0.98,1.14] | 1.01 [0.94,1.06] | 1.00 [0.95,1.02] | 1.64 |  | 0.388 | 0.342 | 0.392 |
| Melanoma of skin | 618 | 0.80 [0.65,0.98] | **1.22 [1.09,1.34]** | **1.11 [1.01,1.19]** | 1.02 [0.95,1.07] | **1.07 [1.02,1.10]** | 1.42 |  | 0.0523 | 0.0004 | 0.0095 |
| Esophagus | 535 | 1.03 [0.84,1.21] | 1.05 [0.92,1.18] | 0.99 [0.90,1.07] | 0.92 [0.86,0.96] | 1.04 [0.99,1.07] | 1.49 |  | 0.703 | 0.739 | 0.903 |
| Diffuse NHL^b^ | 422 | 1.05 [0.84,1.27] | 0.87 [0.72,1.00] | 0.91 [0.80,1.00] | 1.07 [0.99,1.13] | 1.06 [1.00,1.09] | 1.67 |  | 0.690 | 0.798 | 0.393 |
| Multiple myeloma | 391 | 1.10 [0.86,1.30] | **1.35 [1.18,1.53]** | 1.02 [0.91,1.11] | 0.98 [0.89,1.04] | 1.03 [0.97,1.07] | 0.73 |  | 0.02093 | 0.02072 | 0.56336 |
| Lymphoid leukaemia | 368 | 0.92 [0.69,1.17] | 1.08 [0.90,1.23] | 1.07 [0.96,1.17] | 1.08 [0.99,1.14] | 1.01 [0.95,1.05] | 0.58 |  | 0.228 | 0.081 | 0.093 |
| Myeloid leukaemia | 342 | **1.42 [1.14,1.67]** | 1.05 [0.88,1.20] | 0.98 [0.87,1.09] | 1.04 [0.94,1.10] | 1.01 [0.94,1.05] | 1.70 |  | 0.051 | 0.478 | 0.559 |
| Meninges | 291 | **1.34 [1.04,1.63]** | 1.14 [0.95,1.31] | 0.94 [0.82,1.04] | 1.02 [0.93,1.10] | 1.03 [0.97,1.08] | 1.20 |  | 0.044 | 0.286 | 0.588 |
| Liver | 257 | 0.98 [0.76,1.26] | 1.15 [0.96,1.34] | 1.05 [0.90,1.17] | 1.00 [0.91,1.08] | 1.07 [1.00,1.11] | 1.02 |  | 0.139 | 0.077 | 0.218 |
| Lip | 244 | 0.76 [0.54,0.99] | 0.86 [0.69,1.04] | 0.99 [0.85,1.12] | 0.89 [0.80,0.96] | 1.05 [0.98,1.11] | 1.52 |  | 0.986 | 0.957 | 0.831 |
| Hodgkin's disease | 239 | 1.04 [0.76,1.42] | 0.95 [0.75,1.13] | **1.16 [1.01,1.31]** | 1.02 [0.93,1.10] | 1.02 [0.95,1.08] | 1.23 |  | 0.235 | 0.229 | 0.076 |
| Testis | 222 | 0.85 [0.57,1.24] | 0.85 [0.68,1.06] | **1.23 [1.04,1.37]** | 0.95 [0.85,1.04] | 1.06 [0.98,1.11] | 1.95 |  | 0.583 | 0.363 | 0.088 |
| Larynx | 208 | 1.00 [0.72,1.34] | 0.82 [0.62,1.02] | **1.17 [1.01,1.31]** | 0.98 [0.88,1.06] | 1.08 [1.00,1.15] | 1.77 |  | 0.419 | 0.401 | 0.046 |
| ^a^ Shown are the estimated risk ratios (RR) with 90% confidence intervals (CI) for 1st to 5th degree (1º-5º) relatives and mates of the 27 cancer sites with ≥200 cases, in bold when the 90% CI does not include 1.00, which corresponds to one-sided p-value <0.05. Also shown are combined p-values (one-sided) to evaluate the significance of the RR for all relatives (1^st^-5^th^ degree) and for close (1^st^ and 2^nd^ degree) and distant relatives (3^rd^-5^th^ degree). | | | | | | | | | | | |
| ^b^ NHL, non-Hodgkin's lymphoma. | | | | | | | | | | | |
| ^c^ na, not applicable (sex-specific cancers). | | | | | | | | | | | |
| ^d^ Number of mates or relatives with cancer/total number of mates or relatives for each cancer site | | | | | | | | | | | |

| **Table 3** RR estimates for relatives and mates of patients diagnosed with prostate cancer for sites with 200 or more cases^a^ | | | | | | | | | | | |
| --- | --- | --- | --- | --- | --- | --- | --- | --- | --- | --- | --- |
| Cancer sites | # of aff. | RR [90% CI] | | | | | |  | combined p-value for relatives | | |
|  |  | 1º relative | 2º relatives | 3º relatives | 4º relatives | 5º relatives | Mates |  | 1º-5º | 1º-2º | 3º-5º |
| Breast | 3812 | **1.10 [1.02,1.17]** | **1.08 [1.01,1.12]** | **1.07 [1.02,1.10]** | 1.04 [1.00,1.05] | **1.06 [1.02,1.07]** | 1.09 |  | 0.00001 | 0.00003 | 0.0002 |
| Prostate | 3380 | **1.89 [1.75,2.01]** | **1.36 [1.26,1.45]** | **1.19 [1.13,1.24]** | **1.10 [1.05,1.13]** | **1.10 [1.07,1.12]** | na^c^ |  | <0.00001 | <0.00001 | <0.00001 |
| Lung | 2904 | **1.10 [1.02,1.17]** | 0.99 [0.91,1.04] | 0.98 [0.93,1.01] | **1.05 [1.01,1.08]** | 1.02 [0.98,1.03] | 1.30 |  | 0.169 | 0.491 | 0.333 |
| Stomach | 2890 | 1.03 [0.94,1.10] | 1.00 [0.93,1.04] | **1.07 [1.01,1.10]** | 1.03 [0.99,1.05] | 1.02 [0.99,1.04] | 1.41 |  | 0.06394 | 0.06349 | 0.01424 |
| Colon | 2224 | **1.15 [1.05,1.23]** | 1.05 [0.97,1.11] | **1.08 [1.02,1.12]** | 1.04 [1.00,1.07] | **1.04 [1.01,1.06]** | 1.44 |  | 0.00002 | 0.00105 | 0.00059 |
| Bladder | 1384 | **1.34 [1.20,1.46]** | **1.12 [1.02,1.21]** | 1.04 [0.97,1.09] | **1.07 [1.02,1.10]** | 1.01 [0.97,1.03] | 1.10 |  | <0.00001 | 0.0062 | 0.04423 |
| Kidney | 1227 | **1.31 [1.16,1.43]** | **1.18 [1.07,1.28]** | 1.07 [1.00,1.12] | 1.05 [0.99,1.08] | 1.04 [1.00,1.06] | 1.75 |  | 0.00001 | 0.00014 | 0.0086 |
| Thyroid | 957 | **1.30 [1.14,1.45]** | **1.19 [1.07,1.30]** | 1.06 [0.98,1.12] | **1.07 [1.01,1.11]** | 1.04 [1.00,1.07] | 1.42 |  | <0.00001 | 0.00018 | 0.0061 |
| Pancreas | 930 | 1.13 [0.98,1.25] | 1.12 [1.00,1.22] | 1.04 [0.96,1.10] | 1.06 [1.00,1.10] | **1.06 [1.02,1.08]** | 1.03 |  | 0.0008 | 0.0021 | 0.0095 |
| Ovary | 906 | **1.20 [1.05,1.35]** | 1.00 [0.88,1.10] | 1.05 [0.98,1.12] | 1.04 [0.99,1.08] | 1.05 [1.00,1.08] | 1.09 |  | 0.013 | 0.083 | 0.021 |
| Non-melanoma skin | 781 | 1.06 [0.90,1.21] | 1.02 [0.89,1.12] | 0.99 [0.91,1.06] | 0.98 [0.91,1.02] | 1.02 [0.98,1.05] | 1.35 |  | 0.566 | 0.680 | 0.715 |
| Rectum | 767 | 0.99 [0.84,1.15] | 1.04 [0.91,1.15] | 0.90 [0.82,0.97] | 0.95 [0.89,0.99] | 1.01 [0.97,1.05] | 1.11 |  | 0.944 | 0.961 | 0.999 |
| Endometrium | 753 | 1.06 [0.91,1.23] | **1.18 [1.03,1.29]** | 1.07 [0.98,1.14] | 0.99 [0.92,1.03] | 1.00 [0.95,1.03] | 1.15 |  | 0.063 | 0.078 | 0.467 |
| Cervix uteri | 724 | 1.03 [0.86,1.21] | 1.10 [0.96,1.21] | 0.98 [0.89,1.05] | 1.04 [0.97,1.09] | 1.03 [0.98,1.06] | 0.93 |  | 0.191 | 0.172 | 0.336 |
| Brain | 663 | 1.08 [0.91,1.28] | 1.09 [0.96,1.22] | **1.15 [1.05,1.23]** | 0.94 [0.88,0.99] | 1.03 [0.98,1.06] | 1.08 |  | 0.074 | 0.090 | 0.175 |
| Melanoma of skin | 618 | **1.49 [1.25,1.74]** | **1.14 [1.01,1.28]** | **1.15 [1.06,1.24]** | 1.06 [0.99,1.11] | 1.01 [0.96,1.04] | 1.16 |  | <0.00001 | 0.00286 | 0.0112 |
| Esophagus | 535 | **1.20 [1.02,1.40]** | 0.89 [0.76,1.02] | 1.04 [0.93,1.13] | 1.02 [0.95,1.08] | 1.05 [1.00,1.09] | 1.68 |  | 0.159 | 0.453 | 0.114 |
| Diffuse NHL^b^ | 422 | 1.15 [0.95,1.40] | 1.10 [0.92,1.29] | 1.06 [0.94,1.14] | 1.05 [0.96,1.12] | 1.03 [0.98,1.07] | 0.66 |  | 0.024 | 0.054 | 0.109 |
| Multiple myeloma | 391 | **1.30 [1.09,1.53]** | 1.15 [0.99,1.33] | 1.09 [0.98,1.17] | 1.05 [0.96,1.12] | 1.04 [0.98,1.08] | 1.26 |  | 0.0014 | 0.016 | 0.042 |
| Lymphoid leukaemia | 368 | 1.02 [0.79,1.30] | 0.97 [0.82,1.15] | 1.02 [0.90,1.11] | 0.99 [0.91,1.06] | 1.02 [0.95,1.06] | 1.83 |  | 0.585 | 0.631 | 0.558 |
| Myeloid leukaemia | 342 | **1.34 [1.04,1.59]** | **1.22 [1.03,1.43]** | 1.11 [1.00,1.22] | 0.98 [0.90,1.05] | 1.03 [0.97,1.08] | 1.06 |  | 0.0018 | 0.0259 | 0.1965 |
| Meninges | 291 | 1.18 [0.91,1.46] | 1.15 [0.96,1.36] | 1.08 [0.95,1.20] | **1.11 [1.02,1.21]** | 1.08 [1.00,1.13] | 1.09 |  | 0.0027 | 0.0046 | 0.0077 |
| Liver | 257 | 1.02 [0.77,1.29] | 0.88 [0.70,1.09] | 1.05 [0.92,1.17] | 1.00 [0.89,1.09] | 1.06 [0.99,1.11] | 1.87 |  | 0.529 | 0.545 | 0.239 |
| Lip | 244 | 1.11 [0.85,1.43] | 0.94 [0.74,1.18] | 1.09 [0.96,1.23] | **1.17 [1.05,1.26]** | 1.00 [0.93,1.05] | 0.00 |  | 0.091 | 0.096 | 0.021 |
| Hodgkin's disease | 239 | 0.96 [0.65,1.30] | 1.05 [0.88,1.27] | 1.09 [0.94,1.23] | 0.99 [0.90,1.09] | 1.02 [0.94,1.07] | 1.25 |  | 0.356 | 0.238 | 0.290 |
| Testis | 222 | **1.59 [1.16,2.28]** | **1.23 [1.01,1.46]** | 0.86 [0.71,1.01] | **1.17 [1.06,1.28]** | 1.04 [0.96,1.10] | na^c^ |  | 0.0031 | 0.0621 | 0.2653 |
| Larynx | 208 | 0.94 [0.72,1.22] | 0.91 [0.69,1.14] | 0.85 [0.71,0.99] | 0.94 [0.83,1.04] | 1.03 [0.96,1.10] | 0.67 |  | 0.938 | 0.944 | 0.936 |
| ^a^ Shown are the estimated risk ratios (RR) with 90% confidence intervals (CI) for 1st to 5th degree (1º-5º) relatives and mates of the 27 cancer sites with ≥200 cases, in bold when the 90% CI does not include 1.00, which corresponds to one-sided p-value <0.05. Also shown are combined p-values (one-sided) to evaluate the significance of the RR for all relatives (1^st^-5^th^ degree) and for close (1^st^ and 2^nd^ degree) and distant relatives (3^rd^-5^th^ degree). | | | | | | | | | | | |
| ^b^ NHL, non-Hodgkin's lymphoma. | | | | | | | | | | | |
| ^c^ na, not applicable (sex-specific cancers). | | | | | | | | | | | |
| ^d^ Number of mates or relatives with cancer/total number of mates or relatives for each cancer site | | | | | | | | | | | |

| **Table 4** RR estimates for relatives and mates of patients diagnosed with lung cancer for sites with 200 or more cases^a^ | | | | | | | | | | | |
| --- | --- | --- | --- | --- | --- | --- | --- | --- | --- | --- | --- |
| Cancer sites | # of aff. | RR [90% CI] | | | | | |  | combined p-value for relatives | | |
|  |  | 1º relative | 2º relatives | 3º relatives | 4º relatives | 5º relatives | Mates |  | 1º-5º | 1º-2º | 3º-5º |
| Breast | 3812 | 1.10 [1.00,1.16] | 1.04 [0.96,1.09] | 1.05 [1.00,1.08] | 0.99 [0.95,1.01] | 1.00 [0.96,1.01] | 1.16 |  | 0.17097 | 0.48014 | 0.66022 |
| Prostate | 3380 | **1.10 [1.02,1.17]** | 0.99 [0.91,1.04] | 0.98 [0.93,1.01] | **1.05 [1.01,1.08]** | 1.02 [0.98,1.03] | 1.30 |  | 0.169 | 0.491 | 0.333 |
| Lung | 2904 | **2.00 [1.83,2.16]** | **1.39 [1.26,1.50]** | **1.10 [1.03,1.16]** | 1.02 [0.97,1.07] | **1.04 [1.01,1.08]** | 1.68 |  | <0.00001 | <0.00001 | 0.00135 |
| Stomach | 2890 | 1.04 [0.95,1.13] | **1.14 [1.07,1.20]** | 1.01 [0.97,1.06] | 1.01 [0.98,1.05] | 1.02 [0.99,1.05] | 1.41 |  | 0.00218 | 0.00189 | 0.11944 |
| Colon | 2224 | **1.17 [1.06,1.27]** | 1.03 [0.95,1.11] | **1.07 [1.02,1.12]** | 1.02 [0.98,1.06] | 1.01 [0.99,1.04] | 1.38 |  | 0.0014 | 0.0247 | 0.0152 |
| Bladder | 1384 | **1.27 [1.15,1.43]** | **1.12 [1.02,1.23]** | 1.05 [0.99,1.11] | 1.00 [0.95,1.05] | 1.00 [0.98,1.04] | 1.35 |  | 0.00033 | 0.03663 | 0.18905 |
| Kidney | 1227 | 1.11 [0.99,1.26] | 1.09 [0.98,1.20] | 1.00 [0.94,1.07] | 0.99 [0.93,1.03] | 0.95 [0.92,0.98] | 0.96 |  | 0.325 | 0.631 | 0.907 |
| Thyroid | 957 | 0.99 [0.84,1.18] | 0.96 [0.85,1.07] | 1.03 [0.96,1.13] | 1.02 [0.97,1.08] | 1.00 [0.96,1.04] | 1.17 |  | 0.406 | 0.365 | 0.183 |
| Pancreas | 930 | **1.20 [1.05,1.37]** | 1.09 [0.97,1.21] | 1.01 [0.94,1.08] | **1.08 [1.03,1.14]** | 1.02 [0.99,1.06] | 1.06 |  | 0.00148 | 0.01326 | 0.02842 |
| Ovary | 906 | **1.18 [1.01,1.35]** | 1.05 [0.93,1.18] | 1.04 [0.96,1.12] | 0.97 [0.92,1.03] | 0.99 [0.95,1.03] | 1.23 |  | 0.114 | 0.356 | 0.529 |
| Non-melanoma skin | 781 | 1.11 [0.95,1.27] | 1.11 [0.97,1.24] | 1.04 [0.96,1.13] | 1.04 [0.97,1.10] | 0.99 [0.96,1.04] | 0.61 |  | 0.034 | 0.049 | 0.160 |
| Rectum | 767 | 1.08 [0.93,1.25] | **1.23 [1.09,1.37]** | 0.95 [0.86,1.03] | 0.98 [0.91,1.03] | 0.98 [0.94,1.03] | 1.11 |  | 0.155 | 0.249 | 0.908 |
| Endometrium | 753 | 0.90 [0.75,1.06] | 1.03 [0.89,1.16] | 0.94 [0.86,1.02] | 1.02 [0.96,1.08] | 0.98 [0.94,1.03] | 1.13 |  | 0.821 | 0.639 | 0.776 |
| Cervix uteri | 724 | **1.31 [1.11,1.52]** | **1.32 [1.14,1.46]** | **1.17 [1.07,1.26]** | **1.10 [1.02,1.15]** | 1.04 [0.99,1.08] | 2.01 |  | <0.00001 | <0.00001 | 0.00008 |
| Brain | 663 | 1.13 [0.92,1.33] | 0.92 [0.78,1.05] | 0.99 [0.90,1.08] | **1.08 [1.01,1.15]** | 0.98 [0.95,1.03] | 1.17 |  | 0.327 | 0.553 | 0.218 |
| Melanoma of skin | 618 | 1.07 [0.87,1.31] | **1.29 [1.12,1.43]** | 1.00 [0.91,1.09] | 0.98 [0.92,1.05] | 1.01 [0.97,1.06] | 0.39 |  | 0.034 | 0.032 | 0.468 |
| Esophagus | 535 | **1.36 [1.13,1.60]** | 1.11 [0.96,1.29] | 1.00 [0.89,1.10] | 1.05 [0.99,1.14] | 1.01 [0.96,1.06] | 1.71 |  | 0.00351 | 0.08273 | 0.18707 |
| Diffuse NHL^b^ | 422 | 1.04 [0.83,1.29] | 0.99 [0.81,1.15] | 0.98 [0.88,1.09] | 1.04 [0.96,1.12] | 0.99 [0.94,1.04] | 1.26 |  | 0.480 | 0.561 | 0.477 |
| Multiple myeloma | 391 | 1.11 [0.87,1.36] | 1.14 [0.95,1.33] | 1.04 [0.93,1.16] | 1.01 [0.92,1.09] | 0.93 [0.88,0.98] | 1.19 |  | 0.231 | 0.351 | 0.667 |
| Lymphoid leukaemia | 368 | 0.63 [0.42,0.88] | 1.14 [0.95,1.33] | 1.09 [0.98,1.22] | 1.02 [0.94,1.10] | 0.98 [0.93,1.04] | 1.23 |  | 0.644 | 0.085 | 0.203 |
| Myeloid leukaemia | 342 | 1.03 [0.77,1.31] | 1.20 [0.98,1.42] | 1.07 [0.95,1.22] | 0.97 [0.89,1.06] | 0.98 [0.92,1.04] | 1.54 |  | 0.175 | 0.159 | 0.435 |
| Meninges | 291 | 1.16 [0.90,1.49] | 1.14 [0.95,1.38] | 1.01 [0.88,1.16] | 1.00 [0.91,1.11] | 0.99 [0.93,1.06] | 1.29 |  | 0.092 | 0.202 | 0.444 |
| Liver | 257 | **1.62 [1.29,2.02]** | 0.91 [0.73,1.17] | 1.02 [0.88,1.17] | 1.06 [0.96,1.16] | 1.00 [0.94,1.06] | 1.57 |  | 0.017 | 0.405 | 0.215 |
| Lip | 244 | 1.08 [0.78,1.40] | 1.03 [0.81,1.29] | **1.19 [1.04,1.36]** | 1.04 [0.94,1.15] | 0.89 [0.83,0.96] | 1.21 |  | 0.272 | 0.290 | 0.291 |
| Hodgkin's disease | 239 | 0.80 [0.52,1.14] | 1.00 [0.79,1.26] | 1.01 [0.85,1.17] | 1.06 [0.94,1.18] | 0.98 [0.91,1.05] | 1.31 |  | 0.666 | 0.449 | 0.428 |
| Testis | 222 | 1.18 [0.84,1.74] | 0.80 [0.61,1.03] | 0.94 [0.78,1.10] | **1.11 [1.01,1.24]** | **1.11 [1.03,1.20]** | 0.00 |  | 0.222 | 0.335 | 0.051 |
| Larynx | 208 | **2.03 [1.61,2.53]** | 1.11 [0.88,1.41] | 1.04 [0.88,1.19] | 1.00 [0.89,1.11] | 0.94 [0.88,1.02] | 1.73 |  | 0.00165 | 0.44915 | 0.6282 |
| ^a^ Shown are the estimated risk ratios (RR) with 90% confidence intervals (CI) for 1st to 5th degree (1º-5º) relatives and mates of the 27 cancer sites with ≥200 cases, in bold when the 90% CI does not include 1.00, which corresponds to one-sided p-value <0.05. Also shown are combined p-values (one-sided) to evaluate the significance of the RR for all relatives (1^st^-5^th^ degree) and for close (1^st^ and 2^nd^ degree) and distant relatives (3^rd^-5^th^ degree). | | | | | | | | | | | |
| ^b^ NHL, non-Hodgkin's lymphoma. | | | | | | | | | | | |
| ^c^ na, not applicable (sex-specific cancers). | | | | | | | | | | | |
| ^d^ Number of mates or relatives with cancer/total number of mates or relatives for each cancer site | | | | | | | | | | | |

| **Table 5** RR estimates for relatives and mates of patients diagnosed with stomach cancer for sites with 200 or more cases^a^ | | | | | | | | | | | |
| --- | --- | --- | --- | --- | --- | --- | --- | --- | --- | --- | --- |
| Cancer sites | # of aff. | RR [90% CI] | | | | | |  | combined p-value for relatives | | |
|  |  | 1º relative | 2º relatives | 3º relatives | 4º relatives | 5º relatives | Mates |  | 1º-5º | 1º-2º | 3º-5º |
| Breast | 3812 | 1.08 [0.99,1.16] | 1.05 [0.98,1.09] | 1.00 [0.95,1.03] | 1.00 [0.96,1.02] | **1.05 [1.01,1.06]** | 1.01 |  | 0.06579 | 0.17786 | 0.32053 |
| Prostate | 3380 | 1.03 [0.94,1.10] | 1.00 [0.93,1.04] | **1.07 [1.01,1.10]** | 1.03 [0.99,1.05] | 1.02 [0.99,1.04] | 1.41 |  | 0.06394 | 0.06349 | 0.01424 |
| Lung | 2904 | 1.04 [0.95,1.13] | **1.14 [1.07,1.20]** | 1.01 [0.97,1.06] | 1.01 [0.98,1.05] | 1.02 [0.99,1.05] | 1.41 |  | 0.00218 | 0.00189 | 0.11944 |
| Stomach | 2890 | **1.90 [1.74,2.05]** | **1.38 [1.25,1.48]** | **1.23 [1.16,1.29]** | **1.15 [1.09,1.19]** | **1.09 [1.04,1.11]** | 1.72 |  | <0.00001 | <0.00001 | <0.00001 |
| Colon | 2224 | **1.26 [1.14,1.36]** | 1.08 [0.99,1.15] | **1.10 [1.04,1.14]** | **1.06 [1.01,1.08]** | 1.04 [1.00,1.05] | 1.37 |  | <0.00001 | 0.00032 | 0.00049 |
| Bladder | 1384 | **1.20 [1.06,1.33]** | 1.08 [0.96,1.16] | 1.07 [0.99,1.12] | 1.05 [0.99,1.09] | 1.03 [0.99,1.05] | 1.31 |  | 0.00121 | 0.01464 | 0.02383 |
| Kidney | 1227 | **1.19 [1.03,1.32]** | 1.02 [0.90,1.11] | **1.13 [1.04,1.18]** | 1.07 [1.00,1.10] | 1.04 [1.00,1.07] | 1.08 |  | 0.00063 | 0.00461 | 0.00058 |
| Thyroid | 957 | 1.16 [0.99,1.33] | **1.25 [1.12,1.36]** | **1.15 [1.06,1.22]** | 1.06 [0.99,1.10] | 1.03 [0.98,1.05] | 1.66 |  | 0.00003 | 0.00003 | 0.00199 |
| Pancreas | 930 | **1.23 [1.07,1.39]** | 1.12 [0.98,1.23] | 1.07 [0.98,1.14] | 1.00 [0.93,1.04] | **1.07 [1.02,1.10]** | 1.58 |  | 0.0013 | 0.0205 | 0.0654 |
| Ovary | 906 | **1.23 [1.04,1.42]** | **1.30 [1.16,1.43]** | **1.10 [1.01,1.16]** | 1.05 [0.98,1.10] | **1.07 [1.02,1.10]** | 0.96 |  | <0.00001 | <0.00001 | 0.00267 |
| Non-melanoma skin | 781 | 1.14 [0.96,1.32] | 1.13 [0.97,1.25] | 1.03 [0.94,1.11] | 1.03 [0.95,1.07] | 1.01 [0.95,1.04] | 1.48 |  | 0.059 | 0.156 | 0.366 |
| Rectum | 767 | **1.21 [1.03,1.39]** | **1.23 [1.08,1.36]** | 1.08 [0.99,1.17] | 1.04 [0.97,1.08] | 1.03 [0.98,1.06] | 1.55 |  | 0.0007 | 0.002 | 0.071 |
| Endometrium | 753 | **1.51 [1.30,1.73]** | **1.41 [1.25,1.54]** | 1.07 [0.98,1.15] | **1.09 [1.02,1.15]** | 1.00 [0.95,1.03] | 0.97 |  | <0.00001 | <0.00001 | 0.04577 |
| Cervix uteri | 724 | **1.52 [1.30,1.77]** | **1.14 [1.01,1.28]** | 1.09 [0.98,1.17] | 1.02 [0.95,1.08] | 1.05 [1.00,1.09] | 1.02 |  | 0.00002 | 0.01322 | 0.04785 |
| Brain | 663 | **1.30 [1.06,1.54]** | 1.10 [0.94,1.25] | **1.19 [1.07,1.29]** | **1.11 [1.04,1.16]** | 1.04 [0.99,1.08] | 1.02 |  | 0.00001 | 0.00019 | 0.00006 |
| Melanoma of skin | 618 | 0.90 [0.69,1.13] | 1.06 [0.92,1.20] | **1.15 [1.05,1.25]** | 1.06 [0.99,1.13] | **1.07 [1.01,1.10]** | 0.34 |  | 0.0559 | 0.0044 | 0.0012 |
| Esophagus | 535 | **1.31 [1.08,1.54]** | 1.16 [0.99,1.34] | 1.13 [1.00,1.21] | **1.11 [1.02,1.17]** | **1.06 [1.01,1.10]** | 1.37 |  | 0.00003 | 0.00043 | 0.00104 |
| Diffuse NHL^b^ | 422 | 0.96 [0.73,1.22] | 1.06 [0.89,1.23] | 0.99 [0.86,1.09] | 0.98 [0.89,1.06] | 1.04 [0.97,1.08] | 0.70 |  | 0.579 | 0.493 | 0.643 |
| Multiple myeloma | 391 | **1.33 [1.10,1.62]** | 1.14 [0.95,1.31] | 1.03 [0.91,1.14] | 1.07 [0.98,1.14] | 0.97 [0.91,1.01] | 1.93 |  | 0.026 | 0.218 | 0.424 |
| Lymphoid leukaemia | 368 | 1.26 [0.99,1.58] | 0.93 [0.74,1.12] | **1.20 [1.07,1.33]** | 1.06 [0.96,1.15] | 1.03 [0.96,1.07] | 0.42 |  | 0.029 | 0.104 | 0.010 |
| Myeloid leukaemia | 342 | 1.26 [0.98,1.59] | 1.16 [0.96,1.34] | 1.03 [0.90,1.14] | 0.92 [0.84,1.00] | 1.05 [0.98,1.10] | 1.06 |  | 0.111 | 0.296 | 0.607 |
| Meninges | 291 | 1.22 [0.93,1.55] | **1.25 [1.04,1.48]** | 0.94 [0.79,1.06] | 1.02 [0.92,1.11] | 0.99 [0.92,1.05] | 1.17 |  | 0.148 | 0.290 | 0.801 |
| Liver | 257 | 1.24 [0.98,1.58] | 0.85 [0.67,1.05] | 1.03 [0.89,1.17] | 1.02 [0.91,1.12] | 0.99 [0.91,1.04] | 2.11 |  | 0.449 | 0.788 | 0.508 |
| Lip | 244 | 1.30 [0.99,1.61] | 1.09 [0.86,1.32] | 1.05 [0.90,1.18] | 0.98 [0.88,1.07] | 1.03 [0.96,1.09] | 0.80 |  | 0.128 | 0.365 | 0.450 |
| Hodgkin's disease | 239 | 1.16 [0.76,1.61] | **1.35 [1.10,1.64]** | 0.90 [0.77,1.06] | **1.14 [1.02,1.24]** | 1.07 [0.98,1.13] | 1.18 |  | 0.021 | 0.016 | 0.146 |
| Testis | 222 | 0.82 [0.44,1.35] | **1.35 [1.10,1.63]** | 0.99 [0.83,1.14] | 1.08 [0.95,1.19] | 1.05 [0.96,1.11] | 0.00 |  | 0.171 | 0.026 | 0.242 |
| Larynx | 208 | **1.44 [1.10,1.88]** | 0.97 [0.74,1.22] | **1.31 [1.12,1.47]** | 0.94 [0.84,1.04] | 1.04 [0.94,1.10] | 1.23 |  | 0.025 | 0.126 | 0.051 |
| ^a^ Shown are the estimated risk ratios (RR) with 90% confidence intervals (CI) for 1st to 5th degree (1º-5º) relatives and mates of the 27 cancer sites with ≥200 cases, in bold when the 90% CI does not include 1.00, which corresponds to one-sided p-value <0.05. Also shown are combined p-values (one-sided) to evaluate the significance of the RR for all relatives (1^st^-5^th^ degree) and for close (1^st^ and 2^nd^ degree) and distant relatives (3^rd^-5^th^ degree). | | | | | | | | | | | |
| ^b^ NHL, non-Hodgkin's lymphoma. | | | | | | | | | | | |
| ^c^ na, not applicable (sex-specific cancers). | | | | | | | | | | | |
| ^d^ Number of mates or relatives with cancer/total number of mates or relatives for each cancer site | | | | | | | | | | | |

| **Table 6** RR estimates for relatives and mates of patients diagnosed with colon cancer for sites with 200 or more cases^a^ | | | | | | | | | | | |
| --- | --- | --- | --- | --- | --- | --- | --- | --- | --- | --- | --- |
| Cancer sites | # of aff. | RR [90% CI] | | | | | |  | combined p-value for relatives | | |
|  |  | 1º relative | 2º relatives | 3º relatives | 4º relatives | 5º relatives | Mates |  | 1º-5º | 1º-2º | 3º-5º |
| Breast | 3812 | **1.13 [1.02,1.21]** | 1.06 [0.98,1.11] | 1.03 [0.98,1.07] | 1.04 [0.99,1.06] | **1.07 [1.03,1.08]** | 1.06 |  | 0.00022 | 0.00242 | 0.00269 |
| Prostate | 3380 | **1.15 [1.05,1.23]** | 1.05 [0.97,1.11] | **1.08 [1.02,1.12]** | 1.04 [1.00,1.07] | **1.04 [1.01,1.06]** | 1.44 |  | 0.00002 | 0.00105 | 0.00059 |
| Lung | 2904 | **1.17 [1.06,1.27]** | 1.03 [0.95,1.11] | **1.07 [1.02,1.12]** | 1.02 [0.98,1.06] | 1.01 [0.99,1.04] | 1.38 |  | 0.0014 | 0.0247 | 0.0152 |
| Stomach | 2890 | **1.26 [1.14,1.36]** | 1.08 [0.99,1.15] | **1.10 [1.04,1.14]** | **1.06 [1.01,1.08]** | 1.04 [1.00,1.05] | 1.37 |  | <0.00001 | 0.00032 | 0.00049 |
| Colon | 2224 | **1.92 [1.71,2.14]** | **1.26 [1.12,1.40]** | **1.16 [1.07,1.24]** | 1.05 [0.98,1.10] | **1.06 [1.01,1.09]** | 1.46 |  | <0.00001 | <0.00001 | 0.00029 |
| Bladder | 1384 | 1.16 [1.00,1.29] | 1.04 [0.92,1.14] | 1.06 [0.97,1.12] | 1.01 [0.95,1.06] | 1.02 [0.98,1.06] | 0.83 |  | 0.052 | 0.177 | 0.197 |
| Kidney | 1227 | **1.18 [1.03,1.36]** | 0.99 [0.88,1.11] | **1.13 [1.04,1.21]** | 1.02 [0.95,1.06] | 1.00 [0.95,1.03] | 1.75 |  | 0.04145 | 0.18805 | 0.0915 |
| Thyroid | 957 | **1.30 [1.09,1.50]** | **1.16 [1.03,1.30]** | 1.08 [0.98,1.16] | 1.01 [0.95,1.06] | 1.05 [1.00,1.09] | 1.12 |  | 0.00062 | 0.01209 | 0.07399 |
| Pancreas | 930 | **1.25 [1.07,1.45]** | 1.07 [0.93,1.22] | 0.94 [0.86,1.02] | 0.99 [0.92,1.04] | 1.03 [0.98,1.07] | 1.55 |  | 0.106 | 0.517 | 0.733 |
| Ovary | 906 | **1.26 [1.05,1.48]** | 1.09 [0.94,1.25] | 1.02 [0.92,1.10] | 0.98 [0.91,1.04] | 0.99 [0.94,1.02] | 1.04 |  | 0.104 | 0.486 | 0.745 |
| Non-melanoma skin | 781 | **1.29 [1.05,1.48]** | 1.02 [0.87,1.17] | 0.98 [0.89,1.07] | 0.96 [0.89,1.01] | 1.00 [0.95,1.04] | 1.21 |  | 0.352 | 0.823 | 0.893 |
| Rectum | 767 | **1.64 [1.41,1.89]** | **1.27 [1.11,1.43]** | **1.13 [1.02,1.23]** | **1.10 [1.02,1.16]** | **1.06 [1.01,1.10]** | 1.50 |  | <0.00001 | 0.00001 | 0.00074 |
| Endometrium | 753 | **1.33 [1.11,1.54]** | 1.12 [0.95,1.26] | 1.07 [0.97,1.17] | 0.97 [0.89,1.04] | 1.02 [0.96,1.06] | 1.75 |  | 0.010 | 0.200 | 0.404 |
| Cervix uteri | 724 | 1.14 [0.91,1.39] | 0.94 [0.80,1.09] | 0.95 [0.84,1.04] | 1.02 [0.93,1.07] | 1.00 [0.95,1.04] | 1.75 |  | 0.649 | 0.864 | 0.805 |
| Brain | 663 | 0.94 [0.72,1.21] | 0.92 [0.79,1.10] | 1.03 [0.92,1.14] | 0.99 [0.92,1.07] | 0.99 [0.94,1.04] | 0.96 |  | 0.765 | 0.711 | 0.536 |
| Melanoma of skin | 618 | 1.07 [0.83,1.35] | 1.11 [0.94,1.27] | 1.08 [0.96,1.18] | 1.03 [0.94,1.10] | 1.00 [0.94,1.04] | 1.45 |  | 0.132 | 0.141 | 0.272 |
| Esophagus | 535 | 1.01 [0.80,1.26] | 0.87 [0.71,1.02] | 0.99 [0.88,1.11] | 1.05 [0.95,1.11] | 0.97 [0.90,1.01] | 1.37 |  | 0.838 | 0.895 | 0.693 |
| Diffuse NHL^b^ | 422 | 1.10 [0.83,1.40] | 1.01 [0.82,1.23] | 1.10 [0.97,1.23] | 1.06 [0.95,1.14] | 1.05 [0.98,1.11] | 1.49 |  | 0.101 | 0.104 | 0.049 |
| Multiple myeloma | 391 | 0.99 [0.75,1.28] | 0.93 [0.75,1.15] | 1.10 [0.97,1.24] | 0.93 [0.83,1.00] | 0.92 [0.86,0.97] | 0.66 |  | 0.895 | 0.908 | 0.900 |
| Lymphoid leukaemia | 368 | 1.01 [0.71,1.33] | 0.69 [0.51,0.86] | 0.95 [0.81,1.09] | 0.97 [0.87,1.06] | 1.00 [0.93,1.06] | 1.05 |  | 0.973 | 0.993 | 0.829 |
| Myeloid leukaemia | 342 | 1.03 [0.75,1.40] | 1.10 [0.89,1.34] | 0.95 [0.80,1.08] | 0.91 [0.80,0.99] | 0.94 [0.88,1.01] | 1.46 |  | 0.832 | 0.895 | 0.989 |
| Meninges | 291 | 1.27 [0.93,1.66] | 0.95 [0.73,1.19] | 1.01 [0.85,1.15] | 1.04 [0.92,1.14] | **1.09 [1.01,1.16]** | 2.03 |  | 0.111 | 0.281 | 0.153 |
| Liver | 257 | 1.22 [0.89,1.64] | 1.08 [0.82,1.35] | 0.96 [0.80,1.12] | 0.92 [0.81,1.03] | 1.04 [0.94,1.10] | 1.00 |  | 0.416 | 0.660 | 0.806 |
| Lip | 244 | 0.85 [0.57,1.22] | 0.94 [0.69,1.22] | 0.94 [0.80,1.10] | 1.01 [0.89,1.12] | 0.95 [0.87,1.01] | 1.83 |  | 0.902 | 0.866 | 0.855 |
| Hodgkin's disease | 239 | 1.07 [0.71,1.60] | 1.27 [0.98,1.60] | 1.18 [0.96,1.36] | 1.06 [0.93,1.17] | 1.00 [0.92,1.07] | 1.21 |  | 0.061 | 0.031 | 0.128 |
| Testis | 222 | 1.40 [0.90,2.16] | 0.96 [0.70,1.22] | **1.33 [1.10,1.56]** | 0.95 [0.82,1.09] | 1.04 [0.94,1.12] | 0.00 |  | 0.064 | 0.165 | 0.069 |
| Larynx | 208 | 0.82 [0.52,1.20] | 1.27 [0.95,1.57] | 0.99 [0.81,1.17] | 0.89 [0.77,1.02] | 1.01 [0.91,1.09] | 0.56 |  | 0.636 | 0.433 | 0.850 |
| ^a^ Shown are the estimated risk ratios (RR) with 90% confidence intervals (CI) for 1st to 5th degree (1º-5º) relatives and mates of the 27 cancer sites with ≥200 cases, in bold when the 90% CI does not include 1.00, which corresponds to one-sided p-value <0.05. Also shown are combined p-values (one-sided) to evaluate the significance of the RR for all relatives (1^st^-5^th^ degree) and for close (1^st^ and 2^nd^ degree) and distant relatives (3^rd^-5^th^ degree). | | | | | | | | | | | |
| ^b^ NHL, non-Hodgkin's lymphoma. | | | | | | | | | | | |
| ^c^ na, not applicable (sex-specific cancers). | | | | | | | | | | | |
| ^d^ Number of mates or relatives with cancer/total number of mates or relatives for each cancer site | | | | | | | | | | | |

| **Table 7** RR estimates for relatives and mates of patients diagnosed with bladder cancer for sites with 200 or more cases^a^ | | | | | | | | | | | |
| --- | --- | --- | --- | --- | --- | --- | --- | --- | --- | --- | --- |
| Cancer sites | # of aff. | RR [90% CI] | | | | | |  | combined p-value for relatives | | |
|  |  | 1º relative | 2º relatives | 3º relatives | 4º relatives | 5º relatives | Mates |  | 1º-5º | 1º-2º | 3º-5º |
| Breast | 3812 | 1.07 [0.95,1.18] | 1.10 [1.00,1.18] | 1.05 [0.99,1.11] | 1.00 [0.94,1.02] | 1.02 [0.98,1.04] | 1.28 |  | 0.050 | 0.082 | 0.300 |
| Prostate | 3380 | **1.34 [1.20,1.46]** | **1.12 [1.02,1.21]** | 1.04 [0.97,1.09] | **1.07 [1.02,1.10]** | 1.01 [0.97,1.03] | 1.10 |  | <0.00001 | 0.0062 | 0.04423 |
| Lung | 2904 | **1.27 [1.15,1.43]** | **1.12 [1.02,1.23]** | 1.05 [0.99,1.11] | 1.00 [0.95,1.05] | 1.00 [0.98,1.04] | 1.35 |  | 0.00033 | 0.03663 | 0.18905 |
| Stomach | 2890 | **1.20 [1.06,1.33]** | 1.08 [0.96,1.16] | 1.07 [0.99,1.12] | 1.05 [0.99,1.09] | 1.03 [0.99,1.05] | 1.31 |  | 0.00121 | 0.01464 | 0.02383 |
| Colon | 2224 | 1.16 [1.00,1.29] | 1.04 [0.92,1.14] | 1.06 [0.97,1.12] | 1.01 [0.95,1.06] | 1.02 [0.98,1.06] | 0.83 |  | 0.052 | 0.177 | 0.197 |
| Bladder | 1384 | **1.68 [1.39,2.05]** | 1.19 [0.98,1.43] | **1.26 [1.13,1.41]** | 1.08 [0.99,1.19] | 1.03 [0.97,1.10] | 0.41 |  | <0.00001 | 0.00002 | 0.00033 |
| Kidney | 1227 | **1.24 [1.05,1.47]** | 1.06 [0.92,1.24] | 1.01 [0.91,1.13] | 0.99 [0.92,1.06] | 1.00 [0.95,1.05] | 1.76 |  | 0.086 | 0.336 | 0.487 |
| Thyroid | 957 | 1.22 [0.96,1.46] | **1.20 [1.04,1.42]** | 0.99 [0.89,1.11] | 1.05 [0.98,1.13] | 1.03 [0.98,1.08] | 1.68 |  | 0.0076 | 0.02385 | 0.1575 |
| Pancreas | 930 | **1.36 [1.13,1.62]** | 1.16 [0.99,1.37] | 1.07 [0.96,1.18] | 1.00 [0.93,1.09] | 0.99 [0.93,1.05] | 1.16 |  | 0.00435 | 0.06895 | 0.26254 |
| Ovary | 906 | 1.03 [0.83,1.30] | **1.29 [1.11,1.52]** | 1.10 [1.00,1.24] | 0.94 [0.87,1.03] | 0.97 [0.93,1.04] | 1.43 |  | 0.049 | 0.035 | 0.479 |
| Non-melanoma skin | 781 | 1.06 [0.83,1.32] | **1.24 [1.06,1.47]** | 1.00 [0.90,1.12] | 0.98 [0.90,1.07] | 1.00 [0.95,1.06] | 1.57 |  | 0.084 | 0.076 | 0.530 |
| Rectum | 767 | 0.90 [0.72,1.14] | 1.08 [0.92,1.32] | 1.02 [0.91,1.15] | 0.89 [0.81,0.97] | 0.98 [0.92,1.04] | 1.41 |  | 0.750 | 0.646 | 0.868 |
| Endometrium | 753 | 1.09 [0.86,1.39] | 0.95 [0.77,1.15] | 0.97 [0.85,1.10] | 1.09 [1.00,1.19] | **1.07 [1.01,1.14]** | 1.12 |  | 0.136 | 0.159 | 0.049 |
| Cervix uteri | 724 | **1.32 [1.05,1.67]** | 1.00 [0.84,1.24] | 0.91 [0.79,1.03] | 0.95 [0.87,1.04] | 1.00 [0.94,1.07] | 1.98 |  | 0.304 | 0.790 | 0.911 |
| Brain | 663 | **1.43 [1.15,1.88]** | 0.96 [0.78,1.21] | 0.95 [0.81,1.09] | 1.07 [0.97,1.17] | **1.08 [1.02,1.15]** | 0.00 |  | 0.016 | 0.213 | 0.105 |
| Melanoma of skin | 618 | 1.02 [0.75,1.38] | 1.05 [0.86,1.30] | 1.10 [0.98,1.25] | 1.01 [0.92,1.10] | 0.97 [0.91,1.04] | 0.56 |  | 0.255 | 0.235 | 0.268 |
| Esophagus | 535 | 1.17 [0.90,1.51] | 0.91 [0.74,1.15] | 0.98 [0.83,1.13] | 1.05 [0.96,1.16] | 1.01 [0.95,1.08] | 1.31 |  | 0.247 | 0.484 | 0.315 |
| Diffuse NHL^b^ | 422 | 1.04 [0.72,1.46] | 0.91 [0.67,1.21] | **1.22 [1.06,1.42]** | 0.95 [0.83,1.06] | 1.03 [0.96,1.12] | 0.92 |  | 0.258 | 0.241 | 0.086 |
| Multiple myeloma | 391 | 0.75 [0.50,1.06] | 0.95 [0.69,1.27] | 1.16 [0.99,1.35] | 1.02 [0.90,1.15] | 0.95 [0.88,1.04] | 0.00 |  | 0.643 | 0.343 | 0.215 |
| Lymphoid leukaemia | 368 | 1.03 [0.68,1.48] | **1.27 [1.01,1.63]** | 1.18 [1.00,1.38] | 0.96 [0.84,1.09] | 1.06 [0.98,1.15] | 1.41 |  | 0.045 | 0.022 | 0.089 |
| Myeloid leukaemia | 342 | **1.46 [1.05,2.06]** | 0.90 [0.67,1.22] | **1.29 [1.13,1.55]** | 0.96 [0.84,1.10] | 0.99 [0.90,1.08] | 0.41 |  | 0.030 | 0.200 | 0.072 |
| Meninges | 291 | 0.71 [0.42,1.13] | 1.11 [0.81,1.48] | 1.06 [0.88,1.28] | 1.06 [0.93,1.22] | **1.15 [1.06,1.25]** | 0.72 |  | 0.189 | 0.024 | 0.019 |
| Liver | 257 | 1.23 [0.85,1.81] | 1.17 [0.87,1.55] | 1.14 [0.93,1.38] | 1.03 [0.90,1.21] | 0.99 [0.90,1.10] | 0.94 |  | 0.050 | 0.107 | 0.188 |
| Lip | 244 | 0.84 [0.50,1.30] | 1.11 [0.83,1.51] | 1.06 [0.87,1.28] | 1.04 [0.88,1.20] | 1.02 [0.92,1.13] | 3.21 |  | 0.324 | 0.170 | 0.253 |
| Hodgkin's disease | 239 | 1.21 [0.72,1.95] | 0.96 [0.68,1.32] | 0.80 [0.62,1.03] | 0.91 [0.77,1.08] | 1.09 [0.98,1.21] | 0.00 |  | 0.555 | 0.764 | 0.779 |
| Testis | 222 | 1.22 [0.66,2.20] | 1.28 [0.94,1.71] | 0.99 [0.77,1.28] | 0.98 [0.83,1.15] | 1.02 [0.91,1.14] | 0.00 |  | 0.137 | 0.187 | 0.477 |
| Larynx | 208 | 1.15 [0.74,1.74] | 0.94 [0.63,1.40] | 0.87 [0.69,1.11] | 1.05 [0.91,1.26] | 1.05 [0.95,1.17] | 1.63 |  | 0.347 | 0.452 | 0.387 |
| ^a^ Shown are the estimated risk ratios (RR) with 90% confidence intervals (CI) for 1st to 5th degree (1º-5º) relatives and mates of the 27 cancer sites with ≥200 cases, in bold when the 90% CI does not include 1.00, which corresponds to one-sided p-value <0.05. Also shown are combined p-values (one-sided) to evaluate the significance of the RR for all relatives (1^st^-5^th^ degree) and for close (1^st^ and 2^nd^ degree) and distant relatives (3^rd^-5^th^ degree). | | | | | | | | | | | |
| ^b^ NHL, non-Hodgkin's lymphoma. | | | | | | | | | | | |
| ^c^ na, not applicable (sex-specific cancers). | | | | | | | | | | | |
| ^d^ Number of mates or relatives with cancer/total number of mates or relatives for each cancer site | | | | | | | | | | | |

| **Table 8** RR estimates for relatives and mates of patients diagnosed with kidney cancer for sites with 200 or more cases^a^ | | | | | | | | | | | |
| --- | --- | --- | --- | --- | --- | --- | --- | --- | --- | --- | --- |
| Cancer sites | # of aff. | RR [90% CI] | | | | | |  | combined p-value for relatives | | |
|  |  | 1º relative | 2º relatives | 3º relatives | 4º relatives | 5º relatives | Mates |  | 1º-5º | 1º-2º | 3º-5º |
| Breast | 3812 | 1.06 [0.94,1.20] | **1.12 [1.02,1.20]** | 1.03 [0.96,1.08] | 1.01 [0.96,1.04] | 1.04 [1.00,1.06] | 0.93 |  | 0.02958 | 0.0377 | 0.21246 |
| Prostate | 3380 | **1.31 [1.16,1.43]** | **1.18 [1.07,1.28]** | 1.07 [1.00,1.12] | 1.05 [0.99,1.08] | 1.04 [1.00,1.06] | 1.75 |  | 0.00001 | 0.00014 | 0.0086 |
| Lung | 2904 | 1.11 [0.99,1.26] | 1.09 [0.98,1.20] | 1.00 [0.94,1.07] | 0.99 [0.93,1.03] | 0.95 [0.92,0.98] | 0.96 |  | 0.325 | 0.631 | 0.907 |
| Stomach | 2890 | **1.19 [1.03,1.32]** | 1.02 [0.90,1.11] | **1.13 [1.04,1.18]** | 1.07 [1.00,1.10] | 1.04 [1.00,1.07] | 1.08 |  | 0.00063 | 0.00461 | 0.00058 |
| Colon | 2224 | **1.18 [1.03,1.36]** | 0.99 [0.88,1.11] | **1.13 [1.04,1.21]** | 1.02 [0.95,1.06] | 1.00 [0.95,1.03] | 1.75 |  | 0.04145 | 0.18805 | 0.0915 |
| Bladder | 1384 | **1.24 [1.05,1.47]** | 1.06 [0.92,1.24] | 1.01 [0.91,1.13] | 0.99 [0.92,1.06] | 1.00 [0.95,1.05] | 1.76 |  | 0.086 | 0.336 | 0.487 |
| Kidney | 1227 | **2.30 [1.89,2.80]** | **1.31 [1.06,1.57]** | **1.32 [1.15,1.48]** | **1.15 [1.03,1.26]** | 1.03 [0.95,1.09] | 1.20 |  | <0.00001 | <0.00001 | 0.00034 |
| Thyroid | 957 | **1.37 [1.09,1.69]** | **1.22 [1.02,1.42]** | **1.17 [1.03,1.29]** | 1.10 [0.99,1.17] | 1.00 [0.94,1.05] | 0.95 |  | 0.00016 | 0.00353 | 0.02136 |
| Pancreas | 930 | 1.10 [0.88,1.37] | 0.95 [0.76,1.13] | 1.12 [0.98,1.24] | 0.95 [0.86,1.03] | 1.08 [1.00,1.13] | 1.80 |  | 0.234 | 0.329 | 0.153 |
| Ovary | 906 | 1.25 [1.00,1.58] | 0.93 [0.76,1.10] | 1.05 [0.92,1.16] | 1.07 [0.98,1.16] | 1.06 [0.99,1.11] | 1.19 |  | 0.073 | 0.231 | 0.064 |
| Non-melanoma skin | 781 | 1.15 [0.88,1.44] | 1.04 [0.85,1.23] | 1.10 [0.98,1.24] | 1.05 [0.94,1.12] | 1.08 [1.00,1.14] | 1.63 |  | 0.041 | 0.060 | 0.035 |
| Rectum | 767 | 1.22 [0.97,1.52] | 1.18 [0.96,1.40] | 0.85 [0.73,0.97] | 0.98 [0.88,1.07] | 1.05 [0.98,1.11] | 0.93 |  | 0.211 | 0.521 | 0.883 |
| Endometrium | 753 | **1.47 [1.14,1.77]** | 1.13 [0.93,1.39] | 1.09 [0.95,1.21] | 1.10 [1.00,1.20] | 1.04 [0.96,1.09] | 0.67 |  | 0.0018 | 0.0378 | 0.0619 |
| Cervix uteri | 724 | 0.88 [0.66,1.22] | 1.09 [0.89,1.33] | **1.18 [1.03,1.33]** | 0.97 [0.87,1.06] | 1.04 [0.96,1.10] | 1.89 |  | 0.222 | 0.092 | 0.123 |
| Brain | 663 | **1.41 [1.08,1.84]** | **1.25 [1.02,1.49]** | **1.23 [1.07,1.38]** | 1.06 [0.95,1.14] | 1.06 [0.98,1.12] | 0.30 |  | 0.00029 | 0.00232 | 0.00986 |
| Melanoma of skin | 618 | **1.52 [1.10,2.00]** | **1.31 [1.04,1.58]** | **1.29 [1.12,1.45]** | 1.06 [0.95,1.15] | **1.10 [1.01,1.17]** | 1.72 |  | 0.00001 | 0.00008 | 0.00071 |
| Esophagus | 535 | 1.07 [0.76,1.44] | 1.18 [0.90,1.42] | **1.18 [1.01,1.34]** | 0.97 [0.86,1.07] | 1.04 [0.95,1.10] | 1.14 |  | 0.106 | 0.107 | 0.161 |
| Diffuse NHL^b^ | 422 | **1.37 [1.02,1.93]** | **1.33 [1.01,1.66]** | 1.16 [0.96,1.34] | 1.14 [1.00,1.26] | 1.10 [1.00,1.18] | 0.73 |  | 0.00051 | 0.00099 | 0.00573 |
| Multiple myeloma | 391 | **1.71 [1.29,2.24]** | 1.17 [0.88,1.48] | 0.95 [0.77,1.12] | 1.14 [0.99,1.26] | 1.07 [0.96,1.14] | 1.29 |  | 0.0038 | 0.117 | 0.1863 |
| Lymphoid leukaemia | 368 | 1.33 [0.87,1.84] | 0.90 [0.66,1.21] | 1.08 [0.89,1.28] | 1.13 [0.98,1.26] | 1.06 [0.97,1.15] | 1.03 |  | 0.076 | 0.203 | 0.050 |
| Myeloid leukaemia | 342 | 1.35 [0.91,1.91] | 1.04 [0.78,1.35] | 1.05 [0.86,1.27] | **1.24 [1.09,1.39]** | 1.09 [0.98,1.17] | 1.42 |  | 0.0084 | 0.0267 | 0.0096 |
| Meninges | 291 | 1.14 [0.71,1.67] | 1.05 [0.75,1.36] | 1.06 [0.86,1.28] | 0.99 [0.83,1.14] | 1.05 [0.95,1.14] | 0.99 |  | 0.284 | 0.326 | 0.337 |
| Liver | 257 | 0.80 [0.48,1.33] | 0.90 [0.61,1.28] | 1.09 [0.84,1.33] | 0.93 [0.78,1.09] | 1.00 [0.89,1.09] | 1.59 |  | 0.749 | 0.679 | 0.588 |
| Lip | 244 | 0.79 [0.47,1.34] | 0.89 [0.60,1.25] | 0.93 [0.71,1.11] | 1.09 [0.94,1.26] | 1.00 [0.88,1.09] | 0.62 |  | 0.807 | 0.706 | 0.547 |
| Hodgkin's disease | 239 | 0.39 [0.13,0.87] | **1.42 [1.04,1.91]** | 1.12 [0.89,1.38] | 1.03 [0.86,1.21] | 1.02 [0.89,1.12] | 0.00 |  | 0.442 | 0.047 | 0.263 |
| Testis | 222 | 1.62 [0.96,2.58] | 0.81 [0.52,1.24] | 1.27 [1.00,1.59] | 0.97 [0.80,1.15] | 1.05 [0.92,1.16] | 0.00 |  | 0.101 | 0.330 | 0.138 |
| Larynx | 208 | 1.26 [0.81,1.97] | 1.08 [0.75,1.54] | 1.16 [0.90,1.42] | 0.79 [0.63,0.95] | **1.18 [1.06,1.30]** | 0.59 |  | 0.156 | 0.254 | 0.271 |
| ^a^ Shown are the estimated risk ratios (RR) with 90% confidence intervals (CI) for 1st to 5th degree (1º-5º) relatives and mates of the 27 cancer sites with ≥200 cases, in bold when the 90% CI does not include 1.00, which corresponds to one-sided p-value <0.05. Also shown are combined p-values (one-sided) to evaluate the significance of the RR for all relatives (1^st^-5^th^ degree) and for close (1^st^ and 2^nd^ degree) and distant relatives (3^rd^-5^th^ degree). | | | | | | | | | | | |
| ^b^ NHL, non-Hodgkin's lymphoma. | | | | | | | | | | | |
| ^c^ na, not applicable (sex-specific cancers). | | | | | | | | | | | |
| ^d^ Number of mates or relatives with cancer/total number of mates or relatives for each cancer site | | | | | | | | | | | |

| **Table 9** RR estimates for relatives and mates of patients diagnosed with thyroid cancer for sites with 200 or more cases^a^ | | | | | | | | | | | |
| --- | --- | --- | --- | --- | --- | --- | --- | --- | --- | --- | --- |
| Cancer sites | # of aff. | RR [90% CI] | | | | | |  | combined p-value for relatives | | |
|  |  | 1º relative | 2º relatives | 3º relatives | 4º relatives | 5º relatives | Mates |  | 1º-5º | 1º-2º | 3º-5º |
| Breast | 3812 | **1.21 [1.06,1.37]** | 1.03 [0.92,1.13] | 1.01 [0.93,1.06] | 1.04 [0.98,1.07] | 1.01 [0.96,1.03] | 1.17 |  | 0.05065 | 0.35495 | 0.3868 |
| Prostate | 3380 | **1.30 [1.14,1.45]** | **1.19 [1.07,1.30]** | 1.06 [0.98,1.12] | **1.07 [1.01,1.11]** | 1.04 [1.00,1.07] | 1.42 |  | <0.00001 | 0.00018 | 0.0061 |
| Lung | 2904 | 0.99 [0.84,1.18] | 0.96 [0.85,1.07] | 1.03 [0.96,1.13] | 1.02 [0.97,1.08] | 1.00 [0.96,1.04] | 1.17 |  | 0.406 | 0.365 | 0.183 |
| Stomach | 2890 | 1.16 [0.99,1.33] | **1.25 [1.12,1.36]** | **1.15 [1.06,1.22]** | 1.06 [0.99,1.10] | 1.03 [0.98,1.05] | 1.66 |  | 0.00003 | 0.00003 | 0.00199 |
| Colon | 2224 | **1.30 [1.09,1.50]** | **1.16 [1.03,1.30]** | 1.08 [0.98,1.16] | 1.01 [0.95,1.06] | 1.05 [1.00,1.09] | 1.12 |  | 0.00062 | 0.01209 | 0.07399 |
| Bladder | 1384 | 1.22 [0.96,1.46] | **1.20 [1.04,1.42]** | 0.99 [0.89,1.11] | 1.05 [0.98,1.13] | 1.03 [0.98,1.08] | 1.68 |  | 0.0076 | 0.02385 | 0.1575 |
| Kidney | 1227 | **1.37 [1.09,1.69]** | **1.22 [1.02,1.42]** | **1.17 [1.03,1.29]** | 1.10 [0.99,1.17] | 1.00 [0.94,1.05] | 0.95 |  | 0.00016 | 0.00353 | 0.02136 |
| Thyroid | 957 | **3.02 [2.33,3.85]** | **1.64 [1.29,2.02]** | **1.30 [1.07,1.51]** | 1.08 [0.93,1.21] | 1.11 [1.00,1.19] | 1.12 |  | <0.00001 | <0.00001 | 0.00506 |
| Pancreas | 930 | 0.98 [0.69,1.26] | **1.37 [1.13,1.60]** | 0.99 [0.85,1.12] | 0.99 [0.88,1.07] | 1.09 [1.00,1.14] | 0.83 |  | 0.077 | 0.027 | 0.391 |
| Ovary | 906 | 1.17 [0.86,1.55] | 0.98 [0.77,1.18] | 1.07 [0.92,1.19] | 1.09 [0.98,1.17] | 1.05 [0.97,1.10] | 1.25 |  | 0.135 | 0.211 | 0.087 |
| Non-melanoma skin | 781 | 0.98 [0.71,1.30] | 1.00 [0.78,1.22] | **1.17 [1.02,1.34]** | 1.09 [0.96,1.18] | 1.04 [0.95,1.09] | 1.56 |  | 0.161 | 0.089 | 0.040 |
| Rectum | 767 | 1.30 [0.99,1.70] | **1.44 [1.16,1.72]** | 0.91 [0.76,1.04] | 1.04 [0.92,1.13] | 0.99 [0.90,1.04] | 1.07 |  | 0.033 | 0.151 | 0.860 |
| Endometrium | 753 | **1.45 [1.12,1.84]** | 1.28 [1.00,1.54] | 1.08 [0.89,1.22] | 1.00 [0.88,1.09] | 1.01 [0.92,1.06] | 0.95 |  | 0.026 | 0.168 | 0.540 |
| Cervix uteri | 724 | 1.12 [0.83,1.56] | 1.22 [0.97,1.50] | 1.15 [0.97,1.32] | 1.08 [0.94,1.17] | 1.02 [0.93,1.07] | 0.41 |  | 0.036 | 0.048 | 0.113 |
| Brain | 663 | **1.51 [1.09,2.03]** | 0.90 [0.69,1.17] | 0.98 [0.82,1.13] | **1.23 [1.07,1.34]** | 1.10 [0.99,1.16] | 1.22 |  | 0.019 | 0.119 | 0.029 |
| Melanoma of skin | 618 | **1.62 [1.17,2.24]** | 0.73 [0.52,0.95] | 1.15 [0.97,1.32] | 1.05 [0.92,1.15] | 0.95 [0.86,1.02] | 1.01 |  | 0.212 | 0.788 | 0.344 |
| Esophagus | 535 | 0.91 [0.56,1.34] | 1.21 [0.93,1.52] | 0.90 [0.73,1.05] | 0.96 [0.82,1.06] | 0.99 [0.89,1.05] | 0.78 |  | 0.755 | 0.711 | 0.945 |
| Diffuse NHL^b^ | 422 | 1.17 [0.75,1.72] | 0.67 [0.44,0.93] | 1.06 [0.86,1.27] | 1.10 [0.95,1.25] | 0.90 [0.79,0.97] | 2.44 |  | 0.808 | 0.933 | 0.600 |
| Multiple myeloma | 391 | **1.65 [1.17,2.36]** | 1.21 [0.90,1.60] | 1.18 [0.97,1.40] | 0.82 [0.67,0.94] | 1.00 [0.89,1.08] | 0.47 |  | 0.082 | 0.445 | 0.750 |
| Lymphoid leukaemia | 368 | **1.57 [1.02,2.27]** | 0.92 [0.62,1.24] | 0.93 [0.73,1.10] | 0.98 [0.81,1.10] | 1.01 [0.89,1.09] | 2.12 |  | 0.390 | 0.859 | 0.833 |
| Myeloid leukaemia | 342 | 1.08 [0.64,1.68] | 1.13 [0.79,1.49] | 1.12 [0.86,1.35] | 1.02 [0.86,1.17] | 1.09 [0.97,1.19] | 1.97 |  | 0.126 | 0.148 | 0.168 |
| Meninges | 291 | **1.82 [1.17,2.73]** | 1.29 [0.92,1.70] | 1.16 [0.91,1.39] | 1.04 [0.87,1.20] | 1.01 [0.88,1.10] | 0.91 |  | 0.012 | 0.135 | 0.298 |
| Liver | 257 | 1.49 [0.88,2.48] | 1.03 [0.68,1.44] | 1.20 [0.93,1.50] | 0.85 [0.68,1.02] | 1.11 [0.98,1.21] | 1.37 |  | 0.108 | 0.322 | 0.335 |
| Lip | 244 | 1.06 [0.59,1.69] | 1.04 [0.72,1.47] | 0.94 [0.71,1.19] | 1.17 [0.97,1.35] | 1.13 [0.99,1.26] | 0.67 |  | 0.214 | 0.160 | 0.118 |
| Hodgkin's disease | 239 | 1.22 [0.58,2.52] | 1.04 [0.69,1.47] | **1.48 [1.16,1.86]** | 1.17 [0.97,1.36] | 0.94 [0.81,1.06] | 1.26 |  | 0.061 | 0.061 | 0.021 |
| Testis | 222 | 1.68 [0.86,3.01] | 1.46 [0.94,1.99] | **1.40 [1.06,1.73]** | 1.14 [0.94,1.34] | 0.95 [0.81,1.06] | 1.34 |  | 0.009 | 0.016 | 0.070 |
| Larynx | 208 | 0.38 [0.13,1.02] | 0.87 [0.55,1.32] | 0.90 [0.63,1.17] | 0.96 [0.77,1.13] | **1.27 [1.11,1.42]** | 0.67 |  | 0.802 | 0.454 | 0.270 |
| ^a^ Shown are the estimated risk ratios (RR) with 90% confidence intervals (CI) for 1st to 5th degree (1º-5º) relatives and mates of the 27 cancer sites with ≥200 cases, in bold when the 90% CI does not include 1.00, which corresponds to one-sided p-value <0.05. Also shown are combined p-values (one-sided) to evaluate the significance of the RR for all relatives (1^st^-5^th^ degree) and for close (1^st^ and 2^nd^ degree) and distant relatives (3^rd^-5^th^ degree). | | | | | | | | | | | |
| ^b^ NHL, non-Hodgkin's lymphoma. | | | | | | | | | | | |
| ^c^ na, not applicable (sex-specific cancers). | | | | | | | | | | | |
| ^d^ Number of mates or relatives with cancer/total number of mates or relatives for each cancer site | | | | | | | | | | | |

| **Table 10** RR estimates for relatives and mates of patients diagnosed with pancreatic cancer for sites with 200 or more cases^a^ | | | | | | | | | | | |
| --- | --- | --- | --- | --- | --- | --- | --- | --- | --- | --- | --- |
| Cancer sites | # of aff. | RR [90% CI] | | | | | |  | combined p-value for relatives | | |
|  |  | 1º relative | 2º relatives | 3º relatives | 4º relatives | 5º relatives | Mates |  | 1º-5º | 1º-2º | 3º-5º |
| Breast | 3812 | 1.11 [0.96,1.24] | **1.12 [1.01,1.22]** | 1.04 [0.96,1.10] | 1.03 [0.97,1.06] | 1.00 [0.96,1.02] | 1.05 |  | 0.04072 | 0.09043 | 0.36245 |
| Prostate | 3380 | 1.13 [0.98,1.25] | 1.12 [1.00,1.22] | 1.04 [0.96,1.10] | 1.06 [1.00,1.10] | **1.06 [1.02,1.08]** | 1.03 |  | 0.0008 | 0.0021 | 0.0095 |
| Lung | 2904 | **1.20 [1.05,1.37]** | 1.09 [0.97,1.21] | 1.01 [0.94,1.08] | **1.08 [1.03,1.14]** | 1.02 [0.99,1.06] | 1.06 |  | 0.00148 | 0.01326 | 0.02842 |
| Stomach | 2890 | **1.23 [1.07,1.39]** | 1.12 [0.98,1.23] | 1.07 [0.98,1.14] | 1.00 [0.93,1.04] | **1.07 [1.02,1.10]** | 1.58 |  | 0.0013 | 0.0205 | 0.0654 |
| Colon | 2224 | **1.25 [1.07,1.45]** | 1.07 [0.93,1.22] | 0.94 [0.86,1.02] | 0.99 [0.92,1.04] | 1.03 [0.98,1.07] | 1.55 |  | 0.106 | 0.517 | 0.733 |
| Bladder | 1384 | **1.36 [1.13,1.62]** | 1.16 [0.99,1.37] | 1.07 [0.96,1.18] | 1.00 [0.93,1.09] | 0.99 [0.93,1.05] | 1.16 |  | 0.00435 | 0.06895 | 0.26254 |
| Kidney | 1227 | 1.10 [0.88,1.37] | 0.95 [0.76,1.13] | 1.12 [0.98,1.24] | 0.95 [0.86,1.03] | 1.08 [1.00,1.13] | 1.80 |  | 0.234 | 0.329 | 0.153 |
| Thyroid | 957 | 0.98 [0.69,1.26] | **1.37 [1.13,1.60]** | 0.99 [0.85,1.12] | 0.99 [0.88,1.07] | 1.09 [1.00,1.14] | 0.83 |  | 0.077 | 0.027 | 0.391 |
| Pancreas | 930 | **2.33 [1.83,2.96]** | 1.28 [0.97,1.66] | 1.09 [0.90,1.29] | 1.06 [0.93,1.21] | 0.99 [0.91,1.08] | 1.29 |  | 0.00001 | <0.00001 | 0.20677 |
| Ovary | 906 | **1.66 [1.31,2.05]** | 1.17 [0.98,1.42] | **1.22 [1.09,1.40]** | 1.09 [0.98,1.19] | 1.03 [0.96,1.10] | 2.17 |  | 0.00003 | 0.00197 | 0.00353 |
| Non-melanoma skin | 781 | 1.29 [1.00,1.64] | 1.02 [0.80,1.30] | 1.04 [0.89,1.20] | 0.97 [0.87,1.08] | 0.98 [0.90,1.04] | 0.87 |  | 0.241 | 0.569 | 0.650 |
| Rectum | 767 | 1.08 [0.76,1.40] | 1.18 [0.96,1.44] | 0.87 [0.73,1.01] | 1.05 [0.95,1.18] | 1.01 [0.94,1.07] | 2.18 |  | 0.289 | 0.307 | 0.658 |
| Endometrium | 753 | 0.99 [0.75,1.34] | 1.10 [0.88,1.36] | 1.04 [0.89,1.19] | 0.96 [0.86,1.08] | 0.94 [0.87,1.02] | 1.18 |  | 0.559 | 0.553 | 0.765 |
| Cervix uteri | 724 | 0.84 [0.58,1.16] | 1.10 [0.88,1.36] | 0.83 [0.69,0.99] | 0.86 [0.76,0.97] | 1.02 [0.95,1.10] | 1.07 |  | 0.931 | 0.873 | 0.984 |
| Brain | 663 | 0.87 [0.58,1.27] | 1.09 [0.87,1.43] | 1.03 [0.89,1.24] | 0.99 [0.88,1.10] | 1.02 [0.93,1.09] | 2.37 |  | 0.393 | 0.261 | 0.395 |
| Melanoma of skin | 618 | **1.51 [1.08,2.08]** | 1.23 [0.97,1.51] | 0.99 [0.84,1.17] | 1.03 [0.92,1.15] | 1.04 [0.96,1.13] | 2.52 |  | 0.017 | 0.081 | 0.263 |
| Esophagus | 535 | 1.35 [0.97,1.80] | 1.02 [0.77,1.31] | 0.98 [0.79,1.17] | 1.06 [0.93,1.18] | 0.97 [0.89,1.06] | 1.26 |  | 0.201 | 0.506 | 0.546 |
| Diffuse NHL^b^ | 422 | 1.00 [0.58,1.51] | 1.22 [0.92,1.61] | 0.94 [0.75,1.15] | 0.90 [0.78,1.04] | 1.03 [0.92,1.12] | 0.45 |  | 0.483 | 0.466 | 0.794 |
| Multiple myeloma | 391 | 0.89 [0.58,1.30] | 0.91 [0.61,1.25] | 1.01 [0.84,1.25] | 0.73 [0.61,0.87] | 1.02 [0.93,1.14] | 1.91 |  | 0.923 | 0.918 | 0.915 |
| Lymphoid leukaemia | 368 | 1.02 [0.62,1.57] | 0.89 [0.60,1.26] | 1.01 [0.82,1.23] | 1.01 [0.87,1.18] | 0.99 [0.90,1.10] | 6.06 |  | 0.574 | 0.593 | 0.443 |
| Myeloid leukaemia | 342 | 1.14 [0.72,1.68] | 0.84 [0.56,1.16] | 1.01 [0.80,1.27] | 1.12 [0.96,1.31] | 1.09 [0.99,1.21] | 1.15 |  | 0.257 | 0.280 | 0.085 |
| Meninges | 291 | 1.11 [0.66,1.75] | 1.24 [0.92,1.69] | 1.13 [0.89,1.42] | 1.00 [0.84,1.15] | 1.01 [0.90,1.12] | 2.15 |  | 0.130 | 0.108 | 0.269 |
| Liver | 257 | 1.33 [0.86,1.95] | 1.23 [0.88,1.70] | 1.23 [0.98,1.51] | 0.90 [0.75,1.09] | 1.00 [0.88,1.12] | 2.29 |  | 0.083 | 0.155 | 0.333 |
| Lip | 244 | 1.38 [0.90,2.11] | 0.72 [0.42,1.09] | 1.26 [1.00,1.53] | 0.88 [0.71,1.09] | 1.02 [0.91,1.14] | 0.63 |  | 0.360 | 0.640 | 0.268 |
| Hodgkin's disease | 239 | 0.87 [0.35,1.66] | 1.34 [0.91,1.91] | 1.00 [0.78,1.31] | 0.94 [0.76,1.14] | 1.04 [0.90,1.17] | 0.00 |  | 0.339 | 0.213 | 0.511 |
| Testis | 222 | 0.90 [0.31,1.83] | 1.20 [0.82,1.76] | 1.03 [0.79,1.37] | 1.05 [0.87,1.27] | **1.15 [1.01,1.32]** | 0.00 |  | 0.189 | 0.070 | 0.091 |
| Larynx | 208 | **1.73 [1.08,2.74]** | 0.79 [0.46,1.26] | 1.14 [0.86,1.44] | 0.94 [0.75,1.13] | 0.93 [0.81,1.07] | 2.11 |  | 0.312 | 0.780 | 0.610 |
| ^a^ Shown are the estimated risk ratios (RR) with 90% confidence intervals (CI) for 1st to 5th degree (1º-5º) relatives and mates of the 27 cancer sites with ≥200 cases, in bold when the 90% CI does not include 1.00, which corresponds to one-sided p-value <0.05. Also shown are combined p-values (one-sided) to evaluate the significance of the RR for all relatives (1^st^-5^th^ degree) and for close (1^st^ and 2^nd^ degree) and distant relatives (3^rd^-5^th^ degree). | | | | | | | | | | | |
| ^b^ NHL, non-Hodgkin's lymphoma. | | | | | | | | | | | |
| ^c^ na, not applicable (sex-specific cancers). | | | | | | | | | | | |
| ^d^ Number of mates or relatives with cancer/total number of mates or relatives for each cancer site | | | | | | | | | | | |

| **Table 11** RR estimates for relatives and mates of patients diagnosed with ovarian cancer for sites with 200 or more cases^a^ | | | | | | | | | | | |
| --- | --- | --- | --- | --- | --- | --- | --- | --- | --- | --- | --- |
| Cancer sites | # of aff. | RR [90% CI] | | | | | |  | combined p-value for relatives | | |
|  |  | 1º relative | 2º relatives | 3º relatives | 4º relatives | 5º relatives | Mates |  | 1º-5º | 1º-2º | 3º-5º |
| Breast | 3812 | **1.34 [1.17,1.50]** | 1.13 [1.00,1.23] | **1.10 [1.01,1.16]** | 1.06 [0.99,1.10] | 1.03 [0.99,1.05] | 0.00 |  | 0.00001 | 0.00187 | 0.00955 |
| Prostate | 3380 | **1.20 [1.05,1.35]** | 1.00 [0.88,1.10] | 1.05 [0.98,1.12] | 1.04 [0.99,1.08] | 1.05 [1.00,1.08] | 1.09 |  | 0.013 | 0.083 | 0.021 |
| Lung | 2904 | **1.18 [1.01,1.35]** | 1.05 [0.93,1.18] | 1.04 [0.96,1.12] | 0.97 [0.92,1.03] | 0.99 [0.95,1.03] | 1.23 |  | 0.114 | 0.356 | 0.529 |
| Stomach | 2890 | **1.23 [1.04,1.42]** | **1.30 [1.16,1.43]** | **1.10 [1.01,1.16]** | 1.05 [0.98,1.10] | **1.07 [1.02,1.10]** | 0.96 |  | <0.00001 | <0.00001 | 0.00267 |
| Colon | 2224 | **1.26 [1.05,1.48]** | 1.09 [0.94,1.25] | 1.02 [0.92,1.10] | 0.98 [0.91,1.04] | 0.99 [0.94,1.02] | 1.04 |  | 0.104 | 0.486 | 0.745 |
| Bladder | 1384 | 1.03 [0.83,1.30] | **1.29 [1.11,1.52]** | 1.10 [1.00,1.24] | 0.94 [0.87,1.03] | 0.97 [0.93,1.04] | 1.43 |  | 0.049 | 0.035 | 0.479 |
| Kidney | 1227 | 1.25 [1.00,1.58] | 0.93 [0.76,1.10] | 1.05 [0.92,1.16] | 1.07 [0.98,1.16] | 1.06 [0.99,1.11] | 1.19 |  | 0.073 | 0.231 | 0.064 |
| Thyroid | 957 | 1.17 [0.86,1.55] | 0.98 [0.77,1.18] | 1.07 [0.92,1.19] | 1.09 [0.98,1.17] | 1.05 [0.97,1.10] | 1.25 |  | 0.135 | 0.211 | 0.087 |
| Pancreas | 930 | **1.66 [1.31,2.05]** | 1.17 [0.98,1.42] | **1.22 [1.09,1.40]** | 1.09 [0.98,1.19] | 1.03 [0.96,1.10] | 2.17 |  | 0.00003 | 0.00197 | 0.00353 |
| Ovary | 906 | **2.01 [1.48,2.70]** | **1.62 [1.27,2.05]** | **1.24 [1.03,1.47]** | **1.18 [1.03,1.33]** | 0.92 [0.83,1.01] | na^c^ |  | <0.00001 | <0.00001 | 0.04804 |
| Non-melanoma skin | 781 | 1.18 [0.84,1.54] | **1.27 [1.01,1.57]** | 1.07 [0.90,1.23] | 0.98 [0.87,1.08] | 1.05 [0.97,1.12] | 0.88 |  | 0.045 | 0.064 | 0.269 |
| Rectum | 767 | 1.23 [0.92,1.63] | 1.14 [0.91,1.41] | 1.03 [0.88,1.18] | 1.03 [0.92,1.15] | 1.06 [0.97,1.12] | 0.46 |  | 0.044 | 0.110 | 0.209 |
| Endometrium | 753 | 1.29 [0.98,1.71] | 1.05 [0.82,1.32] | 1.10 [0.94,1.28] | 1.04 [0.94,1.15] | 0.99 [0.91,1.07] | na^c^ |  | 0.086 | 0.222 | 0.208 |
| Cervix uteri | 724 | 1.14 [0.80,1.56] | 1.04 [0.83,1.31] | 1.00 [0.84,1.17] | 1.02 [0.90,1.13] | 1.05 [0.97,1.13] | na^c^ |  | 0.237 | 0.303 | 0.328 |
| Brain | 663 | **1.47 [1.06,1.94]** | 0.81 [0.59,1.06] | 0.93 [0.78,1.10] | 0.92 [0.80,1.02] | 0.94 [0.85,1.01] | 0.00 |  | 0.727 | 0.986 | 0.968 |
| Melanoma of skin | 618 | 1.37 [0.97,2.01] | 1.11 [0.86,1.43] | 0.99 [0.83,1.17] | 0.99 [0.87,1.11] | 1.03 [0.94,1.11] | 2.38 |  | 0.082 | 0.318 | 0.482 |
| Esophagus | 535 | 1.08 [0.71,1.53] | 0.66 [0.48,0.91] | 0.94 [0.75,1.13] | 1.10 [0.97,1.23] | 1.08 [1.00,1.18] | 1.24 |  | 0.598 | 0.685 | 0.174 |
| Diffuse NHL^b^ | 422 | 0.82 [0.47,1.32] | 0.70 [0.47,0.99] | 1.16 [0.94,1.40] | 1.04 [0.90,1.19] | 1.02 [0.92,1.13] | 1.22 |  | 0.693 | 0.564 | 0.151 |
| Multiple myeloma | 391 | 0.59 [0.29,1.01] | 0.90 [0.62,1.24] | 0.81 [0.62,1.01] | 0.86 [0.72,1.01] | 0.99 [0.88,1.09] | 2.35 |  | 0.996 | 0.976 | 0.979 |
| Lymphoid leukaemia | 368 | 0.78 [0.43,1.30] | 0.86 [0.57,1.21] | 1.21 [0.98,1.48] | 0.91 [0.76,1.04] | 1.10 [0.99,1.21] | 1.24 |  | 0.583 | 0.348 | 0.143 |
| Myeloid leukaemia | 342 | 0.99 [0.57,1.56] | 0.93 [0.62,1.34] | 0.83 [0.64,1.06] | 1.02 [0.86,1.19] | 0.90 [0.79,1.00] | 0.55 |  | 0.887 | 0.908 | 0.923 |
| Meninges | 291 | 0.82 [0.44,1.45] | 1.29 [0.91,1.74] | 0.85 [0.64,1.10] | 0.98 [0.81,1.15] | **1.14 [1.01,1.27]** | 1.38 |  | 0.421 | 0.251 | 0.495 |
| Liver | 257 | 0.63 [0.31,1.16] | 1.03 [0.68,1.45] | 0.92 [0.68,1.16] | 1.00 [0.81,1.21] | 1.13 [0.99,1.26] | 1.57 |  | 0.639 | 0.404 | 0.411 |
| Lip | 244 | 0.43 [0.14,0.84] | 0.93 [0.60,1.39] | 1.05 [0.81,1.34] | 1.07 [0.88,1.26] | **1.15 [1.02,1.27]** | 0.94 |  | 0.657 | 0.208 | 0.104 |
| Hodgkin's disease | 239 | 0.88 [0.37,1.63] | 1.03 [0.67,1.54] | 1.00 [0.72,1.34] | 1.04 [0.85,1.28] | 0.85 [0.74,0.98] | 1.07 |  | 0.699 | 0.665 | 0.749 |
| Testis | 222 | 0.71 [0.28,1.68] | 1.05 [0.64,1.56] | 0.85 [0.59,1.12] | 0.77 [0.60,0.94] | 1.00 [0.87,1.17] | 1.11 |  | 0.905 | 0.900 | 0.972 |
| Larynx | 208 | 0.76 [0.35,1.38] | 1.11 [0.77,1.79] | 1.24 [0.99,1.65] | 1.03 [0.85,1.26] | 0.95 [0.82,1.08] | 1.46 |  | 0.355 | 0.163 | 0.208 |
| ^a^ Shown are the estimated risk ratios (RR) with 90% confidence intervals (CI) for 1st to 5th degree (1º-5º) relatives and mates of the 27 cancer sites with ≥200 cases, in bold when the 90% CI does not include 1.00, which corresponds to one-sided p-value <0.05. Also shown are combined p-values (one-sided) to evaluate the significance of the RR for all relatives (1^st^-5^th^ degree) and for close (1^st^ and 2^nd^ degree) and distant relatives (3^rd^-5^th^ degree). | | | | | | | | | | | |
| ^b^ NHL, non-Hodgkin's lymphoma. | | | | | | | | | | | |
| ^c^ na, not applicable (sex-specific cancers). | | | | | | | | | | | |
| ^d^ Number of mates or relatives with cancer/total number of mates or relatives for each cancer site | | | | | | | | | | | |

| **Table 12** RR estimates for relatives and mates of patients diagnosed with non-melanoma skin cancer for sites with 200 or more cases^a^ | | | | | | | | | | | |
| --- | --- | --- | --- | --- | --- | --- | --- | --- | --- | --- | --- |
| Cancer sites | # of aff. | RR [90% CI] | | | | | |  | combined p-value for relatives | | |
|  |  | 1º relative | 2º relatives | 3º relatives | 4º relatives | 5º relatives | Mates |  | 1º-5º | 1º-2º | 3º-5º |
| Breast | 3812 | 0.94 [0.79,1.08] | 1.05 [0.93,1.15] | 1.08 [0.99,1.14] | 0.99 [0.93,1.02] | 0.99 [0.95,1.01] | 1.34 |  | 0.589 | 0.386 | 0.494 |
| Prostate | 3380 | 1.06 [0.90,1.21] | 1.02 [0.89,1.12] | 0.99 [0.91,1.06] | 0.98 [0.91,1.02] | 1.02 [0.98,1.05] | 1.35 |  | 0.566 | 0.680 | 0.715 |
| Lung | 2904 | 1.11 [0.95,1.27] | 1.11 [0.97,1.24] | 1.04 [0.96,1.13] | 1.04 [0.97,1.10] | 0.99 [0.96,1.04] | 0.61 |  | 0.034 | 0.049 | 0.160 |
| Stomach | 2890 | 1.14 [0.96,1.32] | 1.13 [0.97,1.25] | 1.03 [0.94,1.11] | 1.03 [0.95,1.07] | 1.01 [0.95,1.04] | 1.48 |  | 0.059 | 0.156 | 0.366 |
| Colon | 2224 | **1.29 [1.05,1.48]** | 1.02 [0.87,1.17] | 0.98 [0.89,1.07] | 0.96 [0.89,1.01] | 1.00 [0.95,1.04] | 1.21 |  | 0.352 | 0.823 | 0.893 |
| Bladder | 1384 | 1.06 [0.83,1.32] | **1.24 [1.06,1.47]** | 1.00 [0.90,1.12] | 0.98 [0.90,1.07] | 1.00 [0.95,1.06] | 1.57 |  | 0.084 | 0.076 | 0.530 |
| Kidney | 1227 | 1.15 [0.88,1.44] | 1.04 [0.85,1.23] | 1.10 [0.98,1.24] | 1.05 [0.94,1.12] | 1.08 [1.00,1.14] | 1.63 |  | 0.041 | 0.060 | 0.035 |
| Thyroid | 957 | 0.98 [0.71,1.30] | 1.00 [0.78,1.22] | **1.17 [1.02,1.34]** | 1.09 [0.96,1.18] | 1.04 [0.95,1.09] | 1.56 |  | 0.161 | 0.089 | 0.040 |
| Pancreas | 930 | 1.29 [1.00,1.64] | 1.02 [0.80,1.30] | 1.04 [0.89,1.20] | 0.97 [0.87,1.08] | 0.98 [0.90,1.04] | 0.87 |  | 0.241 | 0.569 | 0.650 |
| Ovary | 906 | 1.18 [0.84,1.54] | **1.27 [1.01,1.57]** | 1.07 [0.90,1.23] | 0.98 [0.87,1.08] | 1.05 [0.97,1.12] | 0.88 |  | 0.045 | 0.064 | 0.269 |
| Non-melanoma skin | 781 | 1.46 [0.97,2.07] | 0.96 [0.64,1.35] | **1.44 [1.18,1.71]** | 1.04 [0.87,1.20] | 1.12 [1.00,1.23] | 2.16 |  | 0.00727 | 0.17809 | 0.00233 |
| Rectum | 767 | 1.11 [0.79,1.47] | **1.50 [1.21,1.79]** | **1.29 [1.11,1.45]** | 1.11 [0.98,1.21] | **1.12 [1.03,1.19]** | 0.82 |  | 0.00007 | 0.00002 | 0.00055 |
| Endometrium | 753 | 1.09 [0.78,1.41] | 0.81 [0.57,1.04] | 0.98 [0.81,1.13] | 1.06 [0.92,1.17] | 0.99 [0.90,1.06] | 0.52 |  | 0.747 | 0.849 | 0.574 |
| Cervix uteri | 724 | 0.82 [0.50,1.17] | 0.79 [0.55,1.06] | 1.18 [1.00,1.36] | **1.16 [1.02,1.28]** | 1.02 [0.92,1.09] | 1.18 |  | 0.514 | 0.253 | 0.020 |
| Brain | 663 | 0.99 [0.66,1.49] | 1.00 [0.70,1.26] | 1.15 [0.97,1.32] | 1.02 [0.88,1.13] | 1.03 [0.93,1.10] | 0.91 |  | 0.355 | 0.307 | 0.185 |
| Melanoma of skin | 618 | **1.73 [1.22,2.44]** | 1.21 [0.91,1.54] | 1.14 [0.93,1.35] | 1.07 [0.93,1.20] | 0.95 [0.85,1.01] | 1.11 |  | 0.009 | 0.172 | 0.329 |
| Esophagus | 535 | 1.22 [0.85,1.72] | 1.02 [0.73,1.37] | 1.16 [0.94,1.36] | 1.00 [0.86,1.12] | 1.02 [0.92,1.10] | 2.87 |  | 0.198 | 0.335 | 0.285 |
| Diffuse NHL^b^ | 422 | 0.98 [0.57,1.54] | 1.35 [0.97,1.73] | 0.90 [0.68,1.11] | 0.99 [0.83,1.14] | 1.08 [0.95,1.18] | 1.08 |  | 0.335 | 0.276 | 0.639 |
| Multiple myeloma | 391 | 0.89 [0.52,1.37] | 1.05 [0.76,1.45] | 1.01 [0.79,1.22] | 1.06 [0.89,1.21] | 1.12 [1.00,1.22] | 1.90 |  | 0.337 | 0.184 | 0.210 |
| Lymphoid leukaemia | 368 | 1.17 [0.69,1.90] | 1.05 [0.69,1.51] | 1.07 [0.85,1.32] | **1.28 [1.09,1.45]** | 1.09 [0.97,1.21] | 1.60 |  | 0.039 | 0.033 | 0.012 |
| Myeloid leukaemia | 342 | 1.28 [0.81,2.06] | 0.96 [0.61,1.33] | 0.85 [0.64,1.08] | 0.97 [0.81,1.15] | 0.91 [0.78,1.01] | 0.71 |  | 0.785 | 0.944 | 0.955 |
| Meninges | 291 | 0.99 [0.54,1.61] | 1.08 [0.71,1.53] | 0.99 [0.73,1.28] | 0.83 [0.65,1.00] | 1.10 [0.97,1.23] | 1.59 |  | 0.575 | 0.587 | 0.685 |
| Liver | 257 | 0.88 [0.44,1.50] | 0.76 [0.43,1.16] | 1.11 [0.85,1.44] | 0.95 [0.75,1.17] | 1.07 [0.94,1.20] | 0.75 |  | 0.681 | 0.623 | 0.322 |
| Lip | 244 | 1.40 [0.82,2.09] | 1.31 [0.84,1.95] | 0.99 [0.72,1.31] | 0.99 [0.80,1.21] | 1.03 [0.89,1.14] | 1.73 |  | 0.133 | 0.254 | 0.561 |
| Hodgkin's disease | 239 | 1.64 [0.91,3.00] | 1.13 [0.72,1.68] | 0.94 [0.67,1.25] | 0.80 [0.64,0.99] | 1.01 [0.87,1.15] | 0.00 |  | 0.356 | 0.740 | 0.897 |
| Testis | 222 | 0.65 [0.19,2.71] | 0.46 [0.21,0.88] | 1.25 [0.92,1.64] | 0.95 [0.75,1.17] | 0.96 [0.80,1.10] | 0.00 |  | 0.907 | 0.872 | 0.433 |
| Larynx | 208 | 1.67 [0.92,2.53] | 0.61 [0.32,1.10] | 0.76 [0.50,1.02] | 1.20 [0.96,1.43] | 1.13 [0.97,1.28] | 0.00 |  | 0.312 | 0.697 | 0.344 |
| ^a^ Shown are the estimated risk ratios (RR) with 90% confidence intervals (CI) for 1st to 5th degree (1º-5º) relatives and mates of the 27 cancer sites with ≥200 cases, in bold when the 90% CI does not include 1.00, which corresponds to one-sided p-value <0.05. Also shown are combined p-values (one-sided) to evaluate the significance of the RR for all relatives (1^st^-5^th^ degree) and for close (1^st^ and 2^nd^ degree) and distant relatives (3^rd^-5^th^ degree). | | | | | | | | | | | |
| ^b^ NHL, non-Hodgkin's lymphoma. | | | | | | | | | | | |
| ^c^ na, not applicable (sex-specific cancers). | | | | | | | | | | | |
| ^d^ Number of mates or relatives with cancer/total number of mates or relatives for each cancer site | | | | | | | | | | | |

| **Table 13** RR estimates for relatives and mates of patients diagnosed with rectal cancer for sites with 200 or more cases^a^ | | | | | | | | | | | |
| --- | --- | --- | --- | --- | --- | --- | --- | --- | --- | --- | --- |
| Cancer sites | # of aff. | RR [90% CI] | | | | | |  | combined p-value for relatives | | |
|  |  | 1º relative | 2º relatives | 3º relatives | 4º relatives | 5º relatives | Mates |  | 1º-5º | 1º-2º | 3º-5º |
| Breast | 3812 | 1.11 [0.95,1.27] | **1.20 [1.09,1.31]** | 1.04 [0.96,1.11] | 1.02 [0.96,1.06] | **1.09 [1.04,1.12]** | 1.13 |  | 0.00046 | 0.00055 | 0.01638 |
| Prostate | 3380 | 0.99 [0.84,1.15] | 1.04 [0.91,1.15] | 0.90 [0.82,0.97] | 0.95 [0.89,0.99] | 1.01 [0.97,1.05] | 1.11 |  | 0.944 | 0.961 | 0.999 |
| Lung | 2904 | 1.08 [0.93,1.25] | **1.23 [1.09,1.37]** | 0.95 [0.86,1.03] | 0.98 [0.91,1.03] | 0.98 [0.94,1.03] | 1.11 |  | 0.155 | 0.249 | 0.908 |
| Stomach | 2890 | **1.21 [1.03,1.39]** | **1.23 [1.08,1.36]** | 1.08 [0.99,1.17] | 1.04 [0.97,1.08] | 1.03 [0.98,1.06] | 1.55 |  | 0.0007 | 0.002 | 0.071 |
| Colon | 2224 | **1.64 [1.41,1.89]** | **1.27 [1.11,1.43]** | **1.13 [1.02,1.23]** | **1.10 [1.02,1.16]** | **1.06 [1.01,1.10]** | 1.50 |  | <0.00001 | 0.00001 | 0.00074 |
| Bladder | 1384 | 0.90 [0.72,1.14] | 1.08 [0.92,1.32] | 1.02 [0.91,1.15] | 0.89 [0.81,0.97] | 0.98 [0.92,1.04] | 1.41 |  | 0.750 | 0.646 | 0.868 |
| Kidney | 1227 | 1.22 [0.97,1.52] | 1.18 [0.96,1.40] | 0.85 [0.73,0.97] | 0.98 [0.88,1.07] | 1.05 [0.98,1.11] | 0.93 |  | 0.211 | 0.521 | 0.883 |
| Thyroid | 957 | 1.30 [0.99,1.70] | **1.44 [1.16,1.72]** | 0.91 [0.76,1.04] | 1.04 [0.92,1.13] | 0.99 [0.90,1.04] | 1.07 |  | 0.033 | 0.151 | 0.860 |
| Pancreas | 930 | 1.08 [0.76,1.40] | 1.18 [0.96,1.44] | 0.87 [0.73,1.01] | 1.05 [0.95,1.18] | 1.01 [0.94,1.07] | 2.18 |  | 0.289 | 0.307 | 0.658 |
| Ovary | 906 | 1.23 [0.92,1.63] | 1.14 [0.91,1.41] | 1.03 [0.88,1.18] | 1.03 [0.92,1.15] | 1.06 [0.97,1.12] | 0.46 |  | 0.044 | 0.110 | 0.209 |
| Non-melanoma skin | 781 | 1.11 [0.79,1.47] | **1.50 [1.21,1.79]** | **1.29 [1.11,1.45]** | 1.11 [0.98,1.21] | **1.12 [1.03,1.19]** | 0.82 |  | 0.00007 | 0.00002 | 0.00055 |
| Rectum | 767 | **1.68 [1.17,2.42]** | **1.63 [1.22,2.15]** | **1.31 [1.08,1.61]** | 0.90 [0.76,1.06] | 1.00 [0.90,1.12] | 1.63 |  | 0.00051 | 0.00034 | 0.15784 |
| Endometrium | 753 | 1.03 [0.75,1.44] | 0.95 [0.72,1.24] | 1.01 [0.85,1.19] | 0.97 [0.86,1.10] | 1.00 [0.93,1.08] | 0.57 |  | 0.550 | 0.628 | 0.574 |
| Cervix uteri | 724 | 0.93 [0.60,1.33] | 1.14 [0.88,1.39] | 0.87 [0.74,1.05] | 0.95 [0.84,1.07] | 1.03 [0.95,1.12] | 0.76 |  | 0.641 | 0.562 | 0.810 |
| Brain | 663 | 1.00 [0.66,1.50] | 0.76 [0.55,1.03] | 0.95 [0.78,1.17] | 1.04 [0.92,1.17] | 1.01 [0.92,1.09] | 1.42 |  | 0.708 | 0.782 | 0.472 |
| Melanoma of skin | 618 | 1.03 [0.67,1.61] | 1.03 [0.75,1.29] | **1.23 [1.05,1.47]** | 1.10 [0.96,1.25] | **1.10 [1.01,1.21]** | 1.79 |  | 0.0225 | 0.0087 | 0.0019 |
| Esophagus | 535 | **1.41 [1.05,1.94]** | 1.27 [0.94,1.63] | 0.98 [0.80,1.20] | 0.90 [0.76,1.02] | 1.09 [0.99,1.18] | 0.41 |  | 0.0387 | 0.16155 | 0.4897 |
| Diffuse NHL^b^ | 422 | 1.38 [0.93,2.07] | 0.99 [0.71,1.36] | 0.96 [0.77,1.21] | 1.07 [0.89,1.23] | 1.00 [0.91,1.11] | 2.19 |  | 0.207 | 0.452 | 0.414 |
| Multiple myeloma | 391 | 1.11 [0.70,1.65] | 1.14 [0.80,1.56] | 1.11 [0.91,1.38] | 1.03 [0.86,1.20] | 0.99 [0.89,1.10] | 1.92 |  | 0.163 | 0.170 | 0.240 |
| Lymphoid leukaemia | 368 | 0.88 [0.47,1.51] | 1.07 [0.70,1.48] | 0.92 [0.69,1.16] | 1.04 [0.89,1.22] | 0.93 [0.84,1.04] | 0.00 |  | 0.672 | 0.578 | 0.706 |
| Myeloid leukaemia | 342 | 1.50 [0.97,2.33] | 1.01 [0.70,1.47] | 0.74 [0.56,0.95] | 0.96 [0.81,1.14] | 1.01 [0.91,1.13] | 1.43 |  | 0.410 | 0.817 | 0.890 |
| Meninges | 291 | 1.57 [0.99,2.56] | 1.14 [0.79,1.63] | 0.93 [0.70,1.24] | 1.03 [0.87,1.25] | 0.93 [0.80,1.07] | 1.69 |  | 0.162 | 0.476 | 0.656 |
| Liver | 257 | 1.44 [0.84,2.36] | **1.67 [1.19,2.39]** | 1.11 [0.87,1.48] | 1.05 [0.85,1.26] | 0.94 [0.83,1.08] | 0.75 |  | 0.009 | 0.026 | 0.296 |
| Lip | 244 | 0.86 [0.42,1.60] | 1.26 [0.78,1.83] | 1.16 [0.92,1.51] | 0.76 [0.59,0.96] | **1.17 [1.06,1.33]** | 0.83 |  | 0.299 | 0.172 | 0.252 |
| Hodgkin's disease | 239 | 1.03 [0.45,2.13] | 0.91 [0.57,1.39] | 1.09 [0.79,1.46] | 1.15 [0.94,1.39] | 1.07 [0.92,1.21] | 3.52 |  | 0.296 | 0.249 | 0.129 |
| Testis | 222 | 1.03 [0.37,3.45] | 1.01 [0.64,1.58] | 0.95 [0.65,1.26] | 1.00 [0.78,1.25] | 1.04 [0.90,1.21] | 0.00 |  | 0.507 | 0.512 | 0.545 |
| Larynx | 208 | 1.13 [0.56,2.11] | 1.04 [0.57,1.50] | 1.01 [0.75,1.35] | 0.80 [0.61,0.99] | 1.00 [0.88,1.17] | 1.69 |  | 0.644 | 0.740 | 0.787 |
| ^a^ Shown are the estimated risk ratios (RR) with 90% confidence intervals (CI) for 1st to 5th degree (1º-5º) relatives and mates of the 27 cancer sites with ≥200 cases, in bold when the 90% CI does not include 1.00, which corresponds to one-sided p-value <0.05. Also shown are combined p-values (one-sided) to evaluate the significance of the RR for all relatives (1^st^-5^th^ degree) and for close (1^st^ and 2^nd^ degree) and distant relatives (3^rd^-5^th^ degree). | | | | | | | | | | | |
| ^b^ NHL, non-Hodgkin's lymphoma. | | | | | | | | | | | |
| ^c^ na, not applicable (sex-specific cancers). | | | | | | | | | | | |
| ^d^ Number of mates or relatives with cancer/total number of mates or relatives for each cancer site | | | | | | | | | | | |

| **Table 14** RR estimates for relatives and mates of patients diagnosed with endometrial cancer for sites with 200 or more cases^a^ | | | | | | | | | | | |
| --- | --- | --- | --- | --- | --- | --- | --- | --- | --- | --- | --- |
| Cancer sites | # of aff. | RR [90% CI] | | | | | |  | combined p-value for relatives | | |
|  |  | 1º relative | 2º relatives | 3º relatives | 4º relatives | 5º relatives | Mates |  | 1º-5º | 1º-2º | 3º-5º |
| Breast | 3812 | 1.05 [0.90,1.18] | 1.14 [1.00,1.24] | 1.01 [0.93,1.07] | 0.95 [0.88,0.98] | 1.05 [1.00,1.08] | 2.68 |  | 0.230 | 0.270 | 0.710 |
| Prostate | 3380 | 1.06 [0.91,1.23] | **1.18 [1.03,1.29]** | 1.07 [0.98,1.14] | 0.99 [0.92,1.03] | 1.00 [0.95,1.03] | 1.15 |  | 0.063 | 0.078 | 0.467 |
| Lung | 2904 | 0.90 [0.75,1.06] | 1.03 [0.89,1.16] | 0.94 [0.86,1.02] | 1.02 [0.96,1.08] | 0.98 [0.94,1.03] | 1.13 |  | 0.821 | 0.639 | 0.776 |
| Stomach | 2890 | **1.51 [1.30,1.73]** | **1.41 [1.25,1.54]** | 1.07 [0.98,1.15] | **1.09 [1.02,1.15]** | 1.00 [0.95,1.03] | 0.97 |  | <0.00001 | <0.00001 | 0.04577 |
| Colon | 2224 | **1.33 [1.11,1.54]** | 1.12 [0.95,1.26] | 1.07 [0.97,1.17] | 0.97 [0.89,1.04] | 1.02 [0.96,1.06] | 1.75 |  | 0.010 | 0.200 | 0.404 |
| Bladder | 1384 | 1.09 [0.86,1.39] | 0.95 [0.77,1.15] | 0.97 [0.85,1.10] | 1.09 [1.00,1.19] | **1.07 [1.01,1.14]** | 1.12 |  | 0.136 | 0.159 | 0.049 |
| Kidney | 1227 | **1.47 [1.14,1.77]** | 1.13 [0.93,1.39] | 1.09 [0.95,1.21] | 1.10 [1.00,1.20] | 1.04 [0.96,1.09] | 0.67 |  | 0.0018 | 0.0378 | 0.0619 |
| Thyroid | 957 | **1.45 [1.12,1.84]** | 1.28 [1.00,1.54] | 1.08 [0.89,1.22] | 1.00 [0.88,1.09] | 1.01 [0.92,1.06] | 0.95 |  | 0.026 | 0.168 | 0.540 |
| Pancreas | 930 | 0.99 [0.75,1.34] | 1.10 [0.88,1.36] | 1.04 [0.89,1.19] | 0.96 [0.86,1.08] | 0.94 [0.87,1.02] | 1.18 |  | 0.559 | 0.553 | 0.765 |
| Ovary | 906 | 1.29 [0.98,1.71] | 1.05 [0.82,1.32] | 1.10 [0.94,1.28] | 1.04 [0.94,1.15] | 0.99 [0.91,1.07] | na^c^ |  | 0.086 | 0.222 | 0.208 |
| Non-melanoma skin | 781 | 1.09 [0.78,1.41] | 0.81 [0.57,1.04] | 0.98 [0.81,1.13] | 1.06 [0.92,1.17] | 0.99 [0.90,1.06] | 0.52 |  | 0.747 | 0.849 | 0.574 |
| Rectum | 767 | 1.03 [0.75,1.44] | 0.95 [0.72,1.24] | 1.01 [0.85,1.19] | 0.97 [0.86,1.10] | 1.00 [0.93,1.08] | 0.57 |  | 0.550 | 0.628 | 0.574 |
| Endometrium | 753 | **1.86 [1.31,2.62]** | 1.26 [0.90,1.74] | **1.57 [1.30,1.89]** | 1.05 [0.89,1.24] | 1.12 [1.00,1.24] | na^c^ |  | <0.00001 | 0.00298 | 0.00018 |
| Cervix uteri | 724 | 0.96 [0.66,1.41] | 1.09 [0.82,1.39] | **1.18 [1.02,1.38]** | 0.96 [0.83,1.08] | 0.96 [0.87,1.03] | na^c^ |  | 0.371 | 0.294 | 0.373 |
| Brain | 663 | **1.41 [1.02,2.01]** | 1.18 [0.91,1.51] | 1.02 [0.85,1.24] | 1.01 [0.89,1.15] | 1.03 [0.93,1.10] | 2.11 |  | 0.042 | 0.184 | 0.364 |
| Melanoma of skin | 618 | 0.99 [0.67,1.53] | 0.97 [0.71,1.29] | 0.78 [0.61,0.95] | 1.00 [0.88,1.12] | 1.05 [0.96,1.14] | 0.66 |  | 0.778 | 0.844 | 0.844 |
| Esophagus | 535 | 0.71 [0.46,1.14] | 0.90 [0.65,1.24] | 1.15 [0.96,1.39] | 1.08 [0.93,1.24] | 0.88 [0.79,0.97] | 1.59 |  | 0.743 | 0.526 | 0.391 |
| Diffuse NHL^b^ | 422 | 1.34 [0.85,1.90] | 0.77 [0.52,1.17] | 1.17 [0.94,1.44] | 1.08 [0.92,1.27] | 1.13 [1.00,1.25] | 2.58 |  | 0.068 | 0.161 | 0.022 |
| Multiple myeloma | 391 | **1.72 [1.22,2.49]** | 1.01 [0.66,1.38] | **1.34 [1.10,1.64]** | 1.08 [0.90,1.26] | 0.97 [0.87,1.08] | 1.21 |  | 0.0077 | 0.1025 | 0.0485 |
| Lymphoid leukaemia | 368 | 1.23 [0.77,2.00] | 0.73 [0.48,1.07] | 1.12 [0.89,1.41] | 0.96 [0.79,1.14] | 0.88 [0.78,0.99] | 0.65 |  | 0.727 | 0.919 | 0.727 |
| Myeloid leukaemia | 342 | 1.49 [0.94,2.34] | 1.00 [0.68,1.42] | 1.11 [0.89,1.41] | 1.01 [0.84,1.20] | 1.09 [0.97,1.21] | 0.59 |  | 0.056 | 0.195 | 0.149 |
| Meninges | 291 | 1.24 [0.75,2.11] | 1.24 [0.85,1.75] | 0.95 [0.74,1.26] | 0.97 [0.79,1.15] | 0.98 [0.84,1.09] | 0.00 |  | 0.291 | 0.434 | 0.719 |
| Liver | 257 | 0.79 [0.39,1.53] | 0.53 [0.29,0.92] | 1.02 [0.76,1.36] | 0.90 [0.73,1.09] | 0.81 [0.70,0.93] | 0.54 |  | 0.989 | 0.992 | 0.936 |
| Lip | 244 | 1.03 [0.61,1.80] | 0.52 [0.27,0.90] | 0.84 [0.57,1.18] | 0.99 [0.78,1.20] | 1.07 [0.92,1.20] | 1.46 |  | 0.862 | 0.914 | 0.656 |
| Hodgkin's disease | 239 | 1.38 [0.70,2.69] | 0.88 [0.52,1.37] | 1.28 [0.95,1.66] | 1.10 [0.89,1.35] | 0.98 [0.83,1.12] | 2.46 |  | 0.153 | 0.234 | 0.114 |
| Testis | 222 | 0.56 [0.12,2.53] | 0.77 [0.47,1.29] | 1.04 [0.77,1.46] | 1.13 [0.90,1.37] | 1.06 [0.90,1.22] | 0.00 |  | 0.613 | 0.381 | 0.179 |
| Larynx | 208 | 1.26 [0.76,2.30] | 0.65 [0.32,1.16] | 1.14 [0.84,1.48] | 0.93 [0.74,1.15] | 0.85 [0.72,0.99] | 0.00 |  | 0.751 | 0.927 | 0.762 |
| ^a^ Shown are the estimated risk ratios (RR) with 90% confidence intervals (CI) for 1st to 5th degree (1º-5º) relatives and mates of the 27 cancer sites with ≥200 cases, in bold when the 90% CI does not include 1.00, which corresponds to one-sided p-value <0.05. Also shown are combined p-values (one-sided) to evaluate the significance of the RR for all relatives (1^st^-5^th^ degree) and for close (1^st^ and 2^nd^ degree) and distant relatives (3^rd^-5^th^ degree). | | | | | | | | | | | |
| ^b^ NHL, non-Hodgkin's lymphoma. | | | | | | | | | | | |
| ^c^ na, not applicable (sex-specific cancers). | | | | | | | | | | | |
| ^d^ Number of mates or relatives with cancer/total number of mates or relatives for each cancer site | | | | | | | | | | | |

| **Table 15** RR estimates for relatives and mates of patients diagnosed with cervical cancer for sites with 200 or more cases^a^ | | | | | | | | | | | |
| --- | --- | --- | --- | --- | --- | --- | --- | --- | --- | --- | --- |
| Cancer sites | # of aff. | RR [90% CI] | | | | | |  | combined p-value for relatives | | |
|  |  | 1º relative | 2º relatives | 3º relatives | 4º relatives | 5º relatives | Mates |  | 1º-5º | 1º-2º | 3º-5º |
| Breast | 3812 | 1.03 [0.88,1.21] | 0.97 [0.84,1.07] | 1.05 [0.96,1.11] | 0.97 [0.90,1.01] | 0.98 [0.93,1.00] | 0.00 |  | 0.814 | 0.904 | 0.865 |
| Prostate | 3380 | 1.03 [0.86,1.21] | 1.10 [0.96,1.21] | 0.98 [0.89,1.05] | 1.04 [0.97,1.09] | 1.03 [0.98,1.06] | 0.93 |  | 0.191 | 0.172 | 0.336 |
| Lung | 2904 | **1.31 [1.11,1.52]** | **1.32 [1.14,1.46]** | **1.17 [1.07,1.26]** | **1.10 [1.02,1.15]** | 1.04 [0.99,1.08] | 2.01 |  | <0.00001 | <0.00001 | 0.00008 |
| Stomach | 2890 | **1.52 [1.30,1.77]** | **1.14 [1.01,1.28]** | 1.09 [0.98,1.17] | 1.02 [0.95,1.08] | 1.05 [1.00,1.09] | 1.02 |  | 0.00002 | 0.01322 | 0.04785 |
| Colon | 2224 | 1.14 [0.91,1.39] | 0.94 [0.80,1.09] | 0.95 [0.84,1.04] | 1.02 [0.93,1.07] | 1.00 [0.95,1.04] | 1.75 |  | 0.649 | 0.864 | 0.805 |
| Bladder | 1384 | **1.32 [1.05,1.67]** | 1.00 [0.84,1.24] | 0.91 [0.79,1.03] | 0.95 [0.87,1.04] | 1.00 [0.94,1.07] | 1.98 |  | 0.304 | 0.790 | 0.911 |
| Kidney | 1227 | 0.88 [0.66,1.22] | 1.09 [0.89,1.33] | **1.18 [1.03,1.33]** | 0.97 [0.87,1.06] | 1.04 [0.96,1.10] | 1.89 |  | 0.222 | 0.092 | 0.123 |
| Thyroid | 957 | 1.12 [0.83,1.56] | 1.22 [0.97,1.50] | 1.15 [0.97,1.32] | 1.08 [0.94,1.17] | 1.02 [0.93,1.07] | 0.41 |  | 0.036 | 0.048 | 0.113 |
| Pancreas | 930 | 0.84 [0.58,1.16] | 1.10 [0.88,1.36] | 0.83 [0.69,0.99] | 0.86 [0.76,0.97] | 1.02 [0.95,1.10] | 1.07 |  | 0.931 | 0.873 | 0.984 |
| Ovary | 906 | 1.14 [0.80,1.56] | 1.04 [0.83,1.31] | 1.00 [0.84,1.17] | 1.02 [0.90,1.13] | 1.05 [0.97,1.13] | na^c^ |  | 0.237 | 0.303 | 0.328 |
| Non-melanoma skin | 781 | 0.82 [0.50,1.17] | 0.79 [0.55,1.06] | 1.18 [1.00,1.36] | **1.16 [1.02,1.28]** | 1.02 [0.92,1.09] | 1.18 |  | 0.514 | 0.253 | 0.020 |
| Rectum | 767 | 0.93 [0.60,1.33] | 1.14 [0.88,1.39] | 0.87 [0.74,1.05] | 0.95 [0.84,1.07] | 1.03 [0.95,1.12] | 0.76 |  | 0.641 | 0.562 | 0.810 |
| Endometrium | 753 | 0.96 [0.66,1.41] | 1.09 [0.82,1.39] | **1.18 [1.02,1.38]** | 0.96 [0.83,1.08] | 0.96 [0.87,1.03] | na^c^ |  | 0.371 | 0.294 | 0.373 |
| Cervix uteri | 724 | **1.74 [1.12,2.73]** | **1.71 [1.24,2.26]** | 1.10 [0.86,1.40] | 1.16 [0.98,1.36] | 0.99 [0.88,1.12] | na^c^ |  | 0.00053 | 0.00079 | 0.11505 |
| Brain | 663 | 0.47 [0.27,0.89] | **1.42 [1.11,1.77]** | **1.32 [1.11,1.54]** | 0.91 [0.79,1.04] | 0.97 [0.88,1.06] | 1.98 |  | 0.287 | 0.024 | 0.213 |
| Melanoma of skin | 618 | 1.03 [0.67,1.66] | 0.87 [0.63,1.19] | 1.18 [0.98,1.41] | 1.01 [0.87,1.15] | 0.93 [0.84,1.02] | 2.99 |  | 0.491 | 0.576 | 0.348 |
| Esophagus | 535 | **1.74 [1.23,2.40]** | 1.19 [0.90,1.53] | **1.39 [1.15,1.65]** | **1.24 [1.09,1.40]** | 1.06 [0.97,1.17] | 3.02 |  | <0.00001 | 0.00014 | 0.00004 |
| Diffuse NHL^b^ | 422 | 0.80 [0.42,1.35] | 0.88 [0.58,1.28] | 1.14 [0.90,1.42] | 0.99 [0.84,1.17] | 0.93 [0.83,1.05] | 1.06 |  | 0.751 | 0.591 | 0.423 |
| Multiple myeloma | 391 | 1.14 [0.74,1.79] | 0.95 [0.62,1.32] | 0.86 [0.67,1.12] | 1.15 [0.98,1.36] | **1.12 [1.01,1.25]** | 1.04 |  | 0.195 | 0.250 | 0.132 |
| Lymphoid leukaemia | 368 | 0.80 [0.36,1.47] | 1.07 [0.72,1.52] | 0.99 [0.74,1.24] | 1.02 [0.85,1.20] | 0.95 [0.84,1.07] | 1.66 |  | 0.653 | 0.563 | 0.620 |
| Myeloid leukaemia | 342 | 1.29 [0.71,2.10] | 1.17 [0.75,1.63] | 1.08 [0.84,1.38] | 0.92 [0.75,1.10] | 0.95 [0.83,1.07] | 0.72 |  | 0.322 | 0.515 | 0.667 |
| Meninges | 291 | 1.19 [0.71,1.98] | 1.15 [0.78,1.64] | 0.99 [0.78,1.32] | 1.12 [0.95,1.36] | 1.01 [0.90,1.17] | 1.79 |  | 0.090 | 0.119 | 0.180 |
| Liver | 257 | **1.81 [1.12,2.97]** | 1.08 [0.69,1.74] | 1.13 [0.86,1.49] | 0.80 [0.63,0.99] | 1.06 [0.93,1.22] | 0.00 |  | 0.077 | 0.400 | 0.514 |
| Lip | 244 | 0.58 [0.24,1.27] | 1.08 [0.68,1.61] | 1.18 [0.86,1.55] | 1.06 [0.85,1.29] | 0.89 [0.75,1.03] | 0.66 |  | 0.602 | 0.354 | 0.382 |
| Hodgkin's disease | 239 | 1.57 [0.83,2.89] | 0.53 [0.28,0.94] | 1.25 [0.88,1.62] | **1.25 [1.03,1.56]** | 1.12 [0.97,1.28] | 1.37 |  | 0.078 | 0.198 | 0.010 |
| Testis | 222 | 0.57 [0.14,1.36] | 1.29 [0.83,2.00] | 1.01 [0.69,1.41] | 0.91 [0.70,1.15] | 1.10 [0.93,1.26] | 1.18 |  | 0.528 | 0.289 | 0.500 |
| Larynx | 208 | **2.19 [1.37,3.82]** | 1.31 [0.82,2.01] | 1.13 [0.81,1.53] | 0.95 [0.74,1.18] | 1.15 [0.99,1.32] | 1.95 |  | 0.0053 | 0.08925 | 0.1668 |
| ^a^ Shown are the estimated risk ratios (RR) with 90% confidence intervals (CI) for 1st to 5th degree (1º-5º) relatives and mates of the 27 cancer sites with ≥200 cases, in bold when the 90% CI does not include 1.00, which corresponds to one-sided p-value <0.05. Also shown are combined p-values (one-sided) to evaluate the significance of the RR for all relatives (1^st^-5^th^ degree) and for close (1^st^ and 2^nd^ degree) and distant relatives (3^rd^-5^th^ degree). | | | | | | | | | | | |
| ^b^ NHL, non-Hodgkin's lymphoma. | | | | | | | | | | | |
| ^c^ na, not applicable (sex-specific cancers). | | | | | | | | | | | |
| ^d^ Number of mates or relatives with cancer/total number of mates or relatives for each cancer site | | | | | | | | | | | |

| **Table 16** RR estimates for relatives and mates of patients diagnosed with brain cancer for sites with 200 or more cases^a^ | | | | | | | | | | | |
| --- | --- | --- | --- | --- | --- | --- | --- | --- | --- | --- | --- |
| Cancer sites | # of aff. | RR [90% CI] | | | | | |  | combined p-value for relatives | | |
|  |  | 1º relative | 2º relatives | 3º relatives | 4º relatives | 5º relatives | Mates |  | 1º-5º | 1º-2º | 3º-5º |
| Breast | 3812 | 1.00 [0.83,1.17] | 1.03 [0.91,1.16] | 1.07 [0.98,1.14] | 1.01 [0.94,1.06] | 1.00 [0.95,1.02] | 1.64 |  | 0.388 | 0.342 | 0.392 |
| Prostate | 3380 | 1.08 [0.91,1.28] | 1.09 [0.96,1.22] | **1.15 [1.05,1.23]** | 0.94 [0.88,0.99] | 1.03 [0.98,1.06] | 1.08 |  | 0.074 | 0.090 | 0.175 |
| Lung | 2904 | 1.13 [0.92,1.33] | 0.92 [0.78,1.05] | 0.99 [0.90,1.08] | **1.08 [1.01,1.15]** | 0.98 [0.95,1.03] | 1.17 |  | 0.327 | 0.553 | 0.218 |
| Stomach | 2890 | **1.30 [1.06,1.54]** | 1.10 [0.94,1.25] | **1.19 [1.07,1.29]** | **1.11 [1.04,1.16]** | 1.04 [0.99,1.08] | 1.02 |  | 0.00001 | 0.00019 | 0.00006 |
| Colon | 2224 | 0.94 [0.72,1.21] | 0.92 [0.79,1.10] | 1.03 [0.92,1.14] | 0.99 [0.92,1.07] | 0.99 [0.94,1.04] | 0.96 |  | 0.765 | 0.711 | 0.536 |
| Bladder | 1384 | **1.43 [1.15,1.88]** | 0.96 [0.78,1.21] | 0.95 [0.81,1.09] | 1.07 [0.97,1.17] | **1.08 [1.02,1.15]** | 0.00 |  | 0.016 | 0.213 | 0.105 |
| Kidney | 1227 | **1.41 [1.08,1.84]** | **1.25 [1.02,1.49]** | **1.23 [1.07,1.38]** | 1.06 [0.95,1.14] | 1.06 [0.98,1.12] | 0.30 |  | 0.00029 | 0.00232 | 0.00986 |
| Thyroid | 957 | **1.51 [1.09,2.03]** | 0.90 [0.69,1.17] | 0.98 [0.82,1.13] | **1.23 [1.07,1.34]** | 1.10 [0.99,1.16] | 1.22 |  | 0.019 | 0.119 | 0.029 |
| Pancreas | 930 | 0.87 [0.58,1.27] | 1.09 [0.87,1.43] | 1.03 [0.89,1.24] | 0.99 [0.88,1.10] | 1.02 [0.93,1.09] | 2.37 |  | 0.393 | 0.261 | 0.395 |
| Ovary | 906 | **1.47 [1.06,1.94]** | 0.81 [0.59,1.06] | 0.93 [0.78,1.10] | 0.92 [0.80,1.02] | 0.94 [0.85,1.01] | 0.00 |  | 0.727 | 0.986 | 0.968 |
| Non-melanoma skin | 781 | 0.99 [0.66,1.49] | 1.00 [0.70,1.26] | 1.15 [0.97,1.32] | 1.02 [0.88,1.13] | 1.03 [0.93,1.10] | 0.91 |  | 0.355 | 0.307 | 0.185 |
| Rectum | 767 | 1.00 [0.66,1.50] | 0.76 [0.55,1.03] | 0.95 [0.78,1.17] | 1.04 [0.92,1.17] | 1.01 [0.92,1.09] | 1.42 |  | 0.708 | 0.782 | 0.472 |
| Endometrium | 753 | **1.41 [1.02,2.01]** | 1.18 [0.91,1.51] | 1.02 [0.85,1.24] | 1.01 [0.89,1.15] | 1.03 [0.93,1.10] | 2.11 |  | 0.042 | 0.184 | 0.364 |
| Cervix uteri | 724 | 0.47 [0.27,0.89] | **1.42 [1.11,1.77]** | **1.32 [1.11,1.54]** | 0.91 [0.79,1.04] | 0.97 [0.88,1.06] | 1.98 |  | 0.287 | 0.024 | 0.213 |
| Brain | 663 | 1.41 [0.74,2.40] | 1.10 [0.69,1.62] | 0.83 [0.59,1.10] | **1.31 [1.09,1.54]** | 0.99 [0.85,1.11] | 1.22 |  | 0.16029 | 0.18892 | 0.30278 |
| Melanoma of skin | 618 | **1.79 [1.22,2.60]** | 1.15 [0.82,1.55] | 1.01 [0.79,1.21] | 0.95 [0.80,1.09] | 1.00 [0.89,1.08] | 1.31 |  | 0.064 | 0.502 | 0.728 |
| Esophagus | 535 | 1.12 [0.67,1.77] | 0.90 [0.64,1.25] | 1.00 [0.80,1.22] | 0.99 [0.83,1.14] | 0.93 [0.83,1.02] | 1.44 |  | 0.718 | 0.849 | 0.808 |
| Diffuse NHL^b^ | 422 | 1.27 [0.69,1.96] | 1.20 [0.83,1.64] | 0.97 [0.72,1.19] | 0.85 [0.70,1.01] | 0.93 [0.80,1.03] | 0.00 |  | 0.651 | 0.828 | 0.953 |
| Multiple myeloma | 391 | 1.33 [0.77,2.18] | 0.92 [0.57,1.41] | 1.11 [0.86,1.37] | 1.04 [0.86,1.22] | 1.03 [0.90,1.14] | 1.50 |  | 0.286 | 0.437 | 0.295 |
| Lymphoid leukaemia | 368 | 1.68 [0.92,2.80] | 0.97 [0.59,1.39] | 0.90 [0.65,1.15] | 1.20 [1.00,1.40] | 0.98 [0.85,1.10] | 0.00 |  | 0.159 | 0.477 | 0.417 |
| Myeloid leukaemia | 342 | 0.14 [0.01,0.57] | 0.97 [0.60,1.48] | 0.98 [0.71,1.25] | 0.90 [0.72,1.11] | 1.02 [0.88,1.14] | 2.12 |  | 0.966 | 0.735 | 0.730 |
| Meninges | 291 | 1.55 [0.86,2.60] | 0.63 [0.34,1.06] | 0.96 [0.69,1.27] | 1.10 [0.88,1.29] | 0.98 [0.84,1.11] | 2.20 |  | 0.440 | 0.782 | 0.487 |
| Liver | 257 | 1.20 [0.55,2.04] | 0.95 [0.53,1.62] | 1.16 [0.88,1.50] | 0.95 [0.73,1.15] | **1.21 [1.05,1.37]** | 2.46 |  | 0.222 | 0.211 | 0.113 |
| Lip | 244 | 0.87 [0.31,1.98] | 0.78 [0.42,1.37] | 1.20 [0.85,1.54] | 1.13 [0.92,1.36] | 0.90 [0.75,1.03] | 3.12 |  | 0.648 | 0.563 | 0.351 |
| Hodgkin's disease | 239 | 1.03 [0.45,2.07] | 1.25 [0.81,2.04] | 0.64 [0.41,0.95] | 0.94 [0.71,1.16] | 1.09 [0.91,1.25] | 0.00 |  | 0.541 | 0.601 | 0.878 |
| Testis | 222 | 1.31 [0.59,2.33] | 0.98 [0.55,1.66] | 1.18 [0.82,1.64] | 0.85 [0.63,1.06] | 1.10 [0.91,1.25] | 3.16 |  | 0.379 | 0.506 | 0.472 |
| Larynx | 208 | 1.00 [0.42,2.08] | 0.20 [0.03,0.54] | 1.31 [0.94,1.69] | 0.96 [0.73,1.20] | 1.05 [0.89,1.20] | 2.47 |  | 0.799 | 0.830 | 0.200 |
| ^a^ Shown are the estimated risk ratios (RR) with 90% confidence intervals (CI) for 1st to 5th degree (1º-5º) relatives and mates of the 27 cancer sites with ≥200 cases, in bold when the 90% CI does not include 1.00, which corresponds to one-sided p-value <0.05. Also shown are combined p-values (one-sided) to evaluate the significance of the RR for all relatives (1^st^-5^th^ degree) and for close (1^st^ and 2^nd^ degree) and distant relatives (3^rd^-5^th^ degree). | | | | | | | | | | | |
| ^b^ NHL, non-Hodgkin's lymphoma. | | | | | | | | | | | |
| ^c^ na, not applicable (sex-specific cancers). | | | | | | | | | | | |
| ^d^ Number of mates or relatives with cancer/total number of mates or relatives for each cancer site | | | | | | | | | | | |

| **Table 17** RR estimates for relatives and mates of patients diagnosed with melanoma for sites with 200 or more cases^a^ | | | | | | | | | | | |
| --- | --- | --- | --- | --- | --- | --- | --- | --- | --- | --- | --- |
| Cancer sites | # of aff. | RR [90% CI] | | | | | |  | combined p-value for relatives | | |
|  |  | 1º relative | 2º relatives | 3º relatives | 4º relatives | 5º relatives | Mates |  | 1º-5º | 1º-2º | 3º-5º |
| Breast | 3812 | 0.80 [0.65,0.98] | **1.22 [1.09,1.34]** | **1.11 [1.01,1.19]** | 1.02 [0.95,1.07] | **1.07 [1.02,1.10]** | 1.42 |  | 0.0523 | 0.0004 | 0.0095 |
| Prostate | 3380 | **1.49 [1.25,1.74]** | **1.14 [1.01,1.28]** | **1.15 [1.06,1.24]** | 1.06 [0.99,1.11] | 1.01 [0.96,1.04] | 1.16 |  | <0.00001 | 0.00286 | 0.0112 |
| Lung | 2904 | 1.07 [0.87,1.31] | **1.29 [1.12,1.43]** | 1.00 [0.91,1.09] | 0.98 [0.92,1.05] | 1.01 [0.97,1.06] | 0.39 |  | 0.034 | 0.032 | 0.468 |
| Stomach | 2890 | 0.90 [0.69,1.13] | 1.06 [0.92,1.20] | **1.15 [1.05,1.25]** | 1.06 [0.99,1.13] | **1.07 [1.01,1.10]** | 0.34 |  | 0.0559 | 0.0044 | 0.0012 |
| Colon | 2224 | 1.07 [0.83,1.35] | 1.11 [0.94,1.27] | 1.08 [0.96,1.18] | 1.03 [0.94,1.10] | 1.00 [0.94,1.04] | 1.45 |  | 0.132 | 0.141 | 0.272 |
| Bladder | 1384 | 1.02 [0.75,1.38] | 1.05 [0.86,1.30] | 1.10 [0.98,1.25] | 1.01 [0.92,1.10] | 0.97 [0.91,1.04] | 0.56 |  | 0.255 | 0.235 | 0.268 |
| Kidney | 1227 | **1.52 [1.10,2.00]** | **1.31 [1.04,1.58]** | **1.29 [1.12,1.45]** | 1.06 [0.95,1.15] | **1.10 [1.01,1.17]** | 1.72 |  | 0.00001 | 0.00008 | 0.00071 |
| Thyroid | 957 | **1.62 [1.17,2.24]** | 0.73 [0.52,0.95] | 1.15 [0.97,1.32] | 1.05 [0.92,1.15] | 0.95 [0.86,1.02] | 1.01 |  | 0.212 | 0.788 | 0.344 |
| Pancreas | 930 | **1.51 [1.08,2.08]** | 1.23 [0.97,1.51] | 0.99 [0.84,1.17] | 1.03 [0.92,1.15] | 1.04 [0.96,1.13] | 2.52 |  | 0.017 | 0.081 | 0.263 |
| Ovary | 906 | 1.37 [0.97,2.01] | 1.11 [0.86,1.43] | 0.99 [0.83,1.17] | 0.99 [0.87,1.11] | 1.03 [0.94,1.11] | 2.38 |  | 0.082 | 0.318 | 0.482 |
| Non-melanoma skin | 781 | **1.73 [1.22,2.44]** | 1.21 [0.91,1.54] | 1.14 [0.93,1.35] | 1.07 [0.93,1.20] | 0.95 [0.85,1.01] | 1.11 |  | 0.009 | 0.172 | 0.329 |
| Rectum | 767 | 1.03 [0.67,1.61] | 1.03 [0.75,1.29] | **1.23 [1.05,1.47]** | 1.10 [0.96,1.25] | **1.10 [1.01,1.21]** | 1.79 |  | 0.0225 | 0.0087 | 0.0019 |
| Endometrium | 753 | 0.99 [0.67,1.53] | 0.97 [0.71,1.29] | 0.78 [0.61,0.95] | 1.00 [0.88,1.12] | 1.05 [0.96,1.14] | 0.66 |  | 0.778 | 0.844 | 0.844 |
| Cervix uteri | 724 | 1.03 [0.67,1.66] | 0.87 [0.63,1.19] | 1.18 [0.98,1.41] | 1.01 [0.87,1.15] | 0.93 [0.84,1.02] | 2.99 |  | 0.491 | 0.576 | 0.348 |
| Brain | 663 | **1.79 [1.22,2.60]** | 1.15 [0.82,1.55] | 1.01 [0.79,1.21] | 0.95 [0.80,1.09] | 1.00 [0.89,1.08] | 1.31 |  | 0.064 | 0.502 | 0.728 |
| Melanoma of skin | 618 | **1.86 [1.06,3.35]** | **1.61 [1.08,2.31]** | 1.23 [0.90,1.58] | 1.00 [0.78,1.19] | 0.92 [0.77,1.04] | 1.52 |  | 0.02082 | 0.00606 | 0.52545 |
| Esophagus | 535 | 1.04 [0.59,1.57] | 1.03 [0.68,1.37] | 1.15 [0.91,1.39] | **1.22 [1.02,1.35]** | 1.02 [0.89,1.11] | 0.82 |  | 0.178 | 0.138 | 0.068 |
| Diffuse NHL^b^ | 422 | 0.96 [0.49,1.74] | 1.33 [0.92,1.81] | 0.80 [0.56,1.03] | 1.12 [0.91,1.30] | 1.08 [0.93,1.18] | 0.88 |  | 0.325 | 0.277 | 0.553 |
| Multiple myeloma | 391 | 1.31 [0.73,2.08] | 1.13 [0.71,1.59] | 0.99 [0.70,1.24] | 1.13 [0.93,1.31] | 1.08 [0.93,1.18] | 0.00 |  | 0.175 | 0.237 | 0.316 |
| Lymphoid leukaemia | 368 | 1.41 [0.84,2.34] | **1.50 [1.01,2.11]** | 1.11 [0.83,1.40] | 1.14 [0.95,1.31] | **1.18 [1.01,1.29]** | 0.00 |  | 0.01127 | 0.01159 | 0.06038 |
| Myeloid leukaemia | 342 | **1.73 [1.02,2.78]** | 0.86 [0.53,1.30] | 1.03 [0.73,1.31] | 0.74 [0.56,0.90] | 1.00 [0.83,1.10] | 0.00 |  | 0.625 | 0.973 | 0.967 |
| Meninges | 291 | 1.39 [0.68,2.28] | 1.19 [0.76,1.75] | 1.22 [0.88,1.56] | 1.08 [0.87,1.30] | 1.15 [0.98,1.27] | 1.43 |  | 0.047 | 0.078 | 0.095 |
| Liver | 257 | 1.58 [0.78,2.94] | 1.11 [0.65,1.65] | 1.19 [0.82,1.53] | 1.10 [0.86,1.34] | 0.94 [0.77,1.07] | 1.40 |  | 0.171 | 0.386 | 0.413 |
| Lip | 244 | 1.25 [0.58,2.43] | 1.02 [0.63,1.61] | 1.23 [0.92,1.63] | 1.15 [0.89,1.37] | 1.09 [0.91,1.22] | 1.56 |  | 0.126 | 0.138 | 0.087 |
| Hodgkin's disease | 239 | 0.29 [0.01,1.89] | 0.68 [0.34,1.26] | 1.09 [0.74,1.48] | 1.23 [0.97,1.45] | 1.04 [0.87,1.18] | 0.00 |  | 0.824 | 0.492 | 0.193 |
| Testis | 222 | 0.34 [0.01,2.03] | 0.76 [0.35,1.47] | 0.84 [0.55,1.26] | 1.03 [0.77,1.28] | 1.02 [0.82,1.17] | 1.86 |  | 0.937 | 0.818 | 0.729 |
| Larynx | 208 | 0.91 [0.41,1.92] | 1.14 [0.65,1.89] | 1.16 [0.81,1.61] | 0.89 [0.64,1.09] | 1.00 [0.81,1.13] | 0.00 |  | 0.544 | 0.518 | 0.659 |
| ^a^ Shown are the estimated risk ratios (RR) with 90% confidence intervals (CI) for 1st to 5th degree (1º-5º) relatives and mates of the 27 cancer sites with ≥200 cases, in bold when the 90% CI does not include 1.00, which corresponds to one-sided p-value <0.05. Also shown are combined p-values (one-sided) to evaluate the significance of the RR for all relatives (1^st^-5^th^ degree) and for close (1^st^ and 2^nd^ degree) and distant relatives (3^rd^-5^th^ degree). | | | | | | | | | | | |
| ^b^ NHL, non-Hodgkin's lymphoma. | | | | | | | | | | | |
| ^c^ na, not applicable (sex-specific cancers). | | | | | | | | | | | |
| ^d^ Number of mates or relatives with cancer/total number of mates or relatives for each cancer site | | | | | | | | | | | |

| **Table 18** RR estimates for relatives and mates of patients diagnosed with esophageal cancer for sites with 200 or more cases^a^ | | | | | | | | | | | |
| --- | --- | --- | --- | --- | --- | --- | --- | --- | --- | --- | --- |
| Cancer sites | # of aff. | RR [90% CI] | | | | | |  | combined p-value for relatives | | |
|  |  | 1º relative | 2º relatives | 3º relatives | 4º relatives | 5º relatives | Mates |  | 1º-5º | 1º-2º | 3º-5º |
| Breast | 3812 | 1.03 [0.84,1.21] | 1.05 [0.92,1.18] | 0.99 [0.90,1.07] | 0.92 [0.86,0.96] | 1.04 [0.99,1.07] | 1.49 |  | 0.703 | 0.739 | 0.903 |
| Prostate | 3380 | **1.20 [1.02,1.40]** | 0.89 [0.76,1.02] | 1.04 [0.93,1.13] | 1.02 [0.95,1.08] | 1.05 [1.00,1.09] | 1.68 |  | 0.159 | 0.453 | 0.114 |
| Lung | 2904 | **1.36 [1.13,1.60]** | 1.11 [0.96,1.29] | 1.00 [0.89,1.10] | 1.05 [0.99,1.14] | 1.01 [0.96,1.06] | 1.71 |  | 0.00351 | 0.08273 | 0.18707 |
| Stomach | 2890 | **1.31 [1.08,1.54]** | 1.16 [0.99,1.34] | 1.13 [1.00,1.21] | **1.11 [1.02,1.17]** | **1.06 [1.01,1.10]** | 1.37 |  | 0.00003 | 0.00043 | 0.00104 |
| Colon | 2224 | 1.01 [0.80,1.26] | 0.87 [0.71,1.02] | 0.99 [0.88,1.11] | 1.05 [0.95,1.11] | 0.97 [0.90,1.01] | 1.37 |  | 0.838 | 0.895 | 0.693 |
| Bladder | 1384 | 1.17 [0.90,1.51] | 0.91 [0.74,1.15] | 0.98 [0.83,1.13] | 1.05 [0.96,1.16] | 1.01 [0.95,1.08] | 1.31 |  | 0.247 | 0.484 | 0.315 |
| Kidney | 1227 | 1.07 [0.76,1.44] | 1.18 [0.90,1.42] | **1.18 [1.01,1.34]** | 0.97 [0.86,1.07] | 1.04 [0.95,1.10] | 1.14 |  | 0.106 | 0.107 | 0.161 |
| Thyroid | 957 | 0.91 [0.56,1.34] | 1.21 [0.93,1.52] | 0.90 [0.73,1.05] | 0.96 [0.82,1.06] | 0.99 [0.89,1.05] | 0.78 |  | 0.755 | 0.711 | 0.945 |
| Pancreas | 930 | 1.35 [0.97,1.80] | 1.02 [0.77,1.31] | 0.98 [0.79,1.17] | 1.06 [0.93,1.18] | 0.97 [0.89,1.06] | 1.26 |  | 0.201 | 0.506 | 0.546 |
| Ovary | 906 | 1.08 [0.71,1.53] | 0.66 [0.48,0.91] | 0.94 [0.75,1.13] | 1.10 [0.97,1.23] | 1.08 [1.00,1.18] | 1.24 |  | 0.598 | 0.685 | 0.174 |
| Non-melanoma skin | 781 | 1.22 [0.85,1.72] | 1.02 [0.73,1.37] | 1.16 [0.94,1.36] | 1.00 [0.86,1.12] | 1.02 [0.92,1.10] | 2.87 |  | 0.198 | 0.335 | 0.285 |
| Rectum | 767 | **1.41 [1.05,1.94]** | 1.27 [0.94,1.63] | 0.98 [0.80,1.20] | 0.90 [0.76,1.02] | 1.09 [0.99,1.18] | 0.41 |  | 0.0387 | 0.16155 | 0.4897 |
| Endometrium | 753 | 0.71 [0.46,1.14] | 0.90 [0.65,1.24] | 1.15 [0.96,1.39] | 1.08 [0.93,1.24] | 0.88 [0.79,0.97] | 1.59 |  | 0.743 | 0.526 | 0.391 |
| Cervix uteri | 724 | **1.74 [1.23,2.40]** | 1.19 [0.90,1.53] | **1.39 [1.15,1.65]** | **1.24 [1.09,1.40]** | 1.06 [0.97,1.17] | 3.02 |  | <0.00001 | 0.00014 | 0.00004 |
| Brain | 663 | 1.12 [0.67,1.77] | 0.90 [0.64,1.25] | 1.00 [0.80,1.22] | 0.99 [0.83,1.14] | 0.93 [0.83,1.02] | 1.44 |  | 0.718 | 0.849 | 0.808 |
| Melanoma of skin | 618 | 1.04 [0.59,1.57] | 1.03 [0.68,1.37] | 1.15 [0.91,1.39] | **1.22 [1.02,1.35]** | 1.02 [0.89,1.11] | 0.82 |  | 0.178 | 0.138 | 0.068 |
| Esophagus | 535 | **2.09 [1.30,3.31]** | **1.62 [1.07,2.38]** | 1.04 [0.74,1.41] | 1.14 [0.91,1.40] | 1.07 [0.92,1.23] | 2.40 |  | 0.0015 | 0.00134 | 0.16738 |
| Diffuse NHL^b^ | 422 | 1.52 [0.86,2.43] | 0.99 [0.66,1.41] | **1.30 [1.02,1.64]** | 1.05 [0.84,1.22] | 1.05 [0.91,1.17] | 0.95 |  | 0.060 | 0.132 | 0.065 |
| Multiple myeloma | 391 | **1.54 [1.01,2.33]** | 1.06 [0.68,1.58] | 1.10 [0.85,1.39] | 1.00 [0.80,1.18] | 1.02 [0.90,1.15] | 0.77 |  | 0.101 | 0.357 | 0.381 |
| Lymphoid leukaemia | 368 | 1.29 [0.73,2.31] | 1.26 [0.84,1.97] | 1.23 [0.93,1.56] | 0.92 [0.75,1.12] | 1.00 [0.87,1.11] | 0.00 |  | 0.129 | 0.162 | 0.328 |
| Myeloid leukaemia | 342 | **2.12 [1.28,3.31]** | 1.05 [0.64,1.68] | 1.19 [0.85,1.54] | 0.94 [0.77,1.16] | **1.18 [1.02,1.34]** | 1.17 |  | 0.006 | 0.178 | 0.142 |
| Meninges | 291 | 0.84 [0.35,1.59] | 1.05 [0.62,1.62] | 1.00 [0.66,1.30] | **1.32 [1.09,1.60]** | 1.04 [0.88,1.19] | 0.00 |  | 0.250 | 0.143 | 0.099 |
| Liver | 257 | **1.93 [1.24,3.08]** | 0.78 [0.42,1.41] | 1.17 [0.81,1.53] | 1.02 [0.81,1.23] | 0.88 [0.74,1.02] | 1.19 |  | 0.221 | 0.724 | 0.557 |
| Lip | 244 | 1.69 [1.00,2.81] | 1.10 [0.66,1.66] | 0.57 [0.35,0.86] | 1.11 [0.87,1.38] | 1.06 [0.91,1.21] | 1.37 |  | 0.287 | 0.707 | 0.804 |
| Hodgkin's disease | 239 | 1.64 [0.69,4.50] | 0.60 [0.24,1.11] | 0.80 [0.49,1.13] | 1.09 [0.85,1.36] | 0.77 [0.62,0.90] | 0.00 |  | 0.815 | 0.979 | 0.951 |
| Testis | 222 | **2.37 [1.04,5.66]** | 0.93 [0.45,1.68] | 1.08 [0.74,1.59] | 1.19 [0.90,1.46] | 1.10 [0.92,1.30] | 0.00 |  | 0.050 | 0.220 | 0.125 |
| Larynx | 208 | 1.46 [0.66,2.75] | 0.59 [0.26,1.25] | 1.04 [0.71,1.48] | 1.10 [0.85,1.38] | 1.02 [0.84,1.20] | 1.58 |  | 0.435 | 0.656 | 0.313 |
| ^a^ Shown are the estimated risk ratios (RR) with 90% confidence intervals (CI) for 1st to 5th degree (1º-5º) relatives and mates of the 27 cancer sites with ≥200 cases, in bold when the 90% CI does not include 1.00, which corresponds to one-sided p-value <0.05. Also shown are combined p-values (one-sided) to evaluate the significance of the RR for all relatives (1^st^-5^th^ degree) and for close (1^st^ and 2^nd^ degree) and distant relatives (3^rd^-5^th^ degree). | | | | | | | | | | | |
| ^b^ NHL, non-Hodgkin's lymphoma. | | | | | | | | | | | |
| ^c^ na, not applicable (sex-specific cancers). | | | | | | | | | | | |
| ^d^ Number of mates or relatives with cancer/total number of mates or relatives for each cancer site | | | | | | | | | | | |

| **Table 19** RR estimates for relatives and mates of patients diagnosed with diffuse NHL^b^ for sites with 200 or more cases^a^ | | | | | | | | | | | |
| --- | --- | --- | --- | --- | --- | --- | --- | --- | --- | --- | --- |
| Cancer sites | # of aff. | RR [90% CI] | | | | | |  | combined p-value for relatives | | |
|  |  | 1º relative | 2º relatives | 3º relatives | 4º relatives | 5º relatives | Mates |  | 1º-5º | 1º-2º | 3º-5º |
| Breast | 3812 | 1.05 [0.84,1.27] | 0.87 [0.72,1.00] | 0.91 [0.80,1.00] | 1.07 [0.99,1.13] | 1.06 [1.00,1.09] | 1.67 |  | 0.690 | 0.798 | 0.393 |
| Prostate | 3380 | 1.15 [0.95,1.40] | 1.10 [0.92,1.29] | 1.06 [0.94,1.14] | 1.05 [0.96,1.12] | 1.03 [0.98,1.07] | 0.66 |  | 0.024 | 0.054 | 0.109 |
| Lung | 2904 | 1.04 [0.83,1.29] | 0.99 [0.81,1.15] | 0.98 [0.88,1.09] | 1.04 [0.96,1.12] | 0.99 [0.94,1.04] | 1.26 |  | 0.480 | 0.561 | 0.477 |
| Stomach | 2890 | 0.96 [0.73,1.22] | 1.06 [0.89,1.23] | 0.99 [0.86,1.09] | 0.98 [0.89,1.06] | 1.04 [0.97,1.08] | 0.70 |  | 0.579 | 0.493 | 0.643 |
| Colon | 2224 | 1.10 [0.83,1.40] | 1.01 [0.82,1.23] | 1.10 [0.97,1.23] | 1.06 [0.95,1.14] | 1.05 [0.98,1.11] | 1.49 |  | 0.101 | 0.104 | 0.049 |
| Bladder | 1384 | 1.04 [0.72,1.46] | 0.91 [0.67,1.21] | **1.22 [1.06,1.42]** | 0.95 [0.83,1.06] | 1.03 [0.96,1.12] | 0.92 |  | 0.258 | 0.241 | 0.086 |
| Kidney | 1227 | **1.37 [1.02,1.93]** | **1.33 [1.01,1.66]** | 1.16 [0.96,1.34] | 1.14 [1.00,1.26] | 1.10 [1.00,1.18] | 0.73 |  | 0.00051 | 0.00099 | 0.00573 |
| Thyroid | 957 | 1.17 [0.75,1.72] | 0.67 [0.44,0.93] | 1.06 [0.86,1.27] | 1.10 [0.95,1.25] | 0.90 [0.79,0.97] | 2.44 |  | 0.808 | 0.933 | 0.600 |
| Pancreas | 930 | 1.00 [0.58,1.51] | 1.22 [0.92,1.61] | 0.94 [0.75,1.15] | 0.90 [0.78,1.04] | 1.03 [0.92,1.12] | 0.45 |  | 0.483 | 0.466 | 0.794 |
| Ovary | 906 | 0.82 [0.47,1.32] | 0.70 [0.47,0.99] | 1.16 [0.94,1.40] | 1.04 [0.90,1.19] | 1.02 [0.92,1.13] | 1.22 |  | 0.693 | 0.564 | 0.151 |
| Non-melanoma skin | 781 | 0.98 [0.57,1.54] | 1.35 [0.97,1.73] | 0.90 [0.68,1.11] | 0.99 [0.83,1.14] | 1.08 [0.95,1.18] | 1.08 |  | 0.335 | 0.276 | 0.639 |
| Rectum | 767 | 1.38 [0.93,2.07] | 0.99 [0.71,1.36] | 0.96 [0.77,1.21] | 1.07 [0.89,1.23] | 1.00 [0.91,1.11] | 2.19 |  | 0.207 | 0.452 | 0.414 |
| Endometrium | 753 | 1.34 [0.85,1.90] | 0.77 [0.52,1.17] | 1.17 [0.94,1.44] | 1.08 [0.92,1.27] | 1.13 [1.00,1.25] | 2.58 |  | 0.068 | 0.161 | 0.022 |
| Cervix uteri | 724 | 0.80 [0.42,1.35] | 0.88 [0.58,1.28] | 1.14 [0.90,1.42] | 0.99 [0.84,1.17] | 0.93 [0.83,1.05] | 1.06 |  | 0.751 | 0.591 | 0.423 |
| Brain | 663 | 1.27 [0.69,1.96] | 1.20 [0.83,1.64] | 0.97 [0.72,1.19] | 0.85 [0.70,1.01] | 0.93 [0.80,1.03] | 0.00 |  | 0.651 | 0.828 | 0.953 |
| Melanoma of skin | 618 | 0.96 [0.49,1.74] | 1.33 [0.92,1.81] | 0.80 [0.56,1.03] | 1.12 [0.91,1.30] | 1.08 [0.93,1.18] | 0.88 |  | 0.325 | 0.277 | 0.553 |
| Esophagus | 535 | 1.52 [0.86,2.43] | 0.99 [0.66,1.41] | **1.30 [1.02,1.64]** | 1.05 [0.84,1.22] | 1.05 [0.91,1.17] | 0.95 |  | 0.060 | 0.132 | 0.065 |
| Diffuse NHL^b^ | 422 | 1.46 [0.65,4.20] | 1.19 [0.61,2.11] | 1.42 [0.98,2.02] | 0.96 [0.70,1.30] | 0.92 [0.74,1.13] | 2.07 |  | 0.12743 | 0.17125 | 0.24982 |
| Multiple myeloma | 391 | 0.82 [0.38,1.65] | 1.14 [0.70,1.70] | 1.00 [0.73,1.37] | 0.91 [0.72,1.14] | 1.00 [0.85,1.15] | 2.89 |  | 0.563 | 0.468 | 0.599 |
| Lymphoid leukaemia | 368 | **1.94 [1.16,3.41]** | 1.30 [0.84,2.01] | 0.65 [0.44,0.96] | 1.06 [0.87,1.30] | 1.09 [0.93,1.27] | 1.67 |  | 0.072 | 0.356 | 0.647 |
| Myeloid leukaemia | 342 | 1.90 [1.00,3.36] | 1.08 [0.61,1.81] | **1.37 [1.01,1.81]** | 1.20 [0.95,1.48] | 0.91 [0.78,1.07] | 1.39 |  | 0.022 | 0.091 | 0.070 |
| Meninges | 291 | 1.48 [0.62,2.73] | 0.37 [0.12,0.89] | 1.07 [0.73,1.48] | 1.07 [0.85,1.38] | 1.06 [0.89,1.24] | 1.38 |  | 0.505 | 0.741 | 0.227 |
| Liver | 257 | 1.13 [0.55,2.22] | 1.12 [0.59,1.94] | 0.89 [0.61,1.34] | 1.07 [0.83,1.38] | 1.00 [0.83,1.21] | 3.34 |  | 0.364 | 0.418 | 0.482 |
| Lip | 244 | 0.91 [0.41,1.84] | 1.31 [0.76,2.39] | 1.33 [0.96,1.83] | 0.76 [0.56,1.00] | 0.97 [0.80,1.16] | 2.04 |  | 0.337 | 0.275 | 0.509 |
| Hodgkin's disease | 239 | **2.56 [1.40,5.95]** | 0.78 [0.38,1.42] | 1.15 [0.78,1.65] | 0.89 [0.66,1.19] | 0.94 [0.76,1.14] | 0.00 |  | 0.084 | 0.658 | 0.519 |
| Testis | 222 | 2.06 [0.97,5.17] | 1.20 [0.72,2.17] | 1.04 [0.66,1.65] | 1.02 [0.74,1.34] | 1.01 [0.82,1.24] | 0.00 |  | 0.059 | 0.261 | 0.385 |
| Larynx | 208 | 0/2008^d^ | 0.49 [0.15,1.14] | 0.96 [0.62,1.47] | 0.90 [0.62,1.18] | 1.04 [0.87,1.28] | 0.00 |  | 0.981 | 0.845 | 0.583 |
| ^a^ Shown are the estimated risk ratios (RR) with 90% confidence intervals (CI) for 1st to 5th degree (1º-5º) relatives and mates of the 27 cancer sites with ≥200 cases, in bold when the 90% CI does not include 1.00, which corresponds to one-sided p-value <0.05. Also shown are combined p-values (one-sided) to evaluate the significance of the RR for all relatives (1^st^-5^th^ degree) and for close (1^st^ and 2^nd^ degree) and distant relatives (3^rd^-5^th^ degree). | | | | | | | | | | | |
| ^b^ NHL, non-Hodgkin's lymphoma. | | | | | | | | | | | |
| ^c^ na, not applicable (sex-specific cancers). | | | | | | | | | | | |
| ^d^ Number of mates or relatives with cancer/total number of mates or relatives for each cancer site | | | | | | | | | | | |

| **Table 20** RR estimates for relatives and mates of patients diagnosed with multiple myeloma for sites with 200 or more cases^a^ | | | | | | | | | | | |
| --- | --- | --- | --- | --- | --- | --- | --- | --- | --- | --- | --- |
| Cancer sites | # of aff. | RR [90% CI] | | | | | |  | combined p-value for relatives | | |
|  |  | 1º relative | 2º relatives | 3º relatives | 4º relatives | 5º relatives | Mates |  | 1º-5º | 1º-2º | 3º-5º |
| Breast | 3812 | 1.10 [0.86,1.30] | **1.35 [1.18,1.53]** | 1.02 [0.91,1.11] | 0.98 [0.89,1.04] | 1.03 [0.97,1.07] | 0.73 |  | 0.02093 | 0.02072 | 0.56336 |
| Prostate | 3380 | **1.30 [1.09,1.53]** | 1.15 [0.99,1.33] | 1.09 [0.98,1.17] | 1.05 [0.96,1.12] | 1.04 [0.98,1.08] | 1.26 |  | 0.0014 | 0.016 | 0.042 |
| Lung | 2904 | 1.11 [0.87,1.36] | 1.14 [0.95,1.33] | 1.04 [0.93,1.16] | 1.01 [0.92,1.09] | 0.93 [0.88,0.98] | 1.19 |  | 0.231 | 0.351 | 0.667 |
| Stomach | 2890 | **1.33 [1.10,1.62]** | 1.14 [0.95,1.31] | 1.03 [0.91,1.14] | 1.07 [0.98,1.14] | 0.97 [0.91,1.01] | 1.93 |  | 0.026 | 0.218 | 0.424 |
| Colon | 2224 | 0.99 [0.75,1.28] | 0.93 [0.75,1.15] | 1.10 [0.97,1.24] | 0.93 [0.83,1.00] | 0.92 [0.86,0.97] | 0.66 |  | 0.895 | 0.908 | 0.900 |
| Bladder | 1384 | 0.75 [0.50,1.06] | 0.95 [0.69,1.27] | 1.16 [0.99,1.35] | 1.02 [0.90,1.15] | 0.95 [0.88,1.04] | 0.00 |  | 0.643 | 0.343 | 0.215 |
| Kidney | 1227 | **1.71 [1.29,2.24]** | 1.17 [0.88,1.48] | 0.95 [0.77,1.12] | 1.14 [0.99,1.26] | 1.07 [0.96,1.14] | 1.29 |  | 0.0038 | 0.117 | 0.1863 |
| Thyroid | 957 | **1.65 [1.17,2.36]** | 1.21 [0.90,1.60] | 1.18 [0.97,1.40] | 0.82 [0.67,0.94] | 1.00 [0.89,1.08] | 0.47 |  | 0.082 | 0.445 | 0.750 |
| Pancreas | 930 | 0.89 [0.58,1.30] | 0.91 [0.61,1.25] | 1.01 [0.84,1.25] | 0.73 [0.61,0.87] | 1.02 [0.93,1.14] | 1.91 |  | 0.923 | 0.918 | 0.915 |
| Ovary | 906 | 0.59 [0.29,1.01] | 0.90 [0.62,1.24] | 0.81 [0.62,1.01] | 0.86 [0.72,1.01] | 0.99 [0.88,1.09] | 2.35 |  | 0.996 | 0.976 | 0.979 |
| Non-melanoma skin | 781 | 0.89 [0.52,1.37] | 1.05 [0.76,1.45] | 1.01 [0.79,1.22] | 1.06 [0.89,1.21] | 1.12 [1.00,1.22] | 1.90 |  | 0.337 | 0.184 | 0.210 |
| Rectum | 767 | 1.11 [0.70,1.65] | 1.14 [0.80,1.56] | 1.11 [0.91,1.38] | 1.03 [0.86,1.20] | 0.99 [0.89,1.10] | 1.92 |  | 0.163 | 0.170 | 0.240 |
| Endometrium | 753 | **1.72 [1.22,2.49]** | 1.01 [0.66,1.38] | **1.34 [1.10,1.64]** | 1.08 [0.90,1.26] | 0.97 [0.87,1.08] | 1.21 |  | 0.0077 | 0.1025 | 0.0485 |
| Cervix uteri | 724 | 1.14 [0.74,1.79] | 0.95 [0.62,1.32] | 0.86 [0.67,1.12] | 1.15 [0.98,1.36] | **1.12 [1.01,1.25]** | 1.04 |  | 0.195 | 0.250 | 0.132 |
| Brain | 663 | 1.33 [0.77,2.18] | 0.92 [0.57,1.41] | 1.11 [0.86,1.37] | 1.04 [0.86,1.22] | 1.03 [0.90,1.14] | 1.50 |  | 0.286 | 0.437 | 0.295 |
| Melanoma of skin | 618 | 1.31 [0.73,2.08] | 1.13 [0.71,1.59] | 0.99 [0.70,1.24] | 1.13 [0.93,1.31] | 1.08 [0.93,1.18] | 0.00 |  | 0.175 | 0.237 | 0.316 |
| Esophagus | 535 | **1.54 [1.01,2.33]** | 1.06 [0.68,1.58] | 1.10 [0.85,1.39] | 1.00 [0.80,1.18] | 1.02 [0.90,1.15] | 0.77 |  | 0.101 | 0.357 | 0.381 |
| Diffuse NHL^b^ | 422 | 0.82 [0.38,1.65] | 1.14 [0.70,1.70] | 1.00 [0.73,1.37] | 0.91 [0.72,1.14] | 1.00 [0.85,1.15] | 2.89 |  | 0.563 | 0.468 | 0.599 |
| Multiple myeloma | 391 | **2.66 [1.54,6.10]** | 1.32 [0.69,2.62] | 0.57 [0.29,0.99] | 1.15 [0.85,1.55] | 1.10 [0.89,1.34] | 3.65 |  | 0.03144 | 0.00743 | 0.58679 |
| Lymphoid leukaemia | 368 | 1.31 [0.64,2.59] | 1.41 [0.90,2.22] | 1.02 [0.73,1.42] | 0.95 [0.74,1.20] | 0.88 [0.74,1.01] | 1.49 |  | 0.249 | 0.383 | 0.756 |
| Myeloid leukaemia | 342 | 1.15 [0.52,2.36] | 0.93 [0.54,1.58] | 0.89 [0.62,1.24] | 0.81 [0.60,1.01] | 1.01 [0.86,1.17] | 1.36 |  | 0.750 | 0.858 | 0.889 |
| Meninges | 291 | 0.43 [0.07,1.17] | 1.27 [0.74,2.17] | **1.55 [1.11,2.06]** | 0.77 [0.54,0.97] | 1.06 [0.90,1.25] | 1.52 |  | 0.439 | 0.140 | 0.223 |
| Liver | 257 | 1.29 [0.69,2.47] | 1.63 [0.96,2.53] | 1.01 [0.63,1.46] | 0.90 [0.67,1.20] | **1.22 [1.03,1.45]** | 0.00 |  | 0.045 | 0.056 | 0.278 |
| Lip | 244 | 1.49 [0.74,2.75] | 1.08 [0.59,2.03] | 0.70 [0.45,1.05] | 0.74 [0.51,0.99] | 0.96 [0.77,1.15] | 3.32 |  | 0.748 | 0.920 | 0.979 |
| Hodgkin's disease | 239 | 0.78 [0.20,2.98] | **2.58 [1.78,3.66]** | 0.97 [0.63,1.48] | 1.12 [0.84,1.43] | 1.05 [0.87,1.29] | 0.00 |  | 0.0228 | 0.0027 | 0.2974 |
| Testis | 222 | 1.24 [0.29,3.81] | 1.51 [0.87,2.58] | 0.95 [0.61,1.52] | 1.31 [0.97,1.66] | 1.06 [0.86,1.29] | 0.00 |  | 0.154 | 0.079 | 0.160 |
| Larynx | 208 | 0.82 [0.29,3.04] | 0.52 [0.16,1.47] | 0.66 [0.42,1.10] | 0.95 [0.68,1.30] | 0.85 [0.68,1.06] | 1.61 |  | 0.955 | 0.969 | 0.940 |
| ^a^ Shown are the estimated risk ratios (RR) with 90% confidence intervals (CI) for 1st to 5th degree (1º-5º) relatives and mates of the 27 cancer sites with ≥200 cases, in bold when the 90% CI does not include 1.00, which corresponds to one-sided p-value <0.05. Also shown are combined p-values (one-sided) to evaluate the significance of the RR for all relatives (1^st^-5^th^ degree) and for close (1^st^ and 2^nd^ degree) and distant relatives (3^rd^-5^th^ degree). | | | | | | | | | | | |
| ^b^ NHL, non-Hodgkin's lymphoma. | | | | | | | | | | | |
| ^c^ na, not applicable (sex-specific cancers). | | | | | | | | | | | |
| ^d^ Number of mates or relatives with cancer/total number of mates or relatives for each cancer site | | | | | | | | | | | |

| **Table 21** RR estimates for relatives and mates of patients diagnosed with lymphoid leukaemia for sites with 200 or more cases^a^ | | | | | | | | | | | |
| --- | --- | --- | --- | --- | --- | --- | --- | --- | --- | --- | --- |
| Cancer sites | # of aff. | RR [90% CI] | | | | | |  | combined p-value for relatives | | |
|  |  | 1º relative | 2º relatives | 3º relatives | 4º relatives | 5º relatives | Mates |  | 1º-5º | 1º-2º | 3º-5º |
| Breast | 3812 | 0.92 [0.69,1.17] | 1.08 [0.90,1.23] | 1.07 [0.96,1.17] | 1.08 [0.99,1.14] | 1.01 [0.95,1.05] | 0.58 |  | 0.228 | 0.081 | 0.093 |
| Prostate | 3380 | 1.02 [0.79,1.30] | 0.97 [0.82,1.15] | 1.02 [0.90,1.11] | 0.99 [0.91,1.06] | 1.02 [0.95,1.06] | 1.83 |  | 0.585 | 0.631 | 0.558 |
| Lung | 2904 | 0.63 [0.42,0.88] | 1.14 [0.95,1.33] | 1.09 [0.98,1.22] | 1.02 [0.94,1.10] | 0.98 [0.93,1.04] | 1.23 |  | 0.644 | 0.085 | 0.203 |
| Stomach | 2890 | 1.26 [0.99,1.58] | 0.93 [0.74,1.12] | **1.20 [1.07,1.33]** | 1.06 [0.96,1.15] | 1.03 [0.96,1.07] | 0.42 |  | 0.029 | 0.104 | 0.010 |
| Colon | 2224 | 1.01 [0.71,1.33] | 0.69 [0.51,0.86] | 0.95 [0.81,1.09] | 0.97 [0.87,1.06] | 1.00 [0.93,1.06] | 1.05 |  | 0.973 | 0.993 | 0.829 |
| Bladder | 1384 | 1.03 [0.68,1.48] | **1.27 [1.01,1.63]** | 1.18 [1.00,1.38] | 0.96 [0.84,1.09] | 1.06 [0.98,1.15] | 1.41 |  | 0.045 | 0.022 | 0.089 |
| Kidney | 1227 | 1.33 [0.87,1.84] | 0.90 [0.66,1.21] | 1.08 [0.89,1.28] | 1.13 [0.98,1.26] | 1.06 [0.97,1.15] | 1.03 |  | 0.076 | 0.203 | 0.050 |
| Thyroid | 957 | **1.57 [1.02,2.27]** | 0.92 [0.62,1.24] | 0.93 [0.73,1.10] | 0.98 [0.81,1.10] | 1.01 [0.89,1.09] | 2.12 |  | 0.390 | 0.859 | 0.833 |
| Pancreas | 930 | 1.02 [0.62,1.57] | 0.89 [0.60,1.26] | 1.01 [0.82,1.23] | 1.01 [0.87,1.18] | 0.99 [0.90,1.10] | 6.06 |  | 0.574 | 0.593 | 0.443 |
| Ovary | 906 | 0.78 [0.43,1.30] | 0.86 [0.57,1.21] | 1.21 [0.98,1.48] | 0.91 [0.76,1.04] | 1.10 [0.99,1.21] | 1.24 |  | 0.583 | 0.348 | 0.143 |
| Non-melanoma skin | 781 | 1.17 [0.69,1.90] | 1.05 [0.69,1.51] | 1.07 [0.85,1.32] | **1.28 [1.09,1.45]** | 1.09 [0.97,1.21] | 1.60 |  | 0.039 | 0.033 | 0.012 |
| Rectum | 767 | 0.88 [0.47,1.51] | 1.07 [0.70,1.48] | 0.92 [0.69,1.16] | 1.04 [0.89,1.22] | 0.93 [0.84,1.04] | 0.00 |  | 0.672 | 0.578 | 0.706 |
| Endometrium | 753 | 1.23 [0.77,2.00] | 0.73 [0.48,1.07] | 1.12 [0.89,1.41] | 0.96 [0.79,1.14] | 0.88 [0.78,0.99] | 0.65 |  | 0.727 | 0.919 | 0.727 |
| Cervix uteri | 724 | 0.80 [0.36,1.47] | 1.07 [0.72,1.52] | 0.99 [0.74,1.24] | 1.02 [0.85,1.20] | 0.95 [0.84,1.07] | 1.66 |  | 0.653 | 0.563 | 0.620 |
| Brain | 663 | 1.68 [0.92,2.80] | 0.97 [0.59,1.39] | 0.90 [0.65,1.15] | 1.20 [1.00,1.40] | 0.98 [0.85,1.10] | 0.00 |  | 0.159 | 0.477 | 0.417 |
| Melanoma of skin | 618 | 1.41 [0.84,2.34] | **1.50 [1.01,2.11]** | 1.11 [0.83,1.40] | 1.14 [0.95,1.31] | **1.18 [1.01,1.29]** | 0.00 |  | 0.01127 | 0.01159 | 0.06038 |
| Esophagus | 535 | 1.29 [0.73,2.31] | 1.26 [0.84,1.97] | 1.23 [0.93,1.56] | 0.92 [0.75,1.12] | 1.00 [0.87,1.11] | 0.00 |  | 0.129 | 0.162 | 0.328 |
| Diffuse NHL^b^ | 422 | **1.94 [1.16,3.41]** | 1.30 [0.84,2.01] | 0.65 [0.44,0.96] | 1.06 [0.87,1.30] | 1.09 [0.93,1.27] | 1.67 |  | 0.072 | 0.356 | 0.647 |
| Multiple myeloma | 391 | 1.31 [0.64,2.59] | 1.41 [0.90,2.22] | 1.02 [0.73,1.42] | 0.95 [0.74,1.20] | 0.88 [0.74,1.01] | 1.49 |  | 0.249 | 0.383 | 0.756 |
| Lymphoid leukaemia | 368 | **4.56 [2.52,8.86]** | 1.53 [0.74,2.80] | **1.57 [1.01,2.31]** | 1.25 [0.89,1.67] | 1.06 [0.82,1.29] | 0.00 |  | 0.00004 | 0.00024 | 0.03474 |
| Myeloid leukaemia | 342 | 1.32 [0.54,3.96] | 1.06 [0.58,1.84] | 1.01 [0.65,1.44] | 1.16 [0.86,1.46] | 1.15 [0.96,1.31] | 2.00 |  | 0.186 | 0.234 | 0.193 |
| Meninges | 291 | **2.32 [1.13,5.59]** | 1.09 [0.61,1.84] | 0.59 [0.33,0.96] | 1.18 [0.86,1.46] | 0.92 [0.74,1.10] | 0.00 |  | 0.239 | 0.799 | 0.887 |
| Liver | 257 | 0.92 [0.30,3.23] | 1.53 [0.88,2.54] | 0.99 [0.61,1.48] | 0.98 [0.71,1.31] | 1.09 [0.89,1.27] | 0.00 |  | 0.252 | 0.211 | 0.500 |
| Lip | 244 | 1.49 [0.69,4.25] | 1.56 [0.91,2.57] | **1.51 [1.05,2.08]** | 1.14 [0.85,1.45] | 0.96 [0.77,1.16] | 0.00 |  | 0.024 | 0.037 | 0.089 |
| Hodgkin's disease | 239 | 1.55 [0.54,4.35] | 0.77 [0.35,1.48] | **1.98 [1.47,2.72]** | 1.13 [0.80,1.45] | 0.83 [0.65,1.01] | 0.00 |  | 0.069 | 0.107 | 0.023 |
| Testis | 222 | 0.73 [0.09,2.86] | 1.35 [0.73,2.22] | 1.23 [0.71,1.91] | 1.05 [0.71,1.47] | 1.21 [0.94,1.44] | 0.00 |  | 0.196 | 0.096 | 0.133 |
| Larynx | 208 | 1.50 [0.58,4.27] | **1.84 [1.10,3.36]** | 0.81 [0.47,1.44] | 1.29 [0.93,1.75] | 1.23 [0.99,1.45] | 2.77 |  | 0.024 | 0.027 | 0.155 |
| ^a^ Shown are the estimated risk ratios (RR) with 90% confidence intervals (CI) for 1st to 5th degree (1º-5º) relatives and mates of the 27 cancer sites with ≥200 cases, in bold when the 90% CI does not include 1.00, which corresponds to one-sided p-value <0.05. Also shown are combined p-values (one-sided) to evaluate the significance of the RR for all relatives (1^st^-5^th^ degree) and for close (1^st^ and 2^nd^ degree) and distant relatives (3^rd^-5^th^ degree). | | | | | | | | | | | |
| ^b^ NHL, non-Hodgkin's lymphoma. | | | | | | | | | | | |
| ^c^ na, not applicable (sex-specific cancers). | | | | | | | | | | | |
| ^d^ Number of mates or relatives with cancer/total number of mates or relatives for each cancer site | | | | | | | | | | | |

| **Table 22** RR estimates for relatives and mates of patients diagnosed with myeloid leukaemia for sites with 200 or more cases^a^ | | | | | | | | | | | |
| --- | --- | --- | --- | --- | --- | --- | --- | --- | --- | --- | --- |
| Cancer sites | # of aff. | RR [90% CI] | | | | | |  | combined p-value for relatives | | |
|  |  | 1º relative | 2º relatives | 3º relatives | 4º relatives | 5º relatives | Mates |  | 1º-5º | 1º-2º | 3º-5º |
| Breast | 3812 | **1.42 [1.14,1.67]** | 1.05 [0.88,1.20] | 0.98 [0.87,1.09] | 1.04 [0.94,1.10] | 1.01 [0.94,1.05] | 1.70 |  | 0.051 | 0.478 | 0.559 |
| Prostate | 3380 | **1.34 [1.04,1.59]** | **1.22 [1.03,1.43]** | 1.11 [1.00,1.22] | 0.98 [0.90,1.05] | 1.03 [0.97,1.08] | 1.06 |  | 0.0018 | 0.0259 | 0.1965 |
| Lung | 2904 | 1.03 [0.77,1.31] | 1.20 [0.98,1.42] | 1.07 [0.95,1.22] | 0.97 [0.89,1.06] | 0.98 [0.92,1.04] | 1.54 |  | 0.175 | 0.159 | 0.435 |
| Stomach | 2890 | 1.26 [0.98,1.59] | 1.16 [0.96,1.34] | 1.03 [0.90,1.14] | 0.92 [0.84,1.00] | 1.05 [0.98,1.10] | 1.06 |  | 0.111 | 0.296 | 0.607 |
| Colon | 2224 | 1.03 [0.75,1.40] | 1.10 [0.89,1.34] | 0.95 [0.80,1.08] | 0.91 [0.80,0.99] | 0.94 [0.88,1.01] | 1.46 |  | 0.832 | 0.895 | 0.989 |
| Bladder | 1384 | **1.46 [1.05,2.06]** | 0.90 [0.67,1.22] | **1.29 [1.13,1.55]** | 0.96 [0.84,1.10] | 0.99 [0.90,1.08] | 0.41 |  | 0.030 | 0.200 | 0.072 |
| Kidney | 1227 | 1.35 [0.91,1.91] | 1.04 [0.78,1.35] | 1.05 [0.86,1.27] | **1.24 [1.09,1.39]** | 1.09 [0.98,1.17] | 1.42 |  | 0.0084 | 0.0267 | 0.0096 |
| Thyroid | 957 | 1.08 [0.64,1.68] | 1.13 [0.79,1.49] | 1.12 [0.86,1.35] | 1.02 [0.86,1.17] | 1.09 [0.97,1.19] | 1.97 |  | 0.126 | 0.148 | 0.168 |
| Pancreas | 930 | 1.14 [0.72,1.68] | 0.84 [0.56,1.16] | 1.01 [0.80,1.27] | 1.12 [0.96,1.31] | 1.09 [0.99,1.21] | 1.15 |  | 0.257 | 0.280 | 0.085 |
| Ovary | 906 | 0.99 [0.57,1.56] | 0.93 [0.62,1.34] | 0.83 [0.64,1.06] | 1.02 [0.86,1.19] | 0.90 [0.79,1.00] | 0.55 |  | 0.887 | 0.908 | 0.923 |
| Non-melanoma skin | 781 | 1.28 [0.81,2.06] | 0.96 [0.61,1.33] | 0.85 [0.64,1.08] | 0.97 [0.81,1.15] | 0.91 [0.78,1.01] | 0.71 |  | 0.785 | 0.944 | 0.955 |
| Rectum | 767 | 1.50 [0.97,2.33] | 1.01 [0.70,1.47] | 0.74 [0.56,0.95] | 0.96 [0.81,1.14] | 1.01 [0.91,1.13] | 1.43 |  | 0.410 | 0.817 | 0.890 |
| Endometrium | 753 | 1.49 [0.94,2.34] | 1.00 [0.68,1.42] | 1.11 [0.89,1.41] | 1.01 [0.84,1.20] | 1.09 [0.97,1.21] | 0.59 |  | 0.056 | 0.195 | 0.149 |
| Cervix uteri | 724 | 1.29 [0.71,2.10] | 1.17 [0.75,1.63] | 1.08 [0.84,1.38] | 0.92 [0.75,1.10] | 0.95 [0.83,1.07] | 0.72 |  | 0.322 | 0.515 | 0.667 |
| Brain | 663 | 0.14 [0.01,0.57] | 0.97 [0.60,1.48] | 0.98 [0.71,1.25] | 0.90 [0.72,1.11] | 1.02 [0.88,1.14] | 2.12 |  | 0.966 | 0.735 | 0.730 |
| Melanoma of skin | 618 | **1.73 [1.02,2.78]** | 0.86 [0.53,1.30] | 1.03 [0.73,1.31] | 0.74 [0.56,0.90] | 1.00 [0.83,1.10] | 0.00 |  | 0.625 | 0.973 | 0.967 |
| Esophagus | 535 | **2.12 [1.28,3.31]** | 1.05 [0.64,1.68] | 1.19 [0.85,1.54] | 0.94 [0.77,1.16] | **1.18 [1.02,1.34]** | 1.17 |  | 0.006 | 0.178 | 0.142 |
| Diffuse NHL^b^ | 422 | 1.90 [1.00,3.36] | 1.08 [0.61,1.81] | **1.37 [1.01,1.81]** | 1.20 [0.95,1.48] | 0.91 [0.78,1.07] | 1.39 |  | 0.022 | 0.091 | 0.070 |
| Multiple myeloma | 391 | 1.15 [0.52,2.36] | 0.93 [0.54,1.58] | 0.89 [0.62,1.24] | 0.81 [0.60,1.01] | 1.01 [0.86,1.17] | 1.36 |  | 0.750 | 0.858 | 0.889 |
| Lymphoid leukaemia | 368 | 1.32 [0.54,3.96] | 1.06 [0.58,1.84] | 1.01 [0.65,1.44] | 1.16 [0.86,1.46] | 1.15 [0.96,1.31] | 2.00 |  | 0.186 | 0.234 | 0.193 |
| Myeloid leukaemia | 342 | 0.99 [0.23,3.37] | 0.98 [0.38,2.01] | 1.02 [0.57,1.70] | 0.94 [0.62,1.35] | 0.99 [0.76,1.26] | 0.00 |  | 0.54272 | 0.48934 | 0.56875 |
| Meninges | 291 | 0.84 [0.30,3.08] | 0.74 [0.32,1.57] | 1.01 [0.64,1.43] | 0.99 [0.70,1.32] | 1.01 [0.82,1.22] | 1.97 |  | 0.738 | 0.709 | 0.553 |
| Liver | 257 | 0.60 [0.15,2.61] | 1.23 [0.68,1.92] | 1.27 [0.85,1.80] | 0.85 [0.58,1.13] | 1.02 [0.84,1.24] | 0.00 |  | 0.534 | 0.335 | 0.454 |
| Lip | 244 | 1.49 [0.66,4.25] | 0.93 [0.40,1.76] | 1.27 [0.83,1.84] | 1.02 [0.77,1.40] | 0.88 [0.70,1.09] | 0.00 |  | 0.277 | 0.437 | 0.388 |
| Hodgkin's disease | 239 | 0.48 [0.05,2.36] | 1.18 [0.50,1.98] | 0.77 [0.39,1.25] | 0.72 [0.47,1.02] | 0.94 [0.74,1.15] | 0.00 |  | 0.915 | 0.887 | 0.962 |
| Testis | 222 | 0/1516^d^ | **1.97 [1.17,2.97]** | 0.57 [0.26,0.97] | 0.88 [0.61,1.17] | 0.77 [0.59,0.97] | 0.00 |  | 0.917 | 0.689 | 0.991 |
| Larynx | 208 | 1.42 [0.55,4.12] | 1.18 [0.60,2.38] | 0.94 [0.54,1.59] | 1.15 [0.82,1.57] | 0.88 [0.65,1.09] | 4.49 |  | 0.335 | 0.463 | 0.575 |
| ^a^ Shown are the estimated risk ratios (RR) with 90% confidence intervals (CI) for 1st to 5th degree (1º-5º) relatives and mates of the 27 cancer sites with ≥200 cases, in bold when the 90% CI does not include 1.00, which corresponds to one-sided p-value <0.05. Also shown are combined p-values (one-sided) to evaluate the significance of the RR for all relatives (1^st^-5^th^ degree) and for close (1^st^ and 2^nd^ degree) and distant relatives (3^rd^-5^th^ degree). | | | | | | | | | | | |
| ^b^ NHL, non-Hodgkin's lymphoma. | | | | | | | | | | | |
| ^c^ na, not applicable (sex-specific cancers). | | | | | | | | | | | |
| ^d^ Number of mates or relatives with cancer/total number of mates or relatives for each cancer site | | | | | | | | | | | |

| **Table 23** RR estimates for relatives and mates of patients diagnosed with meningeal cancer for sites with 200 or more cases^a^ | | | | | | | | | | | |
| --- | --- | --- | --- | --- | --- | --- | --- | --- | --- | --- | --- |
| Cancer sites | # of aff. | RR [90% CI] | | | | | |  | combined p-value for relatives | | |
|  |  | 1º relative | 2º relatives | 3º relatives | 4º relatives | 5º relatives | Mates |  | 1º-5º | 1º-2º | 3º-5º |
| Breast | 3812 | **1.34 [1.04,1.63]** | 1.14 [0.95,1.31] | 0.94 [0.82,1.04] | 1.02 [0.93,1.10] | 1.03 [0.97,1.08] | 1.20 |  | 0.044 | 0.286 | 0.588 |
| Prostate | 3380 | 1.18 [0.91,1.46] | 1.15 [0.96,1.36] | 1.08 [0.95,1.20] | **1.11 [1.02,1.21]** | 1.08 [1.00,1.13] | 1.09 |  | 0.0027 | 0.0046 | 0.0077 |
| Lung | 2904 | 1.16 [0.90,1.49] | 1.14 [0.95,1.38] | 1.01 [0.88,1.16] | 1.00 [0.91,1.11] | 0.99 [0.93,1.06] | 1.29 |  | 0.092 | 0.202 | 0.444 |
| Stomach | 2890 | 1.22 [0.93,1.55] | **1.25 [1.04,1.48]** | 0.94 [0.79,1.06] | 1.02 [0.92,1.11] | 0.99 [0.92,1.05] | 1.17 |  | 0.148 | 0.290 | 0.801 |
| Colon | 2224 | 1.27 [0.93,1.66] | 0.95 [0.73,1.19] | 1.01 [0.85,1.15] | 1.04 [0.92,1.14] | **1.09 [1.01,1.16]** | 2.03 |  | 0.111 | 0.281 | 0.153 |
| Bladder | 1384 | 0.71 [0.42,1.13] | 1.11 [0.81,1.48] | 1.06 [0.88,1.28] | 1.06 [0.93,1.22] | **1.15 [1.06,1.25]** | 0.72 |  | 0.189 | 0.024 | 0.019 |
| Kidney | 1227 | 1.14 [0.71,1.67] | 1.05 [0.75,1.36] | 1.06 [0.86,1.28] | 0.99 [0.83,1.14] | 1.05 [0.95,1.14] | 0.99 |  | 0.284 | 0.326 | 0.337 |
| Thyroid | 957 | **1.82 [1.17,2.73]** | 1.29 [0.92,1.70] | 1.16 [0.91,1.39] | 1.04 [0.87,1.20] | 1.01 [0.88,1.10] | 0.91 |  | 0.012 | 0.135 | 0.298 |
| Pancreas | 930 | 1.11 [0.66,1.75] | 1.24 [0.92,1.69] | 1.13 [0.89,1.42] | 1.00 [0.84,1.15] | 1.01 [0.90,1.12] | 2.15 |  | 0.130 | 0.108 | 0.269 |
| Ovary | 906 | 0.82 [0.44,1.45] | 1.29 [0.91,1.74] | 0.85 [0.64,1.10] | 0.98 [0.81,1.15] | **1.14 [1.01,1.27]** | 1.38 |  | 0.421 | 0.251 | 0.495 |
| Non-melanoma skin | 781 | 0.99 [0.54,1.61] | 1.08 [0.71,1.53] | 0.99 [0.73,1.28] | 0.83 [0.65,1.00] | 1.10 [0.97,1.23] | 1.59 |  | 0.575 | 0.587 | 0.685 |
| Rectum | 767 | 1.57 [0.99,2.56] | 1.14 [0.79,1.63] | 0.93 [0.70,1.24] | 1.03 [0.87,1.25] | 0.93 [0.80,1.07] | 1.69 |  | 0.162 | 0.476 | 0.656 |
| Endometrium | 753 | 1.24 [0.75,2.11] | 1.24 [0.85,1.75] | 0.95 [0.74,1.26] | 0.97 [0.79,1.15] | 0.98 [0.84,1.09] | 0.00 |  | 0.291 | 0.434 | 0.719 |
| Cervix uteri | 724 | 1.19 [0.71,1.98] | 1.15 [0.78,1.64] | 0.99 [0.78,1.32] | 1.12 [0.95,1.36] | 1.01 [0.90,1.17] | 1.79 |  | 0.090 | 0.119 | 0.180 |
| Brain | 663 | 1.55 [0.86,2.60] | 0.63 [0.34,1.06] | 0.96 [0.69,1.27] | 1.10 [0.88,1.29] | 0.98 [0.84,1.11] | 2.20 |  | 0.440 | 0.782 | 0.487 |
| Melanoma of skin | 618 | 1.39 [0.68,2.28] | 1.19 [0.76,1.75] | 1.22 [0.88,1.56] | 1.08 [0.87,1.30] | 1.15 [0.98,1.27] | 1.43 |  | 0.047 | 0.078 | 0.095 |
| Esophagus | 535 | 0.84 [0.35,1.59] | 1.05 [0.62,1.62] | 1.00 [0.66,1.30] | **1.32 [1.09,1.60]** | 1.04 [0.88,1.19] | 0.00 |  | 0.250 | 0.143 | 0.099 |
| Diffuse NHL^b^ | 422 | 1.48 [0.62,2.73] | 0.37 [0.12,0.89] | 1.07 [0.73,1.48] | 1.07 [0.85,1.38] | 1.06 [0.89,1.24] | 1.38 |  | 0.505 | 0.741 | 0.227 |
| Multiple myeloma | 391 | 0.43 [0.07,1.17] | 1.27 [0.74,2.17] | **1.55 [1.11,2.06]** | 0.77 [0.54,0.97] | 1.06 [0.90,1.25] | 1.52 |  | 0.439 | 0.140 | 0.223 |
| Lymphoid leukaemia | 368 | **2.32 [1.13,5.59]** | 1.09 [0.61,1.84] | 0.59 [0.33,0.96] | 1.18 [0.86,1.46] | 0.92 [0.74,1.10] | 0.00 |  | 0.239 | 0.799 | 0.887 |
| Myeloid leukaemia | 342 | 0.84 [0.30,3.08] | 0.74 [0.32,1.57] | 1.01 [0.64,1.43] | 0.99 [0.70,1.32] | 1.01 [0.82,1.22] | 1.97 |  | 0.738 | 0.709 | 0.553 |
| Meninges | 291 | **3.15 [1.50,6.84]** | 1.36 [0.54,4.03] | 1.60 [0.94,2.70] | 1.47 [1.00,2.06] | 1.20 [0.89,1.53] | 0.00 |  | 0.00141 | 0.01853 | 0.01051 |
| Liver | 257 | 0.66 [0.15,2.73] | 1.33 [0.63,2.67] | 1.48 [0.92,2.16] | 0.92 [0.62,1.28] | 1.15 [0.91,1.37] | 4.04 |  | 0.273 | 0.124 | 0.168 |
| Lip | 244 | 0.99 [0.30,3.38] | **1.79 [1.07,2.86]** | 1.09 [0.69,1.67] | 1.02 [0.73,1.34] | 1.00 [0.78,1.21] | 0.00 |  | 0.157 | 0.123 | 0.489 |
| Hodgkin's disease | 239 | 1.07 [0.25,3.51] | 0.22 [0.02,0.89] | 1.28 [0.80,1.97] | 0.97 [0.64,1.35] | 0.98 [0.74,1.24] | 0.00 |  | 0.708 | 0.829 | 0.395 |
| Testis | 222 | 2.18 [0.85,5.36] | 0.90 [0.30,1.95] | 1.18 [0.65,1.91] | 1.15 [0.79,1.61] | 0.78 [0.56,1.01] | 0.00 |  | 0.239 | 0.575 | 0.551 |
| Larynx | 208 | 1.57 [0.50,4.39] | 1.60 [0.74,2.77] | 0.93 [0.50,1.62] | 0.90 [0.58,1.29] | 1.19 [0.92,1.47] | 0.00 |  | 0.158 | 0.251 | 0.443 |
| ^a^ Shown are the estimated risk ratios (RR) with 90% confidence intervals (CI) for 1st to 5th degree (1º-5º) relatives and mates of the 27 cancer sites with ≥200 cases, in bold when the 90% CI does not include 1.00, which corresponds to one-sided p-value <0.05. Also shown are combined p-values (one-sided) to evaluate the significance of the RR for all relatives (1^st^-5^th^ degree) and for close (1^st^ and 2^nd^ degree) and distant relatives (3^rd^-5^th^ degree). | | | | | | | | | | | |
| ^b^ NHL, non-Hodgkin's lymphoma. | | | | | | | | | | | |
| ^c^ na, not applicable (sex-specific cancers). | | | | | | | | | | | |
| ^d^ Number of mates or relatives with cancer/total number of mates or relatives for each cancer site | | | | | | | | | | | |

| **Table 24** RR estimates for relatives and mates of patients diagnosed with liver cancer for sites with 200 or more cases^a^ | | | | | | | | | | | |
| --- | --- | --- | --- | --- | --- | --- | --- | --- | --- | --- | --- |
| Cancer sites | # of aff. | RR [90% CI] | | | | | |  | combined p-value for relatives | | |
|  |  | 1º relative | 2º relatives | 3º relatives | 4º relatives | 5º relatives | Mates |  | 1º-5º | 1º-2º | 3º-5º |
| Breast | 3812 | 0.98 [0.76,1.26] | 1.15 [0.96,1.34] | 1.05 [0.90,1.17] | 1.00 [0.91,1.08] | 1.07 [1.00,1.11] | 1.02 |  | 0.139 | 0.077 | 0.218 |
| Prostate | 3380 | 1.02 [0.77,1.29] | 0.88 [0.70,1.09] | 1.05 [0.92,1.17] | 1.00 [0.89,1.09] | 1.06 [0.99,1.11] | 1.87 |  | 0.529 | 0.545 | 0.239 |
| Lung | 2904 | **1.62 [1.29,2.02]** | 0.91 [0.73,1.17] | 1.02 [0.88,1.17] | 1.06 [0.96,1.16] | 1.00 [0.94,1.06] | 1.57 |  | 0.017 | 0.405 | 0.215 |
| Stomach | 2890 | 1.24 [0.98,1.58] | 0.85 [0.67,1.05] | 1.03 [0.89,1.17] | 1.02 [0.91,1.12] | 0.99 [0.91,1.04] | 2.11 |  | 0.449 | 0.788 | 0.508 |
| Colon | 2224 | 1.22 [0.89,1.64] | 1.08 [0.82,1.35] | 0.96 [0.80,1.12] | 0.92 [0.81,1.03] | 1.04 [0.94,1.10] | 1.00 |  | 0.416 | 0.660 | 0.806 |
| Bladder | 1384 | 1.23 [0.85,1.81] | 1.17 [0.87,1.55] | 1.14 [0.93,1.38] | 1.03 [0.90,1.21] | 0.99 [0.90,1.10] | 0.94 |  | 0.050 | 0.107 | 0.188 |
| Kidney | 1227 | 0.80 [0.48,1.33] | 0.90 [0.61,1.28] | 1.09 [0.84,1.33] | 0.93 [0.78,1.09] | 1.00 [0.89,1.09] | 1.59 |  | 0.749 | 0.679 | 0.588 |
| Thyroid | 957 | 1.49 [0.88,2.48] | 1.03 [0.68,1.44] | 1.20 [0.93,1.50] | 0.85 [0.68,1.02] | 1.11 [0.98,1.21] | 1.37 |  | 0.108 | 0.322 | 0.335 |
| Pancreas | 930 | 1.33 [0.86,1.95] | 1.23 [0.88,1.70] | 1.23 [0.98,1.51] | 0.90 [0.75,1.09] | 1.00 [0.88,1.12] | 2.29 |  | 0.083 | 0.155 | 0.333 |
| Ovary | 906 | 0.63 [0.31,1.16] | 1.03 [0.68,1.45] | 0.92 [0.68,1.16] | 1.00 [0.81,1.21] | 1.13 [0.99,1.26] | 1.57 |  | 0.639 | 0.404 | 0.411 |
| Non-melanoma skin | 781 | 0.88 [0.44,1.50] | 0.76 [0.43,1.16] | 1.11 [0.85,1.44] | 0.95 [0.75,1.17] | 1.07 [0.94,1.20] | 0.75 |  | 0.681 | 0.623 | 0.322 |
| Rectum | 767 | 1.44 [0.84,2.36] | **1.67 [1.19,2.39]** | 1.11 [0.87,1.48] | 1.05 [0.85,1.26] | 0.94 [0.83,1.08] | 0.75 |  | 0.009 | 0.026 | 0.296 |
| Endometrium | 753 | 0.79 [0.39,1.53] | 0.53 [0.29,0.92] | 1.02 [0.76,1.36] | 0.90 [0.73,1.09] | 0.81 [0.70,0.93] | 0.54 |  | 0.989 | 0.992 | 0.936 |
| Cervix uteri | 724 | **1.81 [1.12,2.97]** | 1.08 [0.69,1.74] | 1.13 [0.86,1.49] | 0.80 [0.63,0.99] | 1.06 [0.93,1.22] | 0.00 |  | 0.077 | 0.400 | 0.514 |
| Brain | 663 | 1.20 [0.55,2.04] | 0.95 [0.53,1.62] | 1.16 [0.88,1.50] | 0.95 [0.73,1.15] | **1.21 [1.05,1.37]** | 2.46 |  | 0.222 | 0.211 | 0.113 |
| Melanoma of skin | 618 | 1.58 [0.78,2.94] | 1.11 [0.65,1.65] | 1.19 [0.82,1.53] | 1.10 [0.86,1.34] | 0.94 [0.77,1.07] | 1.40 |  | 0.171 | 0.386 | 0.413 |
| Esophagus | 535 | **1.93 [1.24,3.08]** | 0.78 [0.42,1.41] | 1.17 [0.81,1.53] | 1.02 [0.81,1.23] | 0.88 [0.74,1.02] | 1.19 |  | 0.221 | 0.724 | 0.557 |
| Diffuse NHL^b^ | 422 | 1.13 [0.55,2.22] | 1.12 [0.59,1.94] | 0.89 [0.61,1.34] | 1.07 [0.83,1.38] | 1.00 [0.83,1.21] | 3.34 |  | 0.364 | 0.418 | 0.482 |
| Multiple myeloma | 391 | 1.29 [0.69,2.47] | 1.63 [0.96,2.53] | 1.01 [0.63,1.46] | 0.90 [0.67,1.20] | **1.22 [1.03,1.45]** | 0.00 |  | 0.045 | 0.056 | 0.278 |
| Lymphoid leukaemia | 368 | 0.92 [0.30,3.23] | 1.53 [0.88,2.54] | 0.99 [0.61,1.48] | 0.98 [0.71,1.31] | 1.09 [0.89,1.27] | 0.00 |  | 0.252 | 0.211 | 0.500 |
| Myeloid leukaemia | 342 | 0.60 [0.15,2.61] | 1.23 [0.68,1.92] | 1.27 [0.85,1.80] | 0.85 [0.58,1.13] | 1.02 [0.84,1.24] | 0.00 |  | 0.534 | 0.335 | 0.454 |
| Meninges | 291 | 0.66 [0.15,2.73] | 1.33 [0.63,2.67] | 1.48 [0.92,2.16] | 0.92 [0.62,1.28] | 1.15 [0.91,1.37] | 4.04 |  | 0.273 | 0.124 | 0.168 |
| Liver | 257 | 2.00 [0.71,5.08] | 1.28 [0.41,3.89] | 0.64 [0.24,1.35] | 1.14 [0.70,1.74] | 1.02 [0.73,1.36] | 0.00 |  | 0.23887 | 0.13249 | 0.60908 |
| Lip | 244 | 0.96 [0.23,3.30] | 1.08 [0.45,2.18] | 1.15 [0.70,1.75] | 1.02 [0.69,1.43] | 0.74 [0.56,0.93] | 2.78 |  | 0.650 | 0.641 | 0.725 |
| Hodgkin's disease | 239 | 1.28 [0.36,3.89] | **2.25 [1.23,3.73]** | 0.63 [0.32,1.21] | 1.28 [0.94,1.76] | 0.77 [0.58,1.00] | 0.00 |  | 0.150 | 0.146 | 0.751 |
| Testis | 222 | 0/1516^d^ | 1.41 [0.71,2.59] | 0.69 [0.31,1.37] | 1.07 [0.70,1.58] | 1.20 [0.94,1.51] | 0.00 |  | 0.510 | 0.205 | 0.405 |
| Larynx | 208 | **3.46 [1.76,7.29]** | 1.03 [0.40,2.02] | **1.79 [1.16,2.65]** | 1.34 [0.94,1.91] | 0.84 [0.61,1.09] | 0.00 |  | 0.0029 | 0.11455 | 0.05175 |
| ^a^ Shown are the estimated risk ratios (RR) with 90% confidence intervals (CI) for 1st to 5th degree (1º-5º) relatives and mates of the 27 cancer sites with ≥200 cases, in bold when the 90% CI does not include 1.00, which corresponds to one-sided p-value <0.05. Also shown are combined p-values (one-sided) to evaluate the significance of the RR for all relatives (1^st^-5^th^ degree) and for close (1^st^ and 2^nd^ degree) and distant relatives (3^rd^-5^th^ degree). | | | | | | | | | | | |
| ^b^ NHL, non-Hodgkin's lymphoma. | | | | | | | | | | | |
| ^c^ na, not applicable (sex-specific cancers). | | | | | | | | | | | |
| ^d^ Number of mates or relatives with cancer/total number of mates or relatives for each cancer site | | | | | | | | | | | |

| **Table 25** RR estimates for relatives and mates of patients diagnosed with lip cancer for sites with 200 or more cases^a^ | | | | | | | | | | | |
| --- | --- | --- | --- | --- | --- | --- | --- | --- | --- | --- | --- |
| Cancer sites | # of aff. | RR [90% CI] | | | | | |  | combined p-value for relatives | | |
|  |  | 1º relative | 2º relatives | 3º relatives | 4º relatives | 5º relatives | Mates |  | 1º-5º | 1º-2º | 3º-5º |
| Breast | 3812 | 0.76 [0.54,0.99] | 0.86 [0.69,1.04] | 0.99 [0.85,1.12] | 0.89 [0.80,0.96] | 1.05 [0.98,1.11] | 1.52 |  | 0.986 | 0.957 | 0.831 |
| Prostate | 3380 | 1.11 [0.85,1.43] | 0.94 [0.74,1.18] | 1.09 [0.96,1.23] | **1.17 [1.05,1.26]** | 1.00 [0.93,1.05] | 0.00 |  | 0.091 | 0.096 | 0.021 |
| Lung | 2904 | 1.08 [0.78,1.40] | 1.03 [0.81,1.29] | **1.19 [1.04,1.36]** | 1.04 [0.94,1.15] | 0.89 [0.83,0.96] | 1.21 |  | 0.272 | 0.290 | 0.291 |
| Stomach | 2890 | 1.30 [0.99,1.61] | 1.09 [0.86,1.32] | 1.05 [0.90,1.18] | 0.98 [0.88,1.07] | 1.03 [0.96,1.09] | 0.80 |  | 0.128 | 0.365 | 0.450 |
| Colon | 2224 | 0.85 [0.57,1.22] | 0.94 [0.69,1.22] | 0.94 [0.80,1.10] | 1.01 [0.89,1.12] | 0.95 [0.87,1.01] | 1.83 |  | 0.902 | 0.866 | 0.855 |
| Bladder | 1384 | 0.84 [0.50,1.30] | 1.11 [0.83,1.51] | 1.06 [0.87,1.28] | 1.04 [0.88,1.20] | 1.02 [0.92,1.13] | 3.21 |  | 0.324 | 0.170 | 0.253 |
| Kidney | 1227 | 0.79 [0.47,1.34] | 0.89 [0.60,1.25] | 0.93 [0.71,1.11] | 1.09 [0.94,1.26] | 1.00 [0.88,1.09] | 0.62 |  | 0.807 | 0.706 | 0.547 |
| Thyroid | 957 | 1.06 [0.59,1.69] | 1.04 [0.72,1.47] | 0.94 [0.71,1.19] | 1.17 [0.97,1.35] | 1.13 [0.99,1.26] | 0.67 |  | 0.214 | 0.160 | 0.118 |
| Pancreas | 930 | 1.38 [0.90,2.11] | 0.72 [0.42,1.09] | 1.26 [1.00,1.53] | 0.88 [0.71,1.09] | 1.02 [0.91,1.14] | 0.63 |  | 0.360 | 0.640 | 0.268 |
| Ovary | 906 | 0.43 [0.14,0.84] | 0.93 [0.60,1.39] | 1.05 [0.81,1.34] | 1.07 [0.88,1.26] | **1.15 [1.02,1.27]** | 0.94 |  | 0.657 | 0.208 | 0.104 |
| Non-melanoma skin | 781 | 1.40 [0.82,2.09] | 1.31 [0.84,1.95] | 0.99 [0.72,1.31] | 0.99 [0.80,1.21] | 1.03 [0.89,1.14] | 1.73 |  | 0.133 | 0.254 | 0.561 |
| Rectum | 767 | 0.86 [0.42,1.60] | 1.26 [0.78,1.83] | 1.16 [0.92,1.51] | 0.76 [0.59,0.96] | **1.17 [1.06,1.33]** | 0.83 |  | 0.299 | 0.172 | 0.252 |
| Endometrium | 753 | 1.03 [0.61,1.80] | 0.52 [0.27,0.90] | 0.84 [0.57,1.18] | 0.99 [0.78,1.20] | 1.07 [0.92,1.20] | 1.46 |  | 0.862 | 0.914 | 0.656 |
| Cervix uteri | 724 | 0.58 [0.24,1.27] | 1.08 [0.68,1.61] | 1.18 [0.86,1.55] | 1.06 [0.85,1.29] | 0.89 [0.75,1.03] | 0.66 |  | 0.602 | 0.354 | 0.382 |
| Brain | 663 | 0.87 [0.31,1.98] | 0.78 [0.42,1.37] | 1.20 [0.85,1.54] | 1.13 [0.92,1.36] | 0.90 [0.75,1.03] | 3.12 |  | 0.648 | 0.563 | 0.351 |
| Melanoma of skin | 618 | 1.25 [0.58,2.43] | 1.02 [0.63,1.61] | 1.23 [0.92,1.63] | 1.15 [0.89,1.37] | 1.09 [0.91,1.22] | 1.56 |  | 0.126 | 0.138 | 0.087 |
| Esophagus | 535 | 1.69 [1.00,2.81] | 1.10 [0.66,1.66] | 0.57 [0.35,0.86] | 1.11 [0.87,1.38] | 1.06 [0.91,1.21] | 1.37 |  | 0.287 | 0.707 | 0.804 |
| Diffuse NHL^b^ | 422 | 0.91 [0.41,1.84] | 1.31 [0.76,2.39] | 1.33 [0.96,1.83] | 0.76 [0.56,1.00] | 0.97 [0.80,1.16] | 2.04 |  | 0.337 | 0.275 | 0.509 |
| Multiple myeloma | 391 | 1.49 [0.74,2.75] | 1.08 [0.59,2.03] | 0.70 [0.45,1.05] | 0.74 [0.51,0.99] | 0.96 [0.77,1.15] | 3.32 |  | 0.748 | 0.920 | 0.979 |
| Lymphoid leukaemia | 368 | 1.49 [0.69,4.25] | 1.56 [0.91,2.57] | **1.51 [1.05,2.08]** | 1.14 [0.85,1.45] | 0.96 [0.77,1.16] | 0.00 |  | 0.024 | 0.037 | 0.089 |
| Myeloid leukaemia | 342 | 1.49 [0.66,4.25] | 0.93 [0.40,1.76] | 1.27 [0.83,1.84] | 1.02 [0.77,1.40] | 0.88 [0.70,1.09] | 0.00 |  | 0.277 | 0.437 | 0.388 |
| Meninges | 291 | 0.99 [0.30,3.38] | **1.79 [1.07,2.86]** | 1.09 [0.69,1.67] | 1.02 [0.73,1.34] | 1.00 [0.78,1.21] | 0.00 |  | 0.157 | 0.123 | 0.489 |
| Liver | 257 | 0.96 [0.23,3.30] | 1.08 [0.45,2.18] | 1.15 [0.70,1.75] | 1.02 [0.69,1.43] | 0.74 [0.56,0.93] | 2.78 |  | 0.650 | 0.641 | 0.725 |
| Lip | 244 | **5.04 [2.75,9.52]** | 0.49 [0.03,2.37] | 0.91 [0.40,1.73] | 0.63 [0.30,1.12] | **1.41 [1.06,1.80]** | 0.00 |  | 0.02346 | 0.0091 | 0.48947 |
| Hodgkin's disease | 239 | 0.72 [0.09,2.85] | **2.95 [1.90,4.59]** | 0.97 [0.50,1.52] | 0.85 [0.52,1.22] | 1.14 [0.89,1.42] | 0.00 |  | 0.080 | 0.018 | 0.626 |
| Testis | 222 | 1.37 [0.08,4.04] | 0.50 [0.12,1.26] | 1.05 [0.59,1.69] | 1.16 [0.74,1.62] | 0.74 [0.52,0.97] | 0.00 |  | 0.686 | 0.863 | 0.690 |
| Larynx | 208 | 0.90 [0.21,3.19] | 0.78 [0.26,1.68] | 1.03 [0.55,1.79] | 0.87 [0.52,1.28] | 0.78 [0.54,1.02] | 0.00 |  | 0.870 | 0.890 | 0.864 |
| ^a^ Shown are the estimated risk ratios (RR) with 90% confidence intervals (CI) for 1st to 5th degree (1º-5º) relatives and mates of the 27 cancer sites with ≥200 cases, in bold when the 90% CI does not include 1.00, which corresponds to one-sided p-value <0.05. Also shown are combined p-values (one-sided) to evaluate the significance of the RR for all relatives (1^st^-5^th^ degree) and for close (1^st^ and 2^nd^ degree) and distant relatives (3^rd^-5^th^ degree). | | | | | | | | | | | |
| ^b^ NHL, non-Hodgkin's lymphoma. | | | | | | | | | | | |
| ^c^ na, not applicable (sex-specific cancers). | | | | | | | | | | | |
| ^d^ Number of mates or relatives with cancer/total number of mates or relatives for each cancer site | | | | | | | | | | | |

| **Table 26** RR estimates for relatives and mates of patients diagnosed with Hodgkin's disease for sites with 200 or more cases^a^ | | | | | | | | | | | |
| --- | --- | --- | --- | --- | --- | --- | --- | --- | --- | --- | --- |
| Cancer sites | # of aff. | RR [90% CI] | | | | | |  | combined p-value for relatives | | |
|  |  | 1º relative | 2º relatives | 3º relatives | 4º relatives | 5º relatives | Mates |  | 1º-5º | 1º-2º | 3º-5º |
| Breast | 3812 | 1.04 [0.76,1.42] | 0.95 [0.75,1.13] | **1.16 [1.01,1.31]** | 1.02 [0.93,1.10] | 1.02 [0.95,1.08] | 1.23 |  | 0.235 | 0.229 | 0.076 |
| Prostate | 3380 | 0.96 [0.65,1.30] | 1.05 [0.88,1.27] | 1.09 [0.94,1.23] | 0.99 [0.90,1.09] | 1.02 [0.94,1.07] | 1.25 |  | 0.356 | 0.238 | 0.290 |
| Lung | 2904 | 0.80 [0.52,1.14] | 1.00 [0.79,1.26] | 1.01 [0.85,1.17] | 1.06 [0.94,1.18] | 0.98 [0.91,1.05] | 1.31 |  | 0.666 | 0.449 | 0.428 |
| Stomach | 2890 | 1.16 [0.76,1.61] | **1.35 [1.10,1.64]** | 0.90 [0.77,1.06] | **1.14 [1.02,1.24]** | 1.07 [0.98,1.13] | 1.18 |  | 0.021 | 0.016 | 0.146 |
| Colon | 2224 | 1.07 [0.71,1.60] | 1.27 [0.98,1.60] | 1.18 [0.96,1.36] | 1.06 [0.93,1.17] | 1.00 [0.92,1.07] | 1.21 |  | 0.061 | 0.031 | 0.128 |
| Bladder | 1384 | 1.21 [0.72,1.95] | 0.96 [0.68,1.32] | 0.80 [0.62,1.03] | 0.91 [0.77,1.08] | 1.09 [0.98,1.21] | 0.00 |  | 0.555 | 0.764 | 0.779 |
| Kidney | 1227 | 0.39 [0.13,0.87] | **1.42 [1.04,1.91]** | 1.12 [0.89,1.38] | 1.03 [0.86,1.21] | 1.02 [0.89,1.12] | 0.00 |  | 0.442 | 0.047 | 0.263 |
| Thyroid | 957 | 1.22 [0.58,2.52] | 1.04 [0.69,1.47] | **1.48 [1.16,1.86]** | 1.17 [0.97,1.36] | 0.94 [0.81,1.06] | 1.26 |  | 0.061 | 0.061 | 0.021 |
| Pancreas | 930 | 0.87 [0.35,1.66] | 1.34 [0.91,1.91] | 1.00 [0.78,1.31] | 0.94 [0.76,1.14] | 1.04 [0.90,1.17] | 0.00 |  | 0.339 | 0.213 | 0.511 |
| Ovary | 906 | 0.88 [0.37,1.63] | 1.03 [0.67,1.54] | 1.00 [0.72,1.34] | 1.04 [0.85,1.28] | 0.85 [0.74,0.98] | 1.07 |  | 0.699 | 0.665 | 0.749 |
| Non-melanoma skin | 781 | 1.64 [0.91,3.00] | 1.13 [0.72,1.68] | 0.94 [0.67,1.25] | 0.80 [0.64,0.99] | 1.01 [0.87,1.15] | 0.00 |  | 0.356 | 0.740 | 0.897 |
| Rectum | 767 | 1.03 [0.45,2.13] | 0.91 [0.57,1.39] | 1.09 [0.79,1.46] | 1.15 [0.94,1.39] | 1.07 [0.92,1.21] | 3.52 |  | 0.296 | 0.249 | 0.129 |
| Endometrium | 753 | 1.38 [0.70,2.69] | 0.88 [0.52,1.37] | 1.28 [0.95,1.66] | 1.10 [0.89,1.35] | 0.98 [0.83,1.12] | 2.46 |  | 0.153 | 0.234 | 0.114 |
| Cervix uteri | 724 | 1.57 [0.83,2.89] | 0.53 [0.28,0.94] | 1.25 [0.88,1.62] | **1.25 [1.03,1.56]** | 1.12 [0.97,1.28] | 1.37 |  | 0.078 | 0.198 | 0.010 |
| Brain | 663 | 1.03 [0.45,2.07] | 1.25 [0.81,2.04] | 0.64 [0.41,0.95] | 0.94 [0.71,1.16] | 1.09 [0.91,1.25] | 0.00 |  | 0.541 | 0.601 | 0.878 |
| Melanoma of skin | 618 | 0.29 [0.01,1.89] | 0.68 [0.34,1.26] | 1.09 [0.74,1.48] | 1.23 [0.97,1.45] | 1.04 [0.87,1.18] | 0.00 |  | 0.824 | 0.492 | 0.193 |
| Esophagus | 535 | 1.64 [0.69,4.50] | 0.60 [0.24,1.11] | 0.80 [0.49,1.13] | 1.09 [0.85,1.36] | 0.77 [0.62,0.90] | 0.00 |  | 0.815 | 0.979 | 0.951 |
| Diffuse NHL^b^ | 422 | **2.56 [1.40,5.95]** | 0.78 [0.38,1.42] | 1.15 [0.78,1.65] | 0.89 [0.66,1.19] | 0.94 [0.76,1.14] | 0.00 |  | 0.084 | 0.658 | 0.519 |
| Multiple myeloma | 391 | 0.78 [0.20,2.98] | **2.58 [1.78,3.66]** | 0.97 [0.63,1.48] | 1.12 [0.84,1.43] | 1.05 [0.87,1.29] | 0.00 |  | 0.0228 | 0.0027 | 0.2974 |
| Lymphoid leukaemia | 368 | 1.55 [0.54,4.35] | 0.77 [0.35,1.48] | **1.98 [1.47,2.72]** | 1.13 [0.80,1.45] | 0.83 [0.65,1.01] | 0.00 |  | 0.069 | 0.107 | 0.023 |
| Myeloid leukaemia | 342 | 0.48 [0.05,2.36] | 1.18 [0.50,1.98] | 0.77 [0.39,1.25] | 0.72 [0.47,1.02] | 0.94 [0.74,1.15] | 0.00 |  | 0.915 | 0.887 | 0.962 |
| Meninges | 291 | 1.07 [0.25,3.51] | 0.22 [0.02,0.89] | 1.28 [0.80,1.97] | 0.97 [0.64,1.35] | 0.98 [0.74,1.24] | 0.00 |  | 0.708 | 0.829 | 0.395 |
| Liver | 257 | 1.28 [0.36,3.89] | **2.25 [1.23,3.73]** | 0.63 [0.32,1.21] | 1.28 [0.94,1.76] | 0.77 [0.58,1.00] | 0.00 |  | 0.150 | 0.146 | 0.751 |
| Lip | 244 | 0.72 [0.09,2.85] | **2.95 [1.90,4.59]** | 0.97 [0.50,1.52] | 0.85 [0.52,1.22] | 1.14 [0.89,1.42] | 0.00 |  | 0.080 | 0.018 | 0.626 |
| Hodgkin's disease | 239 | **3.27 [1.19,7.01]** | 1.85 [0.61,4.84] | 0.79 [0.26,1.74] | 0.94 [0.50,1.57] | 0.79 [0.50,1.16] | 0.00 |  | 0.11431 | 0.03064 | 0.81722 |
| Testis | 222 | 1.94 [0.67,4.99] | 0.69 [0.12,2.80] | 0.99 [0.50,1.67] | 0.62 [0.35,0.94] | 0.91 [0.65,1.18] | 0.00 |  | 0.625 | 0.933 | 0.916 |
| Larynx | 208 | 1.34 [0.28,3.98] | 1.89 [0.95,3.18] | 1.31 [0.74,2.14] | 1.14 [0.69,1.65] | 0.86 [0.61,1.13] | 0.00 |  | 0.084 | 0.095 | 0.342 |
| ^a^ Shown are the estimated risk ratios (RR) with 90% confidence intervals (CI) for 1st to 5th degree (1º-5º) relatives and mates of the 27 cancer sites with ≥200 cases, in bold when the 90% CI does not include 1.00, which corresponds to one-sided p-value <0.05. Also shown are combined p-values (one-sided) to evaluate the significance of the RR for all relatives (1^st^-5^th^ degree) and for close (1^st^ and 2^nd^ degree) and distant relatives (3^rd^-5^th^ degree). | | | | | | | | | | | |
| ^b^ NHL, non-Hodgkin's lymphoma. | | | | | | | | | | | |
| ^c^ na, not applicable (sex-specific cancers). | | | | | | | | | | | |
| ^d^ Number of mates or relatives with cancer/total number of mates or relatives for each cancer site | | | | | | | | | | | |

| **Table 27** RR estimates for relatives and mates of patients diagnosed with testis cancer for sites with 200 or more cases^a^ | | | | | | | | | | | |
| --- | --- | --- | --- | --- | --- | --- | --- | --- | --- | --- | --- |
| Cancer sites | # of aff. | RR [90% CI] | | | | | |  | combined p-value for relatives | | |
|  |  | 1º relative | 2º relatives | 3º relatives | 4º relatives | 5º relatives | Mates |  | 1º-5º | 1º-2º | 3º-5º |
| Breast | 3812 | 0.85 [0.57,1.24] | 0.85 [0.68,1.06] | **1.23 [1.04,1.37]** | 0.95 [0.85,1.04] | 1.06 [0.98,1.11] | 1.95 |  | 0.583 | 0.363 | 0.088 |
| Prostate | 3380 | **1.59 [1.16,2.28]** | **1.23 [1.01,1.46]** | 0.86 [0.71,1.01] | **1.17 [1.06,1.28]** | 1.04 [0.96,1.10] | na^c^ |  | 0.0031 | 0.0621 | 0.2653 |
| Lung | 2904 | 1.18 [0.84,1.74] | 0.80 [0.61,1.03] | 0.94 [0.78,1.10] | **1.11 [1.01,1.24]** | **1.11 [1.03,1.20]** | 0.00 |  | 0.222 | 0.335 | 0.051 |
| Stomach | 2890 | 0.82 [0.44,1.35] | **1.35 [1.10,1.63]** | 0.99 [0.83,1.14] | 1.08 [0.95,1.19] | 1.05 [0.96,1.11] | 0.00 |  | 0.171 | 0.026 | 0.242 |
| Colon | 2224 | 1.40 [0.90,2.16] | 0.96 [0.70,1.22] | **1.33 [1.10,1.56]** | 0.95 [0.82,1.09] | 1.04 [0.94,1.12] | 0.00 |  | 0.064 | 0.165 | 0.069 |
| Bladder | 1384 | 1.22 [0.66,2.20] | 1.28 [0.94,1.71] | 0.99 [0.77,1.28] | 0.98 [0.83,1.15] | 1.02 [0.91,1.14] | 0.00 |  | 0.137 | 0.187 | 0.477 |
| Kidney | 1227 | 1.62 [0.96,2.58] | 0.81 [0.52,1.24] | 1.27 [1.00,1.59] | 0.97 [0.80,1.15] | 1.05 [0.92,1.16] | 0.00 |  | 0.101 | 0.330 | 0.138 |
| Thyroid | 957 | 1.68 [0.86,3.01] | 1.46 [0.94,1.99] | **1.40 [1.06,1.73]** | 1.14 [0.94,1.34] | 0.95 [0.81,1.06] | 1.34 |  | 0.009 | 0.016 | 0.070 |
| Pancreas | 930 | 0.90 [0.31,1.83] | 1.20 [0.82,1.76] | 1.03 [0.79,1.37] | 1.05 [0.87,1.27] | **1.15 [1.01,1.32]** | 0.00 |  | 0.189 | 0.070 | 0.091 |
| Ovary | 906 | 0.71 [0.28,1.68] | 1.05 [0.64,1.56] | 0.85 [0.59,1.12] | 0.77 [0.60,0.94] | 1.00 [0.87,1.17] | 1.11 |  | 0.905 | 0.900 | 0.972 |
| Non-melanoma skin | 781 | 0.65 [0.19,2.71] | 0.46 [0.21,0.88] | 1.25 [0.92,1.64] | 0.95 [0.75,1.17] | 0.96 [0.80,1.10] | 0.00 |  | 0.907 | 0.872 | 0.433 |
| Rectum | 767 | 1.03 [0.37,3.45] | 1.01 [0.64,1.58] | 0.95 [0.65,1.26] | 1.00 [0.78,1.25] | 1.04 [0.90,1.21] | 0.00 |  | 0.507 | 0.512 | 0.545 |
| Endometrium | 753 | 0.56 [0.12,2.53] | 0.77 [0.47,1.29] | 1.04 [0.77,1.46] | 1.13 [0.90,1.37] | 1.06 [0.90,1.22] | 0.00 |  | 0.613 | 0.381 | 0.179 |
| Cervix uteri | 724 | 0.57 [0.14,1.36] | 1.29 [0.83,2.00] | 1.01 [0.69,1.41] | 0.91 [0.70,1.15] | 1.10 [0.93,1.26] | 1.18 |  | 0.528 | 0.289 | 0.500 |
| Brain | 663 | 1.31 [0.59,2.33] | 0.98 [0.55,1.66] | 1.18 [0.82,1.64] | 0.85 [0.63,1.06] | 1.10 [0.91,1.25] | 3.16 |  | 0.379 | 0.506 | 0.472 |
| Melanoma of skin | 618 | 0.34 [0.01,2.03] | 0.76 [0.35,1.47] | 0.84 [0.55,1.26] | 1.03 [0.77,1.28] | 1.02 [0.82,1.17] | 1.86 |  | 0.937 | 0.818 | 0.729 |
| Esophagus | 535 | **2.37 [1.04,5.66]** | 0.93 [0.45,1.68] | 1.08 [0.74,1.59] | 1.19 [0.90,1.46] | 1.10 [0.92,1.30] | 0.00 |  | 0.050 | 0.220 | 0.125 |
| Diffuse NHL^b^ | 422 | 2.06 [0.97,5.17] | 1.20 [0.72,2.17] | 1.04 [0.66,1.65] | 1.02 [0.74,1.34] | 1.01 [0.82,1.24] | 0.00 |  | 0.059 | 0.261 | 0.385 |
| Multiple myeloma | 391 | 1.24 [0.29,3.81] | 1.51 [0.87,2.58] | 0.95 [0.61,1.52] | 1.31 [0.97,1.66] | 1.06 [0.86,1.29] | 0.00 |  | 0.154 | 0.079 | 0.160 |
| Lymphoid leukaemia | 368 | 0.73 [0.09,2.86] | 1.35 [0.73,2.22] | 1.23 [0.71,1.91] | 1.05 [0.71,1.47] | 1.21 [0.94,1.44] | 0.00 |  | 0.196 | 0.096 | 0.133 |
| Myeloid leukaemia | 342 | 0/1516^d^ | **1.97 [1.17,2.97]** | 0.57 [0.26,0.97] | 0.88 [0.61,1.17] | 0.77 [0.59,0.97] | 0.00 |  | 0.917 | 0.689 | 0.991 |
| Meninges | 291 | 2.18 [0.85,5.36] | 0.90 [0.30,1.95] | 1.18 [0.65,1.91] | 1.15 [0.79,1.61] | 0.78 [0.56,1.01] | 0.00 |  | 0.239 | 0.575 | 0.551 |
| Liver | 257 | 0/1516^d^ | 1.41 [0.71,2.59] | 0.69 [0.31,1.37] | 1.07 [0.70,1.58] | 1.20 [0.94,1.51] | 0.00 |  | 0.510 | 0.205 | 0.405 |
| Lip | 244 | 1.37 [0.08,4.04] | 0.50 [0.12,1.26] | 1.05 [0.59,1.69] | 1.16 [0.74,1.62] | 0.74 [0.52,0.97] | 0.00 |  | 0.686 | 0.863 | 0.690 |
| Hodgkin's disease | 239 | 1.94 [0.67,4.99] | 0.69 [0.12,2.80] | 0.99 [0.50,1.67] | 0.62 [0.35,0.94] | 0.91 [0.65,1.18] | 0.00 |  | 0.625 | 0.933 | 0.916 |
| Testis | 222 | **3.52 [1.18,7.37]** | 0.99 [0.09,3.37] | 1.81 [0.89,4.78] | **1.86 [1.16,3.04]** | 1.21 [0.83,1.73] | na^c^ |  | 0.01076 | 0.09278 | 0.00647 |
| Larynx | 208 | 0/2008^d^ | 0.27 [0.01,1.85] | 0.76 [0.29,1.52] | 0.81 [0.48,1.32] | 1.05 [0.77,1.43] | 0.00 |  | 0.973 | 0.927 | 0.752 |
| ^a^ Shown are the estimated risk ratios (RR) with 90% confidence intervals (CI) for 1st to 5th degree (1º-5º) relatives and mates of the 27 cancer sites with ≥200 cases, in bold when the 90% CI does not include 1.00, which corresponds to one-sided p-value <0.05. Also shown are combined p-values (one-sided) to evaluate the significance of the RR for all relatives (1^st^-5^th^ degree) and for close (1^st^ and 2^nd^ degree) and distant relatives (3^rd^-5^th^ degree). | | | | | | | | | | | |
| ^b^ NHL, non-Hodgkin's lymphoma. | | | | | | | | | | | |
| ^c^ na, not applicable (sex-specific cancers). | | | | | | | | | | | |
| ^d^ Number of mates or relatives with cancer/total number of mates or relatives for each cancer site | | | | | | | | | | | |

| **Table 28** RR estimates for relatives and mates of patients diagnosed with laryngeal cancer for sites with 200 or more cases^a^ | | | | | | | | | | | |
| --- | --- | --- | --- | --- | --- | --- | --- | --- | --- | --- | --- |
| Cancer sites | # of aff. | RR [90% CI] | | | | | |  | combined p-value for relatives | | |
|  |  | 1º relative | 2º relatives | 3º relatives | 4º relatives | 5º relatives | Mates |  | 1º-5º | 1º-2º | 3º-5º |
| Breast | 3812 | 1.00 [0.72,1.34] | 0.82 [0.62,1.02] | **1.17 [1.01,1.31]** | 0.98 [0.88,1.06] | 1.08 [1.00,1.15] | 1.77 |  | 0.419 | 0.401 | 0.046 |
| Prostate | 3380 | 0.94 [0.72,1.22] | 0.91 [0.69,1.14] | 0.85 [0.71,0.99] | 0.94 [0.83,1.04] | 1.03 [0.96,1.10] | 0.67 |  | 0.938 | 0.944 | 0.936 |
| Lung | 2904 | **2.03 [1.61,2.53]** | 1.11 [0.88,1.41] | 1.04 [0.88,1.19] | 1.00 [0.89,1.11] | 0.94 [0.88,1.02] | 1.73 |  | 0.00165 | 0.44915 | 0.6282 |
| Stomach | 2890 | **1.44 [1.10,1.88]** | 0.97 [0.74,1.22] | **1.31 [1.12,1.47]** | 0.94 [0.84,1.04] | 1.04 [0.94,1.10] | 1.23 |  | 0.025 | 0.126 | 0.051 |
| Colon | 2224 | 0.82 [0.52,1.20] | 1.27 [0.95,1.57] | 0.99 [0.81,1.17] | 0.89 [0.77,1.02] | 1.01 [0.91,1.09] | 0.56 |  | 0.636 | 0.433 | 0.850 |
| Bladder | 1384 | 1.15 [0.74,1.74] | 0.94 [0.63,1.40] | 0.87 [0.69,1.11] | 1.05 [0.91,1.26] | 1.05 [0.95,1.17] | 1.63 |  | 0.347 | 0.452 | 0.387 |
| Kidney | 1227 | 1.26 [0.81,1.97] | 1.08 [0.75,1.54] | 1.16 [0.90,1.42] | 0.79 [0.63,0.95] | **1.18 [1.06,1.30]** | 0.59 |  | 0.156 | 0.254 | 0.271 |
| Thyroid | 957 | 0.38 [0.13,1.02] | 0.87 [0.55,1.32] | 0.90 [0.63,1.17] | 0.96 [0.77,1.13] | **1.27 [1.11,1.42]** | 0.67 |  | 0.802 | 0.454 | 0.270 |
| Pancreas | 930 | **1.73 [1.08,2.74]** | 0.79 [0.46,1.26] | 1.14 [0.86,1.44] | 0.94 [0.75,1.13] | 0.93 [0.81,1.07] | 2.11 |  | 0.312 | 0.780 | 0.610 |
| Ovary | 906 | 0.76 [0.35,1.38] | 1.11 [0.77,1.79] | 1.24 [0.99,1.65] | 1.03 [0.85,1.26] | 0.95 [0.82,1.08] | 1.46 |  | 0.355 | 0.163 | 0.208 |
| Non-melanoma skin | 781 | 1.67 [0.92,2.53] | 0.61 [0.32,1.10] | 0.76 [0.50,1.02] | 1.20 [0.96,1.43] | 1.13 [0.97,1.28] | 0.00 |  | 0.312 | 0.697 | 0.344 |
| Rectum | 767 | 1.13 [0.56,2.11] | 1.04 [0.57,1.50] | 1.01 [0.75,1.35] | 0.80 [0.61,0.99] | 1.00 [0.88,1.17] | 1.69 |  | 0.644 | 0.740 | 0.787 |
| Endometrium | 753 | 1.26 [0.76,2.30] | 0.65 [0.32,1.16] | 1.14 [0.84,1.48] | 0.93 [0.74,1.15] | 0.85 [0.72,0.99] | 0.00 |  | 0.751 | 0.927 | 0.762 |
| Cervix uteri | 724 | **2.19 [1.37,3.82]** | 1.31 [0.82,2.01] | 1.13 [0.81,1.53] | 0.95 [0.74,1.18] | 1.15 [0.99,1.32] | 1.95 |  | 0.0053 | 0.08925 | 0.1668 |
| Brain | 663 | 1.00 [0.42,2.08] | 0.20 [0.03,0.54] | 1.31 [0.94,1.69] | 0.96 [0.73,1.20] | 1.05 [0.89,1.20] | 2.47 |  | 0.799 | 0.830 | 0.200 |
| Melanoma of skin | 618 | 0.91 [0.41,1.92] | 1.14 [0.65,1.89] | 1.16 [0.81,1.61] | 0.89 [0.64,1.09] | 1.00 [0.81,1.13] | 0.00 |  | 0.544 | 0.518 | 0.659 |
| Esophagus | 535 | 1.46 [0.66,2.75] | 0.59 [0.26,1.25] | 1.04 [0.71,1.48] | 1.10 [0.85,1.38] | 1.02 [0.84,1.20] | 1.58 |  | 0.435 | 0.656 | 0.313 |
| Diffuse NHL^b^ | 422 | 0/2008^d^ | 0.49 [0.15,1.14] | 0.96 [0.62,1.47] | 0.90 [0.62,1.18] | 1.04 [0.87,1.28] | 0.00 |  | 0.981 | 0.845 | 0.583 |
| Multiple myeloma | 391 | 0.82 [0.29,3.04] | 0.52 [0.16,1.47] | 0.66 [0.42,1.10] | 0.95 [0.68,1.30] | 0.85 [0.68,1.06] | 1.61 |  | 0.955 | 0.969 | 0.940 |
| Lymphoid leukaemia | 368 | 1.50 [0.58,4.27] | **1.84 [1.10,3.36]** | 0.81 [0.47,1.44] | 1.29 [0.93,1.75] | 1.23 [0.99,1.45] | 2.77 |  | 0.024 | 0.027 | 0.155 |
| Myeloid leukaemia | 342 | 1.42 [0.55,4.12] | 1.18 [0.60,2.38] | 0.94 [0.54,1.59] | 1.15 [0.82,1.57] | 0.88 [0.65,1.09] | 4.49 |  | 0.335 | 0.463 | 0.575 |
| Meninges | 291 | 1.57 [0.50,4.39] | 1.60 [0.74,2.77] | 0.93 [0.50,1.62] | 0.90 [0.58,1.29] | 1.19 [0.92,1.47] | 0.00 |  | 0.158 | 0.251 | 0.443 |
| Liver | 257 | **3.46 [1.76,7.29]** | 1.03 [0.40,2.02] | **1.79 [1.16,2.65]** | 1.34 [0.94,1.91] | 0.84 [0.61,1.09] | 0.00 |  | 0.0029 | 0.11455 | 0.05175 |
| Lip | 244 | 0.90 [0.21,3.19] | 0.78 [0.26,1.68] | 1.03 [0.55,1.79] | 0.87 [0.52,1.28] | 0.78 [0.54,1.02] | 0.00 |  | 0.870 | 0.890 | 0.864 |
| Hodgkin's disease | 239 | 1.34 [0.28,3.98] | 1.89 [0.95,3.18] | 1.31 [0.74,2.14] | 1.14 [0.69,1.65] | 0.86 [0.61,1.13] | 0.00 |  | 0.084 | 0.095 | 0.342 |
| Testis | 222 | 0/2008^d^ | 0.27 [0.01,1.85] | 0.76 [0.29,1.52] | 0.81 [0.48,1.32] | 1.05 [0.77,1.43] | 0.00 |  | 0.973 | 0.927 | 0.752 |
| Larynx | 208 | **3.02 [1.06,6.65]** | 0.65 [0.05,2.71] | 0.27 [0.02,0.90] | 1.30 [0.75,2.33] | 0.83 [0.53,1.28] | 0.00 |  | 0.3358 | 0.14907 | 0.76038 |
| ^a^ Shown are the estimated risk ratios (RR) with 90% confidence intervals (CI) for 1st to 5th degree (1º-5º) relatives and mates of the 27 cancer sites with ≥200 cases, in bold when the 90% CI does not include 1.00, which corresponds to one-sided p-value <0.05. Also shown are combined p-values (one-sided) to evaluate the significance of the RR for all relatives (1^st^-5^th^ degree) and for close (1^st^ and 2^nd^ degree) and distant relatives (3^rd^-5^th^ degree). | | | | | | | | | | | |
| ^b^ NHL, non-Hodgkin's lymphoma. | | | | | | | | | | | |
| ^c^ na, not applicable (sex-specific cancers). | | | | | | | | | | | |
| ^d^ Number of mates or relatives with cancer/total number of mates or relatives for each cancer site | | | | | | | | | | | |
